# Genetic diversity for drought tolerance in the native forage grass *Trichloris crinita* and possible morpho-physiological mechanisms involved

**DOI:** 10.3389/fpls.2023.1235923

**Published:** 2023-08-03

**Authors:** Deolindo Luis Esteban Dominguez, Juan Bruno Cavagnaro, Juana Panasiti Ros, Anh Tuan Le, Yong Suk Chung, Pablo Federico Cavagnaro

**Affiliations:** ^1^ Instituto de Biología Agrícola de Mendoza (IBAM), Facultad de Ciencias Agrarias, Consejo Nacional de Investigaciones Científicas y Técnicas (CONICET), Universidad Nacional de Cuyo, Lujan de Cuyo, Mendoza, Argentina; ^2^ Facultad de Ciencias Agrarias, Universidad Nacional de Cuyo, Lujan de Cuyo, Mendoza, Argentina; ^3^ Department of Plant Resources and Environment, Jeju National University, Jeju, Republic of Korea; ^4^ Consejo Nacional de Investigaciones Científicas y Técnicas (CONICET), Instituto Nacional de Tecnología Agropecuaria (INTA) Agricultural Experimental Station Mendoza (EEA Mendoza), Lujan de Cuyo, Mendoza, Argentina

**Keywords:** *Leptochloa crinita*, drought stress, photoassimilates partitioning, biomass, stomatal conductance, photochemical efficiency

## Abstract

**Introduction:**

The use of drought tolerant genotypes is one of the main strategies proposed for coping with the negative effects of global warming in dry lands. *Trichloris crinita* is a native forage grass occupying extensive arid and semi-arid regions in the American continent, and used for range grazing and revegetation of degraded lands.

**Methods:**

To identify drought-tolerant genotypes and possible underlying physiological mechanisms, this study investigated drought tolerance in 21 genetically diverse *T. crinita* genotypes under natural field conditions. The accessions were grown under irrigated (control) and drought conditions for 84 days after initiation of the drought treatment (DAIDT), which coincided with flowering initiation. Various morpho-physiological traits were monitored, including total-, foliage-, and root biomass yield, dry matter partitioning to individual plant organs (roots, leaves, stems, and panicles), total leaf area, chlorophyll content, photochemical efficiency of photosystem II, stomatal conductance, and number of panicles per plant.

**Results and discussion:**

Broad and significant variation (p<0.001) was found among the accessions for all the traits. Three highly tolerant and three very sensitive accessions were identified as the most contrasting materials, and their responses to drought stress were confirmed over two years of experiments. Under prolonged drought conditions (84 DAIDT), the tolerant accessions were generally more productive than the rest for all the biomass yield components analyzed, and this was associated with a postponed and more attenuated decrease in variables related to the plant photosynthetic activity, such as stomatal conductance, chlorophyll content, and photochemical efficiency. In contrast to previous findings, our data indicate no direct relationship between drought tolerance and the level of aridity in the accessions natural habitats, but rather suggest genetic heterogeneity and ample variation for drought tolerance in *T. crinita* natural populations derived from a particular location or environment. Also, having low total and forageable biomass yield, or increased biomass allocation to the roots (i.e., lower foliage/root ratio), under optimal water availability, were not associated with greater drought tolerance. The drought-tolerant accessions identified are of value for future genetic research and breeding programs, and as forage for range grazing and revegetation in arid regions.

## 1 Introduction

Drylands cover ~41% of the Earth’s land surface and will experience substantial expansion, degradation, and conversions among dryland subtypes under the predicted climate change scenarios (Reynolds et al., 2007; Yao et al., 2020). Such scenarios predict more frequent and intense drought periods in many regions, further aggravating the situation of arid lands (Overpeck, 2013; International Panel of Climatic Changes, 2014). One of the strategies to counteract land degradation problems is to reseed degraded areas with species capable of surviving in these ecosystems. Native species from arid environments are potentially valuable genetic resources for revegetation and rehabilitation of degraded drylands (Villagra et al., 2021). In this context, the selection of drought-tolerant species, and genotypes within a species, along with the use of adequate and sustainable management practices, are critical for a successful revegetation program with native species. *Trichloris crinita* (Lag.) Parodi [syn. *Leptochloa crinita* (Lag.) Peterson and Snow (Peterson et al., 2012; Peterson et al., 2015)] (Chloridoideae, Poaceae) is a perennial grass native to arid and semi-arid regions of North and South America (Quiroga et al., 2018). In these dry lands, range grazing is one of the few non-irrigated agricultural activities, with native grasses being the main forage resource (Busso and Fernández, 2018). Because of its good forage quality (Dominguez et al., 2022), drought tolerance, resistance to trampling and grazing, and rapid growth and competing aggressiveness among other native species (reviewed by Kozub et al., 2018b), *T. crinita* is widely promoted for range grazing and restoration of degraded rangelands in environments with low water availability (Passera et al., 1992; Cavagnaro and Trione, 2007; Guevara et al., 2009; Kozub et al., 2018b; USDA-NRCS, 2020). Despite the general drought tolerance attributed to *T. crinita*, as compared to other native forage grasses, it must be noted that this species grows naturally in vast geographical regions varying widely in precipitation regimes and overall water availability, with annual precipitations ranging from 150 to 1500 mm, and phenotypically-distinct ecotypes and populations can be found across these environments (Marinoni et al., 2015; Quiroga et al., 2018). This suggests that different adaptive mechanisms for water utilization and coping with drought stress may exist in the *T. crinita* germplasm. Such hypothesis has not been explored to date.

Because of its importance in arid regions, the Germplasm Bank of Native Grasses (GBNG) at the Argentine Institute for Research in Arid Regions (IADIZA) is dedicated to the collection, preservation, studying, and distribution of germplasm of native grasses from arid and semiarid regions of Argentina. In the last decades, representative plants from natural populations of *T*. *crinita* and other native grasses were collected from the Monte phytogeographical region, located in the west of Argentina, and evaluated at various levels. These studies revealed broad genetic diversity for this germplasm collection at the DNA level (using AFLP and SSR molecular markers), as well as for morphological, cytological, physiological, agronomic and forage-quality traits (Greco and Cavagnaro, 2005; Cavagnaro et al., 2006; Kozub et al., 2018a; Kozub et al., 2019; Dominguez et al., 2022). However, to date, no thorough evaluation of drought tolerance in the GBNG *T. crinita* collection has been performed. The characterization of these germplasm with regards to their drought-stress responses may reveal tolerant genotypes, of potential value for revegetation and forage grazing in extremely arid environments.

To cope with drought, plants of arid ecosystems have developed a range of physiological and morphological strategies, including the development of low specific leaf area (SLA), increased allocation of photoassimilates to the roots, reduced foliar biomass, reduced stomatal conductance, low photosynthetic rate, reduced transpiration rate and water loss, osmotic adjustment, low relative growth rate, and reduction of maximum quantum efficiency of photosystem II (Fv/Fm) (Poorter and Nagel, 2000; Chesson et al., 2004; Blum, 2005; Greco and Cavagnaro, 2005; Campanella and Bertiller, 2008; Manzoni et al., 2011; Acquaah, 2012; Poorter et al., 2012; Quiroga et al., 2013; Carrizo et al., 2020; Marinoni et al., 2020). Variations in some of these physiological traits have been shown to condition the growth and survival of various grass species when grown in arid environments.

Only a few studies have investigated drought tolerance in *T. crinita*. First, Greco and Cavagnaro (2003) evaluated three *T. crinita* genotypes under irrigated and drought conditions, reporting significant differences in drought tolerance among the genotypes, and suggested the development of higher root biomass as a possible adaptive trait associated with drought tolerance. Later, Quiroga et al. (2013) compared two ecotypes from contrasting environments varying in rainfall precipitations finding that, under drought conditions, the ecotype from the more-extreme arid site exhibited greater drought-tolerance than the ecotype from the region with greater water availability. Similarly, Marinoni et al. (2020) evaluated four ecotypes under hydroponics in a growth chamber, concluding that ecotypes of arid and semi-arid origins were more tolerant to drought than ecotypes from humid sites. Although these studies evidenced the existence of variation for drought tolerance in *T. crinita*, they were performed using very few (2-4) genotypes, under controlled conditions (in pots or hydroponics), in a single environment (i.e., one year and location), and analyzed a few morphological traits as response variables. Thus, a more comprehensive characterization of the drought-stress response –e.g., by means of analysis of various morphological and physiological traits- in a large and genetically diverse *T. crinita* collection, performed under replicated and natural field conditions, is required to: 1) have a robust assessment on the extent of genetic variation for drought tolerance in the *T. crinita* germplasm; 2) identify new drought-resistant genotypes that combine other desirable agronomic and forage quality traits; and 3) identify morpho-physiological mechanisms associated with drought tolerance in this species.

Based on the background information described above, we hypothesize that: 1) there is ample genetic variability for drought tolerance in the *T. crinita* germplasm, which will allow the identification and selection of drought-tolerant genotypes; 2) accessions from extremely arid regions have greater drought tolerance than accessions from less arid regions; and 3) variation in morpho-physiological traits explain the differences in drought tolerance found among *T. crinita* accessions. Thus, the present study characterized drought tolerance in a genetically diverse *T. crinita* germplasm collection, using a two-year partially replicated field experiment. To this end, 21 *T. crinita* accessions from the GBNG grown under irrigated and drought conditions were analyzed for morpho-physiological and productive (forage biomass) variables, with the ultimate goal of identifying highly-resistant genotypes and physiological mechanisms underlying drought tolerance in this grass species.

## 2 Materials and methods

### 2.1 Plant materials

**Figure 1:**
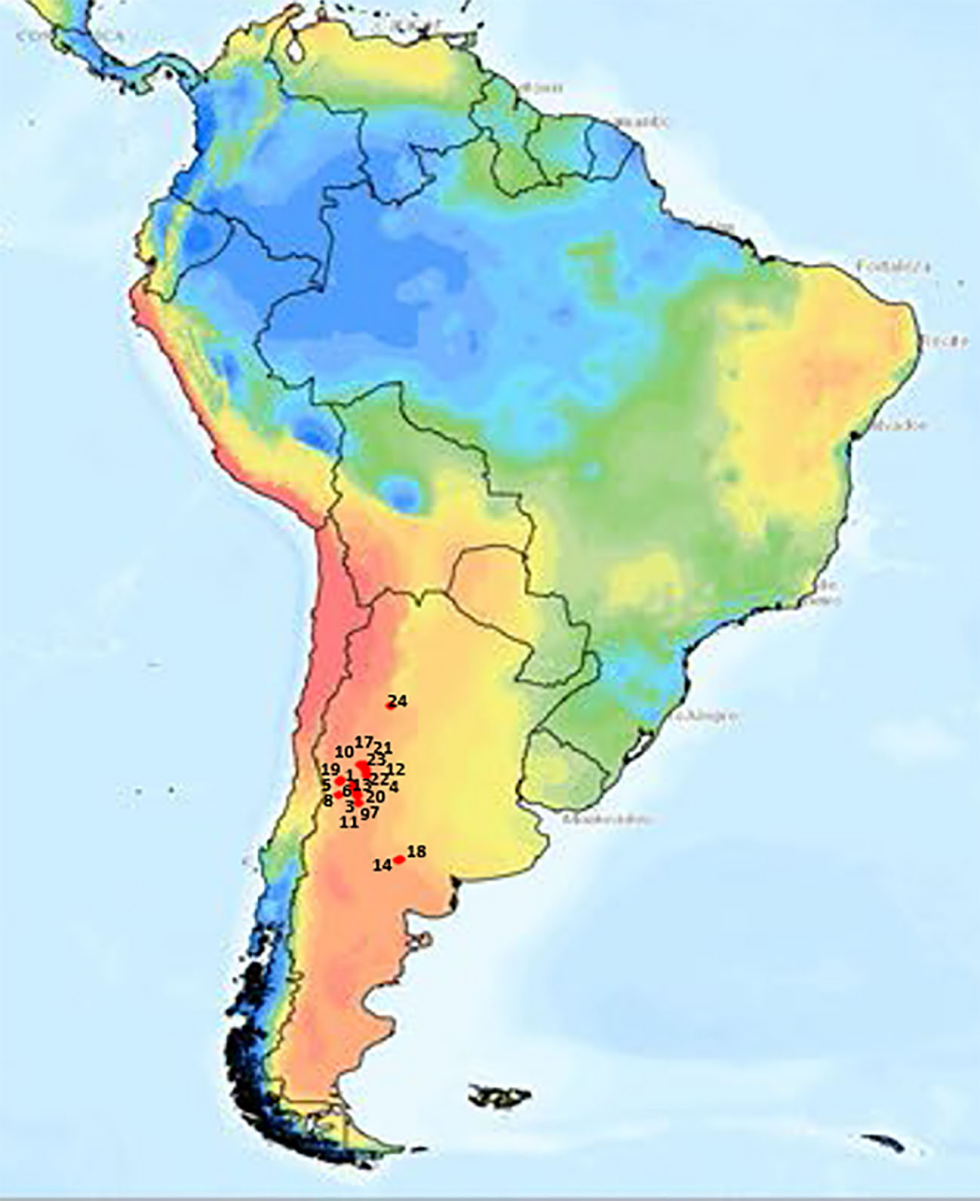
Geographical origin of 21 *Trichloris crinita* accessions (red dots) in South America, with Aridity Indices (Zomer et al., 2022) depicted in color.

Twenty-one *T. crinita* accessions from the GBNG, at IADIZA, were used. These materials were selected to maximize genetic diversity, from collections of representative plants from 48 natural populations of *T. crinita* dispersed throughout extensive arid and semi-arid regions (~350,000 km^2^) in Argentina. The selected accessions are genetically and morphologically diverse, and they constitute a representative sample of the broad phenotypic variation observed naturally in the Monte phytogeographical region (Cavagnaro et al., 2006). The geographic locations and main climatic and edaphic characteristics at the collection sites of the accessions are presented in **Table 1** and **Figure 1**. Noteworthy, these accessions are different from the plant materials used in previous studies by Quiroga et al. (2013) and Marinoni et al. (2020).

**Table 1:** *Trichloris crinita* accessions and main characteristics of their collection sites in Argentina.

Acc. ID	Province	Location	Latitude (° S)^ζ^	Longitude (° W)^ζ^	Altitude (m.a.s.l.)^ζ^	Annual mean temp (°C)^†^	Mean temp in GS (°C)^†^	Mean max temp in GS (°C)^†^	Mean min temp in GS (°C)^†^	Mean annual rainfall (mm)^¥^	Aridity index^β^		Soil electrical conductivity (µmhos/cm)^ζ^	Climate classification (abbreviation)^λ^
											A	B		
1	Mendoza	Rivadavia, El Mirador	33.33	68.25	653	16.9	22.5	30.2	14.8	204	7.6	0.13	348	Warm desert (BWk)
3	Mendoza	Santa Rosa, Ñacuñan	34.04	67.90	540	16.9	22.6	30.5	14.7	327	12.2	0.15	264	Cold semi-arid (BSk)
4	Mendoza	Lavalle, Arroyito	32.79	67.37	540	19.3	24.6	32.0	17.1	190	6.5	0.16	296	Cold desert (BWh)
5	Mendoza	Luján de Cuyo, Ugarteche	33.21	68.95	950	16.2	21.5	28.8	14.2	247	9.4	0.14	1176	Warm desert (BWk)
6	Mendoza	Santa Rosa, Comte. Salas	33.83	68.00	530	16.5	22.2	30.2	14.2	327	12.3	0.15	227	Cold semi-arid (BSk)
7	Mendoza	Santa Rosa, Ñacuñán	34.04	67.90	540	16.9	22.6	30.5	14.7	327	12.2	0.15	361	Cold semi-arid (BSk)
8	Mendoza	San Carlos, Pareditas	33.96	69.04	940	14.4	19.4	27.7	11.0	334	13.7	0.20	342	Cold semi-arid (BSk)
9	Mendoza	Santa Rosa, Ñacuñán	34.04	67.90	540	16.9	22.6	30.5	14.7	327	12.2	0.15	281	Cold semi-arid (BSk)
10	San Juan	25 de mayo, El Encón	32.19	67.71	520	19.2	24.8	32.3	17.3	104	3.6	0.16	7825	Cold desert (BWh)
11	Mendoza	San Rafael, Guadales	34.49	67.83	606	16.3	21.9	30.2	13.5	334	12.7	0.16	293	Cold semi-arid (BSk)
12	Mendoza	Lavalle, El Retamo	32.51	67.41	525	19.4	24.7	32.0	17.4	190	6.5	0.16	2357	Cold desert (BWh)
13	Mendoza	Santa Rosa, Pichi Ciego	33.57	68.09	530	16.6	22.2	30.1	14.4	327	12.3	0.14	424	Cold semi-arid (BSk)
14	La Pampa	La Asturiana	37.83	65.36	260	15.2	20.5	29.1	11.9	519	20.6	0.20	1061	Cold semi-arid (BSk)
17	San Juan	25 de mayo, El Encón	32.15	67.51	520	19.3	24.8	32.3	17.3	104	3.6	0.18	1280	Cold desert (BWh)
18	La Pampa	Lihuel Calel, Sierra Chica	37.90	65.46	235	15.2	20.5	29.1	11.9	519	20.6	0.20	425	Cold semi-arid (BSk)
19	Mendoza	Luján de Cuyo, Agrelo	33.11	68.91	940	16.4	21.7	29.0	14.3	227	8.6	0.13	2050	Warm desert (BWk)
20	Mendoza	Santa Rosa, Pichi Ciego	33.57	68.09	530	16.6	22.2	30.1	14.4	327	12.3	0.14	424	Cold semi-arid (BSk)
21	San Juan	25 de mayo, El Encón	32.19	67.71	520	19.2	24.8	32.3	17.3	104	3.6	0.16	7825	Cold desert (BWh)
22	Mendoza	Lavalle, Arroyito	32.79	67.37	510	19.3	24.6	32.0	17.1	190	6.5	0.16	296	Cold desert (BWh)
23	Mendoza	Lavalle, El Retamo	32.47	67.42	525	19.5	24.8	32.1	17.4	190	6.4	0.17	3585	Cold desert (BWh)
24	Catamarca	Capayán, Miraflores	28.65	65.91	460	20.4	25.3	31.9	18.7	411	13.5	0.23	241	Warm semi-arid (BSh)

GS. growing season (spans the months of October to March, corresponding to spring-summer in the Southern hemisphere); m.a.s.l. meters above sea level.

^ζ^ Data from accession passport data for soil analysis performed at the Germplasm Bank of Native Grasses (GBNG) at the Argentine Institute for Research in Arid Regions (IADIZA). ^†^ Data source: WorldClim v2.1 (https://www.worldclim.org/data/worldclim21.html#; Fick and Hijmans, 2017). Historical climate data (1970–2000) were extracted from layers of 30 arc-second of resolution (≈ 1 km). ^¥^ Data from closest weather station to the collection sites (<20 km). ^β^ Aridity indices were calculated according to De Martonne (1926) (A) and Zomer et al. (2022) (B). For both indices, greater values indicate lower aridity. ^λ^ According to the Köppen–Geiger climate classification.

### 2.2 Experimental design

The field study consisted of a partially-replicated experiment, conducted during the 2017-2018 growing season (henceforth “2018”), in which 21 *T. crinita* accessions were evaluated. The experiment was repeated in the following season (2018-2019; henceforth “2019”) with the six most contrasting accessions with regards to their drought-tolerance performance in the previous year. The climatic conditions at the site of the experiment for 2018 and 2019 trials are presented in **Supplementary Figure S1**. For implantation, individual seeds were sown in 250 cm^3^ pots with sterile soil, and plants were grown under greenhouse conditions until they had 5–6 leaves, after which the pots were placed outdoors for a period of rustication, and after one week they were transplanted to the experimental field of the Faculty of Agricultural Sciences, Nacional University of Cuyo (Mendoza, Argentina) (33° S, 68° 8’ O). In the field, the experimental design consisted of two plots, one for the irrigated (control) and one for the drought treatment, separated from each other by five meters, with each plot containing 252 *T. crinita* plants, for a total of 504 plants used in the experiment. All the plants in both treatment plots were drip-irrigated to field capacity during the first 80 days after the transplant, to ensure that the plants were fully established in the field. After the 80^th^ day, and coincidently with flowering initiation, the drought treatment was imposed in one of the plots by completely eliminating irrigation, while the control plot continued with the same irrigation regime throughout the experiment. A 21 × 2 factorial design with divided plots (subplots) was used, being the two plots the drought and control treatments, and the 21 subplots the accessions within each plot. Each subplot consisted of 12 plants of each *T. crinita* accession, with a completely randomized distribution in the field (within each plot) with adjacent plants separated 80 cm from each other. To prevent any water from precipitations to infiltrate the ground, the soil of the entire experimental plot in both treatments, drought and irrigated, was covered with a 200-µm thick polyethylene, and covered with soil on top. By this means, water availability for the plants was completely controlled, and provided only by the drip irrigation system. **Supplementary Figure S2** shows the field trial for evaluating drought tolerance in *T. crinita* accessions.

### 2.3 Sampling and measurements of response variables

Plants of all the accessions were harvested at four different sampling times during the vegetative cycle; at the beginning of the drought treatment (day 0), and 28, 56 and 84 days after initiation of the drought treatment (DAIDT). At each sampling date, three plants per accession were harvested in both the irrigated and drought plots. The aerial and underground parts of each plant were independently collected. The underground plant parts were obtained from a volume of soil of 0.064 m^3^ (0.4 m × 0.4 m × 0.4 m) by excavation around the plant to a depth of 40 cm, considering that the majority of the root system in this species is found within this soil volume (Greco and Cavagnaro, 2005). The roots were then separated from the soil by rinsing with water and sieving through a 0.6 mm mesh screen, and dried in an oven at 60°C until constant weight, to obtain their dry matter (DM) content. The aerial part of the plant, henceforth referred to as “foliage”, was subdivided into its components leaf blades (from the ligule to the leaf tip, henceforth referred to as “leaves”), ‘stems+culms’ (henceforth “stems”), and panicles, and oven-dried at 60°C until constant weight, to estimate their respective dry matter contents. Dry matter partitioning to each of the plant parts (i.e., leaves, stems, panicles, and roots) was calculated by dividing the DM of a particular plant part by the total DM of the plant, and expressed as percentage of the total plant biomass. The ratio of ‘foliage DM/root DM’, referred to as ‘foliage/root ratio’ (FRR), was calculated for each plant based on its DM contents in the foliage and roots. Total leaf area per plant was determined, prior to desiccation of the leaf samples in the oven, using a leaf area meter (LICOR, Model LI3000A. USA).

On a weekly basis and until the 84 DAIDT, the number of panicles per plant was recorded; and stomatal conductance, chlorophyll index, and maximum quantum efficiency of photosystem II (PSII) were determined on the fully expanded penultimate leaf of each plant, between 10 am and 2 pm. For each accession and treatment, three plants (i.e., three biological replicates) were measured at each time point. Stomatal conductance (g_s_) was measured with a leaf porometer (Decagon Devices, Model SC-1, USA) on the surface of the abaxial side of the leaves, avoiding the midrib. Mean values of three readings per plant, for three plants per accession and treatment, at each time point, were considered to calculate mean g_s_ values. The chlorophyll index was determined using a chlorophyll meter (Minolta, SPAD 502 Plus, Japan). For each plant, the mean of three readings was obtained, and three plants per accession and treatment were analyzed at each time point. Maximum quantum efficiency of PSII (Fm/Fv) was determined by measurements of chlorophyll *a* fluorescence using a Plant Efficiency Analyzer (Pocket PEA; Hansatech Instruments, England). Prior to the measurements, the leaves were fully dark-adapted for 30 min to achieve the complete oxidation of the primary electron carriers. Chlorophyll fluorescence induction was prompted by a 3-s pulse of red light (peak wavelength of 627 nm) emitted from a LED lamp filtered by a NIR filter. This pulse was emitted at maximal saturation irradiance of 3500 μmol m^−2^ s^−1^. The basal non-variable chlorophyll fluorescence (F_0_) and the maximum fluorescence induction (Fm) were determined, and the variable fluorescence (Fv) was calculated as follows: Fv = Fm - F_0_. Then, the maximum quantum efficiency of PSII (Fv/Fm) was estimated according to Maxwell and Johnson (2000).

In order to reflect the degree of change in the plant parameters analyzed due to the drought stress, the data for all the variables were expressed as percentage (%) relative to the values in the respective control treatments, according to the formula (Value_Drought/_Value_Control_) × 100, unless otherwise stated.

### 2.4 Statistical and principal component analyses

The data were analyzed using analysis of variance (ANOVA) by mixed linear models with a factorial structure, treating drought treatment, accession, and their interactions, as fixed effects, while biological replicates were treated as random effects. Different structures of residual variance were evaluated, and the best models were selected using the Akaike (AIC) and Schwarz (BIC) information criteria (Di Rienzo et al., 2017). Prior to the analysis, percentage values were transformed by the square root of the bow-sine function. All the statistical and graphic analyses were performed with the software InfoStat version 2020 (Di Rienzo et al., 2020) and R^®^ version 3.5.3 (R Core Development Team). Means comparisons were performed using the DGC test (Di Rienzo et al., 2002). For all the variables, the data were expressed as mean value ± standard deviation and p-values ​​< 0.05 were considered significant. Principal component analysis (PCA) was implemented in the InfoStat software to classify the accessions based on drought-response variables using a data matrix of 21 × 9, where the rows represent the 21 *T. crinita* accessions and the columns comprised the data for nine morpho-physiological traits.

## 3 Results

### 3.1 Total biomass yield

Total plant biomass (i.e., DM of the four plant parts analyzed combined), expressed as percentage relative to the total DM content in control plants, henceforth referred as “RTDM”, was influenced by the accession, year of cultivation, sampling time, and their interactions (**Table 2**). In 2018, ample and significant differences (p<0.001) were found among *T. crinita* accessions and among sampling times (i.e., 0, 28, 56 and 84 DAIDT) for RTDM (**Table 3**). As expected, before initiation of the drought treatment (0 DAIDT), all the accessions had values close to 100% and they were statistically comparable, indicating no substantial differences between the two treatment plots due to factors unrelated to the drought treatment. After the drought treatment was imposed, all the accessions were significantly and differentially affected by the drought stress at the second sampling date (28 DAIDT), as evidenced by the broad variation found for RTDM values, ranging from 37.4% (in acc. 5) to 87.6% (acc. 21). As the drought treatment progressed, RTDM values were further reduced, with mean values at the end of the drought treatment in the range of 21.6% (acc. 5) to 83.3% (acc. 3). For the last two sampling dates, eight statistically different groups of accessions were revealed for this trait (**Table 3**). Together, the nearly four-fold difference in RTDM between the least and the most affected accessions, and the large number of statistically different groups found for this trait, suggest broad genetic variation for drought tolerance in the *T. crinita* germplasm.

**Table 2:** Influence of the accession, sampling time, year of cultivation, and their interactions on the relative values (%) of total dry matter (RTDM), foliage dry matter (RFDM), roots dry matter (RRDM), foliage/root ratio (RFRR), leaf area (RLA), chlorophyll index (RCI), photochemical efficiency (RPE), stomatal conductance (Rg_s_), and number of panicles per plant (RNPP) of *T. crinita* accessions grown under drought conditions in 2018 and 2019.

Year (N° accs)	Factor	RTDM (n=252)	RFDM (n=252)	RRDM (n=252)	RFRR (n=252)	RPE (n=819)	RLA (n=252)	RCI (n=819)	RPE (n=819)	Rg_s_ (n=819)	RNPP (n=819)
2018 (21 accs.)	Accession (A)	146.8***	1350.9***	916.2***	45.6***	47.8***	20.3***	66.3***	47.8***	47.8***	320.1***
	Sampling time (T)	2176.3***	142.6***	47.4***	63.0***	837.8***	2819.4***	2771***	837.8***	1734.2***	159.1***
	A x T	20.7***	20.8***	8.5***	10.5***	9.7***	9.27***	7.8***	9.7***	13.9***	18.3***
2018 and 2019 (6 accs.)		RTDM (n=144)	RFDM (n=144)	RRDM (n=144)	RFRR (n=144)	RPE (n=468)	RLA (n=144)	RCI (n=468)	RPE (n=468)	Rg_s_ (n= 468)	RNPP (n= 468)

	Year (Y)	66.3***	68.6***	ns	19.8***	21.5***	ns	64.2***	21.5***	13.0**	222.2***
	Accession (A)	257.8***	235.1***	54.2***	36.2***	468.5***	21.5***	310.3***	468.5***	142.6***	1563.1***
	Sampling time (T)	996.4***	773.0***	290.0***	8.9***	903.1***	1064.3***	1417.6***	903.1***	626.8***	321.2***
	Y x A	10.9***	10.9***	3.6**	7.00***	ns	4.1**	3.6**	ns	ns	148.5***
	Y x T	47.2***	33.7***	21.5***	ns	5.3***	5.6**	29.9***	5.3***	1.8*	5.4***
	A x T	69.6***	60.6***	15.5***	8.5***	72.1***	11.9***	15.8***	72.1***	38.9***	29.3***
	Y x A x T	7.3***	8.0***	ns	3.6***	2.3***	2.09*	4.7***	2.27***	ns	7.4***

Numbers are F values from ANOVA. Asterisks indicate statistically significant effects at p<0.05 (*), p<0.01 (**), and p<0.001 (***). ns, not significant. For the ANOVA, all the variables were expressed as percentage relative to the values in their respective irrigated controls. Twenty-one T. crinita accessions were evaluated in 2018, and the six most contrasting accessions with regards to drought-tolerance were re-evaluated in 2019 (i.e., the ANOVA of 2018 and 2019 comprised data for six accessions over two years).

**Table 3:** Time course variation for mean relative total dry matter (RTDM) content for *Trichloris crinita* accessions grown under drought conditions in 2018 and 2019.

Year	Accession	0 DAIDT	28 DAIDT	56 DAIDT	84 DAIDT
2018	1	95.4 ± 3.0 a	74.7 ± 6.4 c	79.6 ± 1.5 c	80.2 ± 0.5 c
	3	101.7 ± 2.5 a	72.0 ± 2.6 c	**81.8 ± 5.5 b**	**83.3 ± 1.6 b**
	4	98.9 ± 1.7 a	52.9 ± 1.2 f	56.9 ± 1.1 e	57.4 ± 2.3 e
	5	97.5 ± 9.6 a	***37.4 ± 0.8 g***	***27.6 ± 0.4 h***	***21.6 ± 0.6 i***
	6	97.8 ± 1.7 a	76.1 ± 1.8 c	69.6 ± 1.5 d	65.8 ± 4.6 d
	7	97.1 ± 2.8 a	76.3 ± 1.5 c	66.5 ± 1.4 d	68.3 ± 2.0 d
	8	103.3 ± 5.3 a	54.4 ± 3.1 f	50.0 ± 2.1 f	48.4 ± 2.0 f
	9	98.7 ± 2.4 a	68.0 ± 0.8 d	73.3 ± 1.2 c	77.8 ± 0.7 c
	10	101.6 ± 2.0 a	86.2 ± 0.7 b	65.8 ± 1.2 d	66.7 ± 1.2 d
	11	102.4 ± 3.6 a	86.5 ± 5.0 b	79.5 ± 3.4 c	76.5 ± 0.5 c
	12	97.2 ± 2.8 a	59.4 ± 1.5 e	52.4 ± 1.0 f	49.6 ± 3.0 f
	13	97.9 ± 2.1 a	65.0 ± 2.9 d	62.5 ± 0.7 d	63.7 ± 1.3 d
	14	98.0 ± 3.5 a	66.0 ± 1.0 d	66.4 ± 2.2 d	71.3 ± 1.6 c
	17	99.4 ± 2.1 a	71.4 ± 5.4 c	70.6 ± 2.7 c	71.2 ± 1.2 c
	18	101.7 ± 3.1 a	61.7 ± 2.2 d	52.9 ± 0.7 f	52.0 ± 2.8 f
	19	97.0 ± 2.0 a	78.4 ± 3.9 c	69.2 ± 1.3 d	68.4 ± 3.3 d
	20	96.7 ± 2.0 a	70.0 ± 1.6 d	65.3 ± 1.3 d	63.2 ± 2.2 d
	21	102.8 ± 2.9 a	**87.6 ± 1.2 b**	76.1 ± 1.8 c	74.7 ± 3.6 c
	22	96.6 ± 4.0 a	73.0 ± 1.9 c	62.5 ± 2.8 d	66.9 ± 1.7 d
	23	102.7 ± 3.9 a	86.4 ± 1.6 b	79.4 ± 0.9 c	79.0 ± 0.5 c
	24	98.4 ± 2.7 a	78.0 ± 3.2 c	73.5 ± 0.6 c	72.8 ± 2.6 c
2019	1	97.9 ± 1.2 a	61.3 ± 2.7 d	57.9 ± 2.4 e	84.2 ± 1.2 b
	3	99.4 ± 3.8 a	68.3 ± 1.5 c	**63.0 ± 2.6 d**	**90.1 ± 6.1 b**
	5	102.7 ± 4.7 a	***47.1 ± 5.9 f***	***19.2 ± 2.3 h***	***19.5 ± 2.7 h***
	9	97.1 ± 11.4 a	68.7 ± 3.6 c	53.0 ± 2.1 e	62.5 ± 1.2 d
	18	98.5 ± 1.0 a	70.9 ± 5.5 c	36.9 ± 1.4 g	45.6 ± 1.6 f
	22	101.5 ± 0.7 a	**82.1 ± 4.0 b**	42.7 ± 2.8 f	55.6 ± 1.8 e

RTDM values are the sum of DM of all the plant parts combined (i.e., roots, stems, leaves, and panicles) for plants in the drought treatment, expressed as percentage of the total DM content in the respective irrigated controls, according to the formula RTDM = (TDM_Drought_/TDM_Irrigated_) x 100. Data for 2018 (21 accessions) and 2019 (6 accessions) were analyzed independently. Values with the same letter are not significantly different at p ≤ 0.05. For each sampling time, the statistically most contrasting accessions were highlighted in color, depicting with dark and light blue the two statistical groups with the least affected accessions (i.e., the most drought-tolerant accessions), and with pink and red the statistical groups comprising the most affected accessions (i.e., the least tolerant accessions); whereas the greatest RTDM value is indicated in bold, and the lowest RDM in bold and italics. DAIDT, days after initiation of the drought treatment.

In general, RTDM was most-severely reduced in accessions 5, 8, 12, and 18, showing significantly lowest mean RTDM values at 56, and 84 DAIDT. In contrast, accessions 3, 1, 23, 9, 11, 21, 24, 14, and 17 exhibited the greatest mean RTDM values for these sampling times (**Table 3**). Although RTDM was significantly reduced in all the accessions at the first sampling time after the drought stress was imposed (i.e., 28 DAIDT), further variations for this trait differed, in rate and direction, for the different *T. crinita* accessions, as the drought treatment progressed. Thus, eleven accessions evidenced additional significant decreases in RTDM levels from 28 to 84 DAIDT, seven accessions did not vary significantly, and three accessions (accs. 1, 3, and 4) revealed significant increases in this time-frame (**Table 3**).

Based on these RTDM data, and results from the other drought-response variables analyzed in 2018 (described in sections below), the six most contrasting accessions with regard to drought tolerance were selected, considering accessions 1, 3, and 9 as the most drought-tolerant; and accessions 5, 18, and 22 as drought-sensitive. Accession 22, although its performance for RTDM was rather intermediate (**Table 3**), generally ranked among the most sensitive accessions for the other variables analyzed, along with accessions 5 and 18, therefore justifying its inclusion in the drought-sensitive group. These six selected accessions were re-evaluated in 2019, using the same methods and experimental procedures as in 2018.

Results for RTDM content in 2019 confirmed the data from the previous season. Thus, broad and significant variation (p<0.001) was found for this trait among the accessions, with accessions 1, 3, and 9 (considered as drought-tolerant) presenting significantly greater mean RTDM values than accessions 5, 18, and 22 (considered sensitive) for the last two time points of the drought treatment (**Table 3**). For these sampling times, and coincidently with results from 2018 using the complete set of 21 accessions, accessions 5 and 3 exhibited the lowest (19.5%) and greatest mean RTDM (90.1%) values, respectively.

### 3.2 Foliage/root ratio

**Figure 2:**
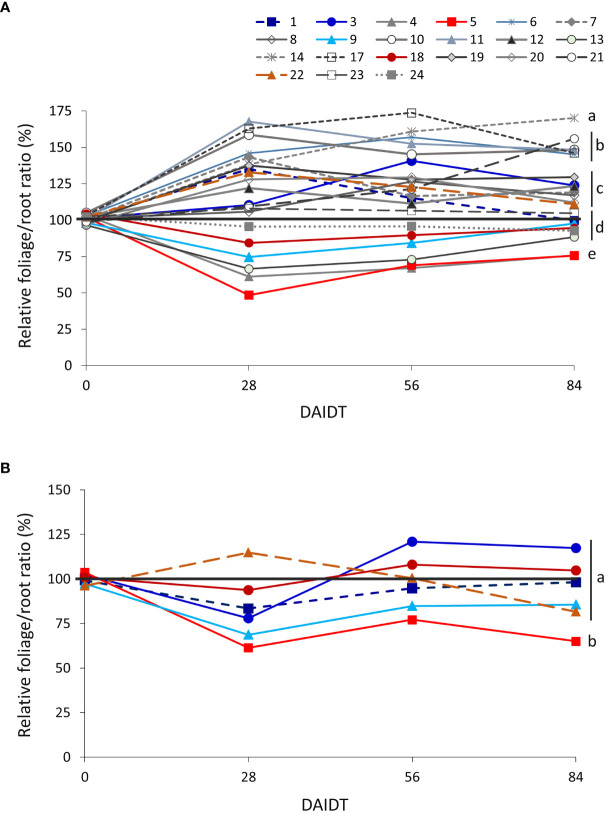
Time-course variation for relative foliage/root ratio (RFRR) for dry matter (DM) content in 21 *T. crinita* accessions grown under drought conditions in 2018 **(A)** and six contrasting accessions grown under the same conditions in 2019 **(B)**, over 84 days of drought stress. RFRR is expressed as percentage of the foliage/root ratio in the respective irrigated controls, as calculated by the formula: RFRR = [(FoliageDM/RootDM)_Drought/_(FoliageDM/RootDM)_Irrigated_] × 100. For each time point, the black horizontal line separates accessions that, under drought stress, allocated relatively more biomass to the roots (RFRR<100%) or to the foliage (RFRR>100%) in comparison to controls. Data points with different letters on the right indicate significantly different mean values at the end of the drought treatment at p<0.05 (DGC test), according to the means comparison analyses for all the accessions, time-points, and years presented in **Supplementary Table S3**. DAIDT. Days after initiation of the drought treatment.

**Figure 3:**
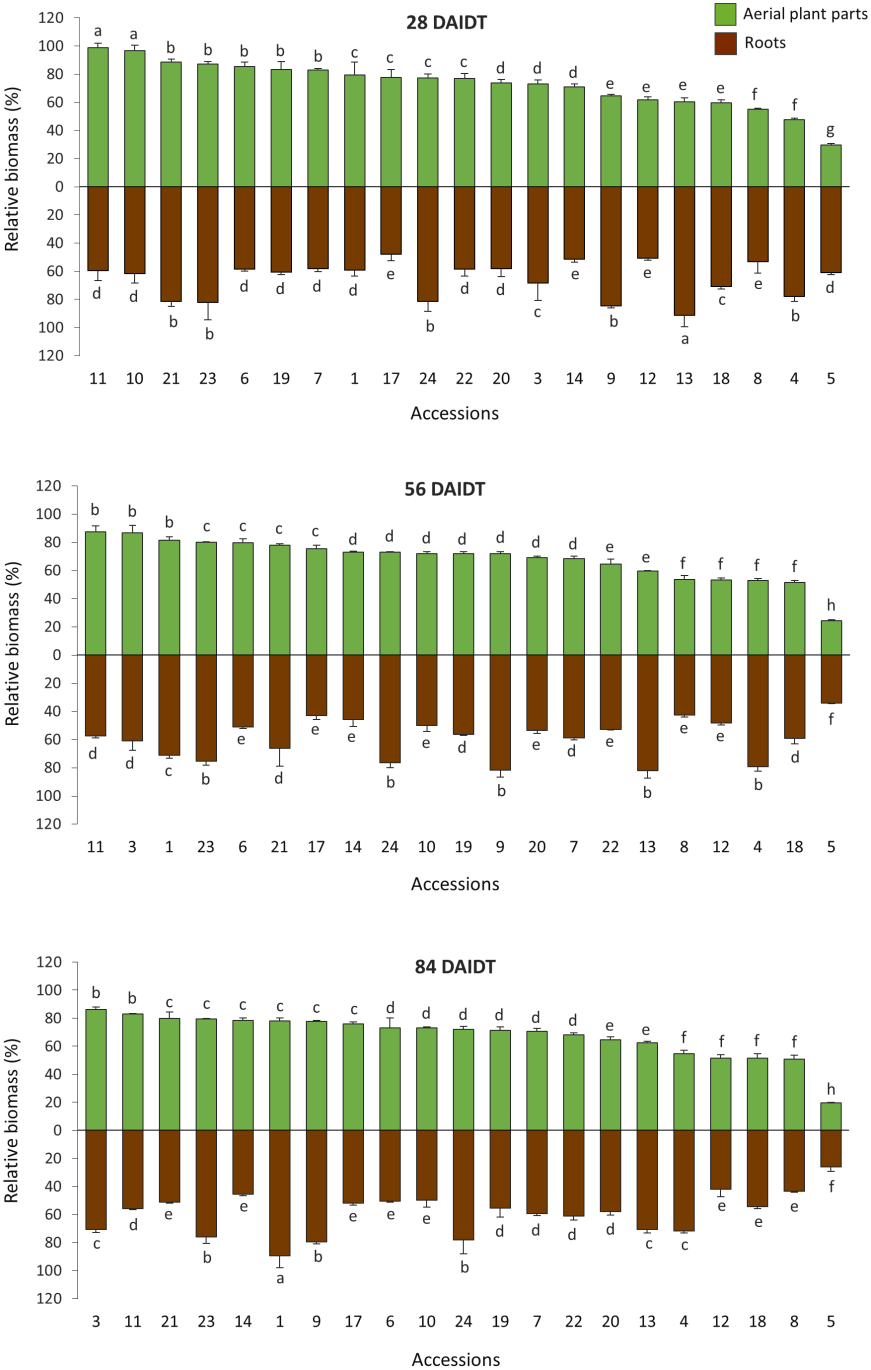
Time-course variation for relative foliage (RFDM) and roots dry matter (RRDM) content in 21 *T. crinita* accessions grown under drought conditions in 2018. Data are expressed as percentage of dry matter (DM) relative to the DM in these plant parts in their respective irrigated control plants, according to the formula (DM_Drought_/DM_Irrigated_) × 100. Foliage dry matter is the sum of DM values for stems, leaves, and panicles. Bars represent means of three biological replicates ± standard deviations. Data for 28, 56, and 84 days after initiation of the drought treatment (DAIDT) are presented, whereas baseline data (0 DAIDT) are not shown because all mean values were ~100% and not statistically different from each other. For each time point, data are presented in decreasing order based on the accessions RFDM levels. Bars not sharing a common letter are significantly different at p<0.05, DGC test.

The foliage/root ratio of *T. crinita* plants under drought stress, relative to this ratio in irrigated controls, referred to as ‘relative foliage/root ratio’ (RFRR), reflects whether the plant allocates more resources to the roots (RFRR > 100%) or the foliage (RFRR < 100%) under drought conditions, in comparison to controls. As depicted in **Figure 2A**, in 2018, highly variable and divergent responses were observed early in the drought treatment (28 DAIDT) among the accessions, with mean RFRR values varying from ~49% (indicating greater biomass allocation in the roots) to 168% (greater allocation in the foliage). In general, after this initial response, most of the accessions leveled-off or slightly inverted their trends as the drought stress continued but, in most cases, their RFRR values at 84 DAIDT did not vary much from the initial response at 28 DAIDT (i.e., the accessions that initially allocated more biomass to the roots finished the drought treatment with less than 100% RFRR, and the opposite occurred with accessions that initially allocated more biomass to the foliage). A group of six accessions (14, 6, 10, 11, 17, and 21) presented statistically greater RFRR values than the rest of the accessions at 84 DAIDT, and three of them (11, 21, 14) also ranked among the plant materials with greatest relative foliage biomass (**Figure 3**). **Figure 2B** presents RFRR data for year 2019 in the six selected contrasting accession. Accession 5 consistently allocated the greatest proportion of biomass to its roots, as indicated by the significantly lowest RFRR values found in this material for both years and all the time-points. On the drought-tolerant extreme, in 2019 accession 1 revealed the greatest mean RFRR value at the end of the experiment, but without reaching statistical differentiation with the rest of the accessions.

### 3.3 Photoassimilates partitioning

**Figure 3** depicts time-course variation for relative biomass content in the foliage and roots of all the accessions grown under drought conditions, and expressed as percentage of the DM content in the foliage (RFDM) and roots (RRDM) of control plants. As expected, at 0 DAIDT, all the accessions had RFDM and RRDM values close to 100%, being not statistically different from each other (data not presented). After the drought stress was imposed, significant variation among the accessions was found for RFDM and RRDM at all the time points analyzed, with mean values for both traits generally decreasing in a genotype-dependent fashion as the drought conditions progressed. Thus, after 56 and 84 DAIDT, mean RFDM, which includes all the above-ground organs (stems, leaves, and panicles), varied from 19.6% to 87.4% across the accessions, whereas RRDM ranged from 26.2% to 89.7%. These data indicate broad genetic variation in the *T. crinita* germplasm with regard to the accessions ability to maintain root and foliage growth under drought stress.

For RFDM, accession 5 was the most affected plant material, and this was evidenced early during the drought stress (at 28 DAIDT), followed by accessions 8, 18, 12, and 4. Conversely, accessions 11, 3, and 1 were the least affected by drought at 56 DAIDT, with accessions 3 and 11 being also the least affected at 84 DAIDT. While most of the accessions either decreased or maintained statistically unaffected their mean RFDM values as the drought progressed, two accessions, namely accs. 3 and 9, significantly increased their RFDM values at 56 and 84 DAIDT compared to 28 DAIDT.

Variation for RRDM partially coincided with RFDM, as shown in **Figure 3**, and as indicated by the low to moderate -yet significant- correlation coefficient (r) values obtained between these two variables at 56 DAIDT (r=0.30, p=0.0178) and 84 DAIDT (r=0.55, p<0.0001), suggesting some level of independence between the two traits. Under prolonged drought conditions (56-84 DAIDT), mean RRDM was significantly lowest in accession 5; and greatest in accession 1 (at 84 DAIDT) and accessions 9, 13, 4, 24, and 23 (at 56 DAIDT). The majority of the accessions revealed significant decreases in –or maintained statistically unaffected- their mean RRDM values as the drought treatment progressed, with the exception of accessions 1 and 3, which increased their RRDM values at 84 DAIDT.

Results for the six contrasting accessions reevaluated in 2019 fully agreed with data from the previous season, clearly separating the drought-tolerant from the sensitive accessions (**Supplementary Figure S3**). Briefly, broad and significant variation (p<0.001) was found among the accessions for both RFDM and RRDM, with the range of variation increasing as the drought treatment progressed For the last two sampling times, all the tolerant accessions (accs. 1, 3, and 9) had significantly greater RFDM and RRDM values than the sensitive accessions (accs. 5, 18, and 22). At these time points, accessions 3 and 1 were the least affected, and accession 5 the most affected, for both variables.

**Figure 4:**
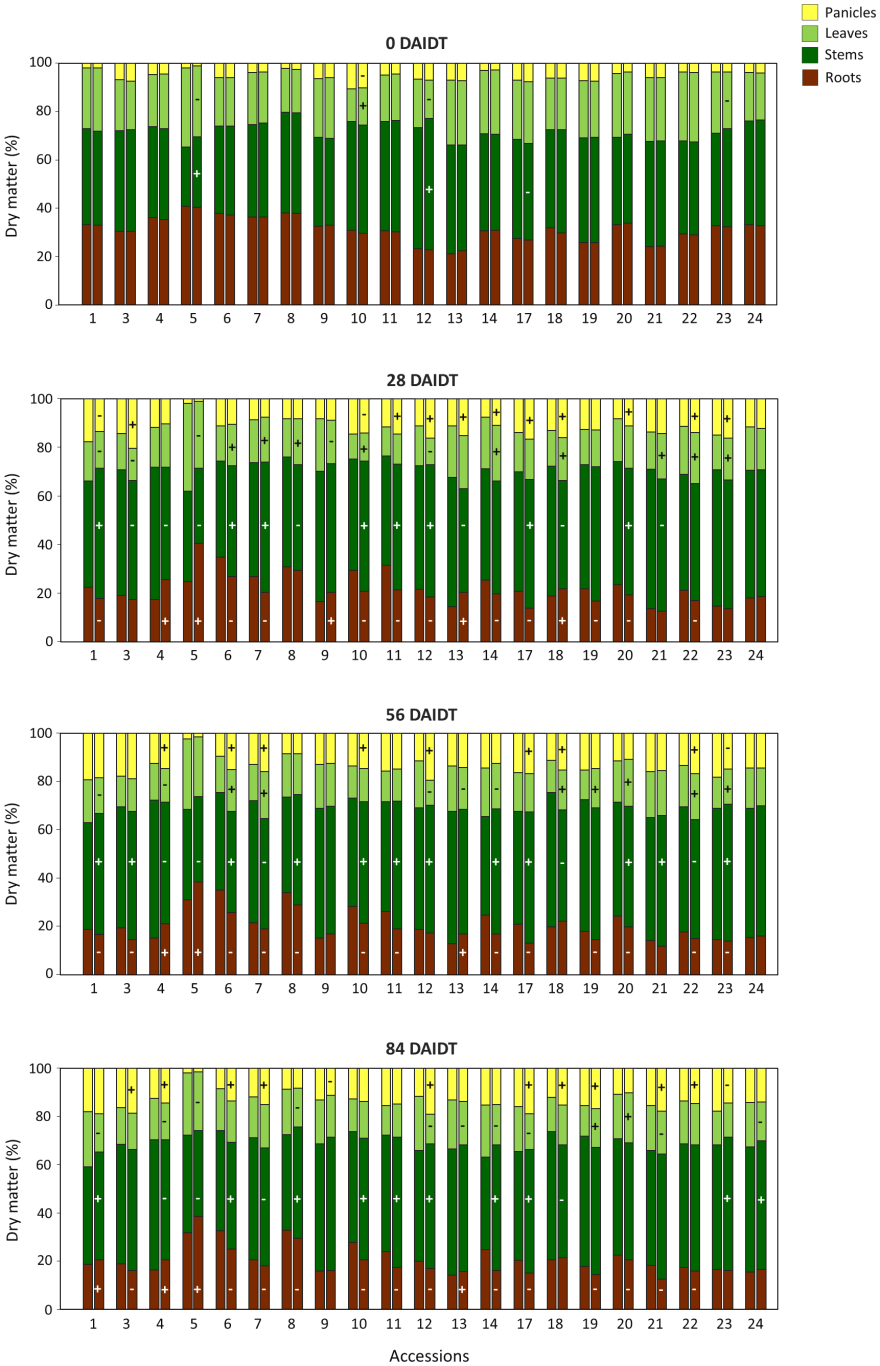
Percentage of the total dry matter (DM) per plant partitioned to different organs before the drought treatment (0 DAIDT), and 28, 56, and 84 days after initiation of the drought treatment (DAIDT) for 21 *T. crinita* accessions grown under irrigated (left bar) and drought conditions (right bar) in 2018. Plus (+) and minus (-) symbols in the drought treatment bar indicate significant increase and decrease in DM partitioning to a particular organ, respectively, relative to the irrigated (control) plants, at p<0.05, DGC test.

Further analysis investigated DM partitioning to individual organs of the plant under irrigated and drought conditions. ANOVA for the percentage of the total DM allocated in roots, stems, leaves, and panicles revealed significant effects for the accession, the drought/irrigation treatment, the year of cultivation, the sampling time, and many of their interactions (**Supplementary Tables S1, S2**). **Figure 4** depicts the variation found among the accessions for photoassimilates partitioning to these organs under irrigated and drought conditions. Nine accessions (accs. 6, 7, 10, 11, 14, 17, 19, 20, 22) consistently revealed significant decreases in DM partitioning to the roots for all the time points considered after the drought stress was imposed (i.e., 28, 56, and 84 DAIDT). Conversely, three other accessions (accs. 4, 5, and 13) consistently exhibited significantly greater biomass partitioning to the roots during the entire drought treatment. For the remaining eight accessions, no consistent variation was observed in photoassimilates partitioning to the roots. Overall, under drought conditions, biomass partitioning to the roots was greatest in accession 5, presenting 25-32% of the total plant DM allocated in its roots, whereas accession 21 had the lowest partitioning to this organ, accounting for 14-18% of the total DM.

Partitioning to individual above-ground organs of the plant varied significantly among the accessions, and between the drought and irrigated treatments within each accession (**Figure 4**). In some accessions, drought stress was associated with reduced biomass partitioning to the leaves while increasing partitioning to the stems and panicles. This was more evident at the end of the drought treatment (84 DAIDT), when ten accessions exhibited significantly greater DM allocation in the stems and panicles, and nine accessions had reduced DM in leaves. Some exceptions to this trend were accessions 4, 5, and 18, which exhibited significantly lower partitioning to the stems consistently during all the drought-stress period (i.e., at 28, 56, and 84 DAIDT), and accession 7, showing the same performance for the last two time points. For the rest of the accessions, no consistent variations in DM allocation to specific above-ground organs were observed during the drought treatment.

Considering the six selected accessions, no direct association was found between the patterns of photoassimilates partitioning to different plant organs under drought conditions with the two sub-classes of accessions (i.e., tolerant *vs.* sensitive). For example, within the drought-sensitive group, accession 5 increased partitioning to the roots and decreased DM in the leaves, whereas accession 22 decreased DM allocation in the roots while increasing its content in panicles, and their performances were consistent throughout the drought treatment (**Figure 4**). In the ‘tolerant’ subgroup, accession 1 consistently revealed reduced DM in leaves while it was increased in stems, whereas no clear trend was observed for accessions 3 and 9. In 2019, data for biomass partitioning to different plant parts generally agreed with results from the previous year (**Supplementary Figure S4**). Again, no clear association was found between DM partitioning patterns under drought conditions with the two subgroups of accessions. Coincidently with results from 2018, under drought stress, accession 5 consistently showed increased DM allocation in the roots and decreased DM in the stems, relative to its control, and presented the greatest percentage of DM allocation in the roots for all the accessions,

### 3.4 Leaf area

**Figure 5:**
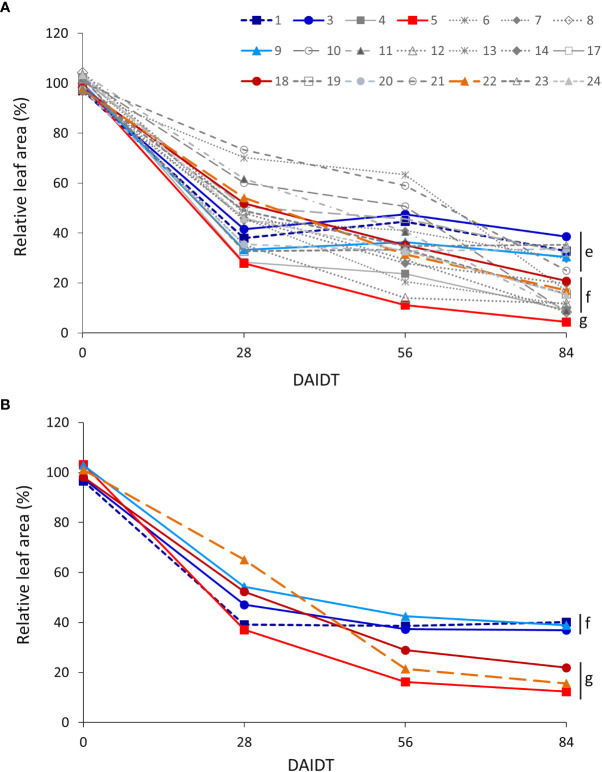
Time-course variation for relative leaf area (RLA) per plant for 21 *T. crinita* accessions grown under drought conditions in 2018 **(A)**. The experiment was partially replicated with six contrasting accessions in 2019 **(B)**. RLA is expressed as percentage of the total leaf area in the respective irrigated controls, as calculated by the formula: RLA = (LA_Drought_/LA_Irrigated_) × 100. Data points with different letters on the right indicate significantly different mean values at the end of the drought treatment at p<0.05 (DGC test), according to the means comparison analyses for all the accessions, time-points, and years presented in **Supplementary Table S4**. DAIDT. Days after initiation of the drought treatment.

ANOVA for relative total leaf area (RLA) revealed significant effects for accession and sampling time (but not for year), and for all the interactions among accession, sampling time, and year (**Table 2**). **Figure 5** depicts time-course variation for RLA for both years of experiments. In 2018, the drought treatment significantly reduced RLA in all of the accessions, as evidenced by the sudden and significant drop in mean RLA values from 28 DAIDT to the end of the experiment (**Figure 5A**). For most of the accessions, such decay in RLA further progressed during the drought treatment, reaching minimum values at 84 DAIDT. However, for a few accessions (namely, accs. 1, 3, 7, 9, 17, and 20), mean RLA values rapidly dropped at the beginning of the drought stress (28 DAIDT), along with most other accessions, but then they remained statistically invariable throughout the rest of the drought treatment. As result, these accessions had the greatest RLA at the end of the experiment (84 DAIDT), together with accession 23. Similar results were obtained in 2019 for the subset of six selected accessions, with the ‘tolerant’ accessions 1, 3, and 9 exhibiting significantly greater RLA than the ‘sensitive’ accessions 5, 18, and 22 at the end of the drought treatment (**Figure 5B**).

In order to integrate and compare the relative leaf area of the accessions during the entire drought treatment, the RLA data for the four time points analyzed were plotted in a graph (the one shown in **Figure 5**) and the ‘area under the curve’ (AUC) was calculated for each accession. Comparisons of the resulting mean AUCs revealed significant variation among the accessions (p<0.05), with accessions 21 and 6 showing the greatest AUC, and accessions 5, 12, and 4 the lowest AUC (**Supplementary Figure S5A**). In 2019, accession 9 had the greatest mean AUC and accession 5 the lowest, whereas the rest of the accessions (accs. 1, 3, 18, 22) were intermediate and statistically comparable (**Supplementary Figure S5B**).

### 3.5 Leaf chlorophyll index

**Figure 6:**
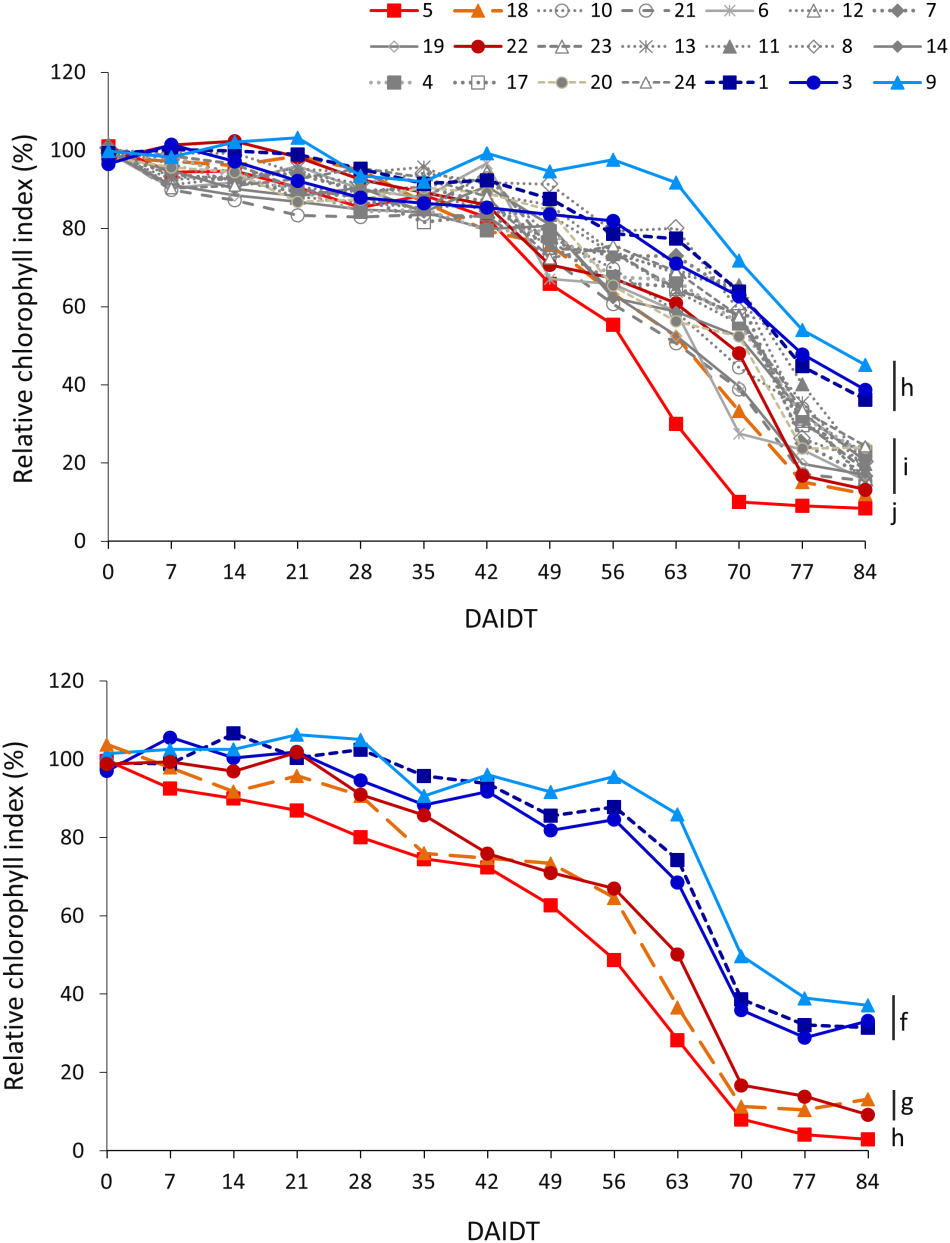
Time-course variation for relative chlorophyll index (RCI) (SPAD units) for 21 *T. crinita* accessions grown under drought conditions in 2018 **(A)**. The experiment was partially replicated with six contrasting accessions in 2019 **(B)**. RCI is expressed as percentage of the chlorophyll index in the respective irrigated controls, as calculated by the formula: RCI = (CI_Drought_/CI_Irrigated_) × 100. Data points with different letters on the right indicate significantly different mean values at the end of the drought treatment at p<0.05 (DGC test), according to the means comparison analyses for all the accessions, time-points, and years presented in **Supplementary Table S5**. DAIDT. Days after initiation of the drought treatment.

Mean leaf chlorophyll index (CI) in *T. crinita* plants grown under drought stress, relative to the CI of irrigated controls, referred to as ‘relative CI’ (RCI), varied significantly among the accessions and among time-points during the drought treatment (**Figure 6**). In 2018, little variation was observed among the accessions during the first 42 DAIDT, with most of the genotypes presenting CI values statistically comparable to their irrigated controls (data not presented), but as the drought stress progressed, the CI values of all but one of the accessions dropped, at varying rates (**Figure 6A**). A subgroup of nine accessions (accs. 5, 6, 7, 8, 18, 19, 20, 21 and 22) revealed the most rapid decay in mean RCI values, exhibiting these accessions the lowest RCI levels at the end of the drought treatment. The latter subgroup included accessions 5, 18, and 22, considered as drought-sensitive. These three materials, and accession 5 in particular, exhibited the lowest mean RCI values of all at 84 DAIDT. Conversely, accessions 9, 1, and 3 showed more gradual decays in RCI levels during the drought treatment, ending these accessions with the greatest RCI values of all at 84 DAIDT. Considering the entire germplasm collection, accession 9 was the least affected of all, exhibiting RCI values statistically comparable to the irrigated control until 63 DAIDT (data not shown), and then gradually decreased to end up with the greatest mean RCI value at 84 DAIDT, along with accessions 1 and 3. The rest of the accessions exhibited intermediate performances between these two sets of contrasting materials. **Supplementary Figure S6A** presents mean RCI values integrated throughout the drought treatment, expressed as AUC, for all the accessions in 2018. Such integrated analysis revealed eight significantly different groups, with accessions 5 and 9 being the plant materials with lowest and greatest mean AUC, respectively, in full agreement with their time-course performances shown in **Figure 5A**.

In the 2019 experiment, RCI data for the six selected accessions varied following a similar pattern as in 2018, with the drought-tolerant accessions (1, 3, and 9) presenting a delayed and more gradual decay of their mean RCI values, as compared to the sensitive accessions (5, 18 y 22), resulting the formers in significantly greater RCI values at the end of the experiment (**Figure 6B**). Consequently, the AUC for this variable was also significantly greater in all the tolerant accessions as compared to the sensitive materials, with accessions 5 and 9 representing the most contrasting extremes (**Supplementary Figure S6B**).

### 3.6 Photochemical efficiency of photosystem II (Fv/Fm)

**Figure 7:**
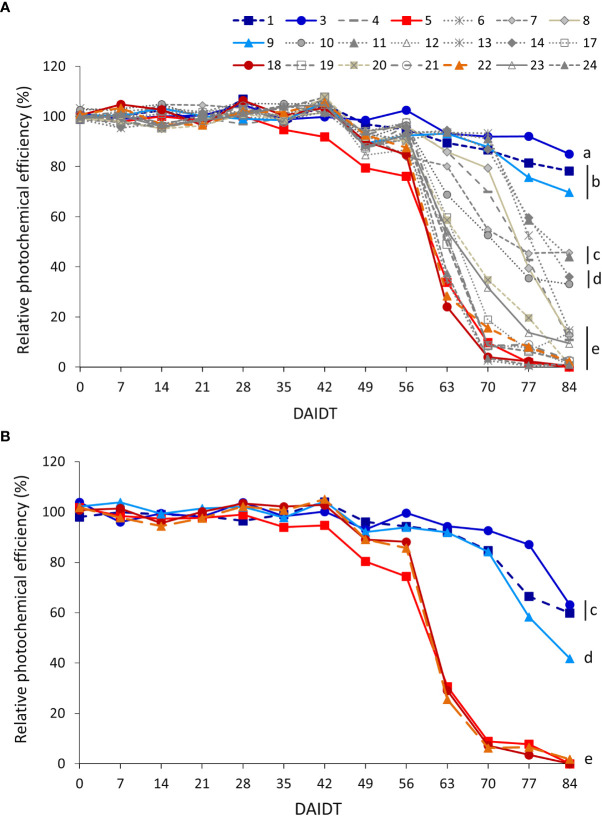
Time-course variation for relative intrinsic photochemical efficiency (RPE) of photosystem II, estimated by the ratio ‘variable chlorophyll fluorescence/maximum chlorophyll fluorescence’ (Fv/Fm), for 21 *T. crinita* accessions grown under drought conditions in 2018 **(A)**. The experiment was partially replicated with six contrasting accessions in 2019 **(B)**. RPE is expressed as percentage of the intrinsic photochemical efficiency (IPE) in the respective irrigated controls, as calculated by the formula: RPE = (PE_Drought_/IPE_Irrigated_) × 100. Data points with different letters on the right indicate significantly different mean values at the end of the drought treatment at p<0.05 (DGC test), according to the means comparison analyses for all the accessions, time-points, and years presented in **Supplementary Table S6**. DAIDT. Days after initiation of the drought treatment.

The photochemical efficiency (PE) of photosystem II (estimated by Fv/Fm ratio) in leaves of *T. crinita* plants grown under drought stress, relative to the PE of irrigated controls, referred to as ‘relative PE’ (RPE), was significantly influenced by the accession, sampling time, year of cultivation, and most of their interactions (**Table 2**). **Figure 7** depicts the time-course variation for RPE in all the accessions during the drought treatment. In 2018, little variation was observed among the accessions during the first 35 DAIDT, with most of the accessions presenting PE values statistically comparable to their irrigated controls (i.e., mean RPE values were close to 100%), but as the drought stress progressed, RPE values of all the accessions began to decrease, at varying rates, with the most abrupt decay observed between 56 and 63 DAIDT for the accessions exhibiting the fastest decline in RPE levels (**Figure 7A**). From 56 DAIDT to the end of the experiment, the drought-stress response of the accessions became evidently and significantly different, varying from genotypes that maintained high levels of RPE (>70%) until the end of the drought treatment (e.g., accs. 3, 1, and 9) to genotypes that rapidly decreased their mean RPE, reaching minimum levels at 84 DAIDT close to 0% (e.g., accs. 5, 6, 18, 19, 21, 22, and 24). **Supplementary Figure S7A** presents integrated mean RPE values throughout the drought treatment and expressed as AUC for all the accessions. As depicted, accession 5 had significantly lowest mean AUC, whereas a group of six accessions (accs. 3, 1, 9, 11, 14, and 13) had the greatest AUC values.

In 2019, RPE data for the six selected accessions varied following a similar pattern as in 2018, with the drought-tolerant accessions (1, 3, and 9) presenting a delayed and more gradual decay of their mean RPE values, as compared to the sensitive accessions (5, 18, and 22), which exhibited an abrupt decay in RPE between 56 and 70 DAIDT, to reach minimums of less than 2% at the end of the experiment (**Figure 7B**). Coincidently with these results, the AUC for RPE in all the tolerant accessions was significantly greater than in the sensitive accessions (**Supplementary Figure S7B**). Altogether, the RPE data from both years suggest accessions 3, 1, and 9 as the most drought-tolerant genotypes, and accession 5 as the most sensitive one.

### 3.7 Stomatal conductance

**Figure 8:**
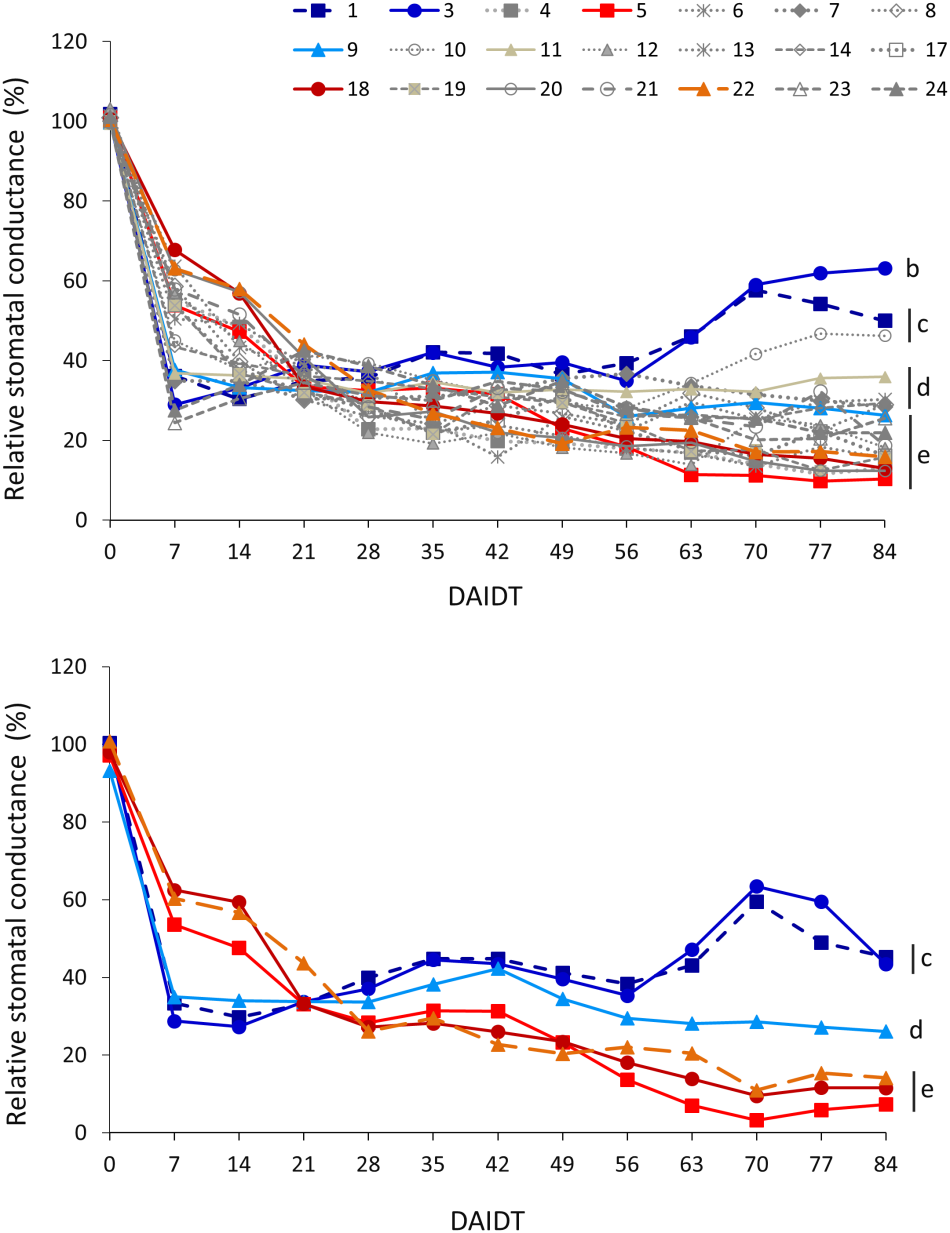
Time-course variation for relative stomatal conductance (Rg_s_) (mmol m^2^ s^-1^) for 21 *T. crinita* accessions grown under drought conditions in 2018 **(A)**. The experiment was partially replicated with six contrasting accessions in 2019 **(B)**. Rg_s_ is expressed as percentage of the stomatal conductance (g_s_) in the respective irrigated controls, as calculated by the formula: Rg_s_ = (g_sDrought_/g_sIrrigated_) × 100. Data points with different letters on the right indicate significantly different mean values at the end of the drought treatment at p<0.05 (DGC test), according to the means comparison analyses for all the accessions, time-points, and years presented in **Supplementary Table S7**. DAIDT. Days after initiation of the drought treatment.

Mean stomatal conductance (g_s_) in *T. crinita* plants grown under drought stress, relative to the g_s_ of the irrigated control plants, referred to as ‘relative g_s_’ (Rg_s_), was significantly influenced by the accession, sampling time, year of cultivation, and most of their interactions (**Table 2**). In 2018, the effect of the drought stress on Rg_s_ was evidenced very early in the experiment, showing significant decreases -to varying extents depending on the genotype- for all 21 accessions in the first week of treatment (**Figure 8A**). In this short period, mean Rg_s_ values dropped from ~100%, before initiation of the drought treatment, to 24% in acc. 23, and up to 68% in acc. 22. In decreasing order, accessions 23, 24, 3, 1, 7, 11, 14, 9, and 10 were the most affected at this time point, exhibiting absolute stomatal conductance values in the range of 10.4-15.2 mmol m^2^ s^-1^ (data not presented). However, after this sudden drop in mean Rg_s_ values, these accessions stabilized and –in some cases- even increased their Rg_s_ throughout the rest of the drought treatment, exhibiting at the end of the experiment significantly greater stomatal conductance than most of the other accessions. In contrast, the accessions that had a mild decrease in Rg_s_ at the beginning of the drought stress (7 DAIDT) (e.g., accs. 5, 18, 22, 20, and 24, among others), continued their decreasing trend throughout the rest of the experiment, reaching the lowest Rg_s_ values of all the accessions, with means in the range of 12.3-16.8% (6.4-9.2 mmol m^2^ s^-1^, in absolute g_s_ values), at the end of the drought period. **Supplementary Figure S8A** presents mean Rg_s_ values integrated throughout the drought treatment and expressed as AUC for all the accessions, showing accessions 3 and 1 with significantly greatest AUC, whereas eight accessions (accs. 4, 12, 13, 19, 17, 23, 5, and 20) comprised the lowest AUC group.

In 2019, Rg_s_ data for the six selected accessions varied following a similar pattern as in 2018. Thus, the drought-tolerant accessions 1, 3, and 9 exhibited a sudden and more profound decrease in mean Rg_s_ than the drought-sensitive genotypes (5, 18, and 22) at 7 DAIDT but, as observed in the previous year, the former group stabilized and –for accessions 1 and 3- increased their mean Rg_s_ levels, ending up with significantly greater Rg_s_ at 84 DAIDT (**Figure 8B**). Conversely, the drought-sensitive accessions continued to decrease their Rg_s_ during the rest of the drought treatment, reaching significantly lowest Rg_s_ values at the end of the experiment. Interestingly, accession 9 behaved as an intermediate material, being its mean Rg_s_ at the end of the drought period statistically lower than the Rg_s_ of accessions 1 and 3, and greater than that of the sensitive genotypes. Coincidently with these results, the AUC for Rgs in 2019 was significantly greatest in accessions 1 and 3, and lowest in accession 5, with the rest of the accessions being intermediate relative to the formers (**Supplementary Figure S8B**).

### 3.8 Number of panicles per plant

**Figure 9:**
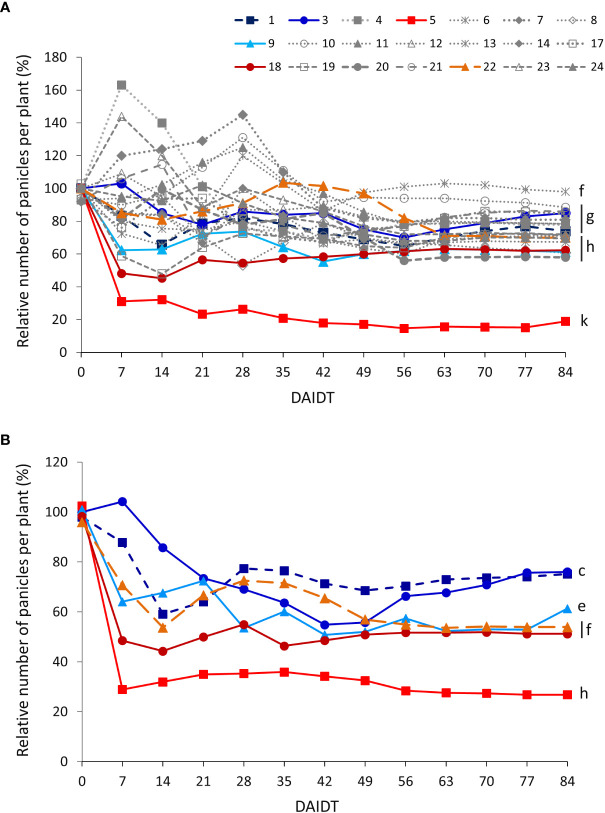
Time-course variation for relative number of panicles per plant (RNPP) for 21 *T. crinita* accessions grown under drought conditions in 2018 **(A)**. The experiment was partially replicated with six contrasting accessions in 2019 **(B)**. RNPP is expressed as percentage of the number of panicles per plant (NPP) in the respective irrigated controls, as calculated by the formula: RNPP = (NPP_Drought_/NPP_Irrigated_) × 100. Data points with different letters on the right indicate significantly different mean values at the end of the drought treatment at p<0.05 (DGC test), according to the means comparison analyses for all the accessions, time-points, and years presented in **Supplementary Table S8**. DAIDT. Days after initiation of the drought treatment.

The number of panicles per plant (NPP) in *T. crinita* accessions grown under drought stress, relative to the NPP in their respective controls, referred to as ‘relative NPP’ (RNPP), was significantly influenced by the accession, the year of cultivation, the sampling time, and their interactions (**Table 2**). In 2018, an early response was observed for some of the accessions, either increasing (accs. 4, 7, 10, and 23) or decreasing their RNPP (e.g., accs. 5, 18, 19, 9, and 6) in the first 28-35 DAIDT (**Figure 9A**). All but one of these accessions stabilized their RNPP values as the drought conditions progressed, to end up with mean RNPP values in the range of 58-98% at 84 DAIDT. Accessions 6 and 10 were the only ones that maintained high RNPP values until the end of the trial, showing no statistical differences with their basal values at 0 DAIDT (i.e., at 84 DAIDT, their RNPP values were ~100%). Accession 5 was the most affected of all, exhibiting an abrupt decay in RNPP early during the drought treatment, which then slowly decreased until the end of the experiment to a final value of 19% RNPP. In 2019, RNPP data generally coincided with results from the previous year, with accession 5 revealing a very similar variation pattern as in 2018, atypical from the rest of the accessions, consistently presenting the lowest mean RNPP values throughout the entire drought treatment (**Figure 8B**). The rest of the accessions followed a similar variation pattern as in the previous year. Although time-course monitoring of this variable did not reveal an early separation of tolerant *versus* sensitive accessions, as observed for other variables, at the end of the drought treatment all the tolerant accessions had significantly greater RNPP values than the sensitive ones, with accessions 3 and 9 being the greater relative number of panicles per plant. Additional comparative analysis of RNPP, expressed as AUC, for both years of data, is presented in **Supplementary Figure S9**.

### 3.9 Relationships among drought response variables

Pairwise correlation coefficient values among all the variables at the end of the drought treatment, for both years, are presented in **Table 4**. In 2018, relative total (RTDM) and foliage biomass (RFDM), two of the most relevant variables reflecting forage yield under drought stress, were strongly correlated with each other (r=0.98, p<0.0001), and both traits were significantly and positively correlated with all the other variables (r=0.34-0.74, p<0.01). This suggest that, under prolonged drought conditions, the accessions with greater forage biomass (relative to irrigated controls) tend to have greater relative levels of total biomass production per plant, root biomass, foliage/root ratio, leaf area, chlorophyll content, stomatal conductance, photosynthetic performance, and inflorescences per plant. In 2019, all the variables were significantly and more-strongly correlated (r=0.55-1.00) than in the previous year, most likely reflecting a sampling bias due to the selection of the six most contrasting accessions used in the analysis. Nonetheless, the majority of the correlations found coincided -in significance and sign- between years, despite the observed differences in the strength of the associations.

**Table 4:** Pairwise Pearson correlation coefficient (r) values among nine morpho-physiological traits for *T. crinita* accessions after 84 days of drought stress, for years 2018 and 2019.

	RTDM	RFDM	RRDM	RFRR	RLA	RCI	RPE	RGs	RNPP
RTDM		0.98***	0.69***	0.34**	0.61***	0.55***	0.51***	0.45**	0.68***
RFDM	1.00***		0.53***	0.52***	0.55***	0.46**	0.47**	0.48**	0.74***
RRDM	0.95***	0.93***		-0.42**	0.51***	0.66***	0.42**	0.25*	0.30*
RFRR	0.75**	0.79**	-0.55*		0.03	-0.18	0.05	0.23	0.54***
RLA	0.77**	0.78**	0.71**	0.69**		0.46**	0.35**	0.17	0.30*
RCI	0.83***	0.83***	0.82***	0.62**	0.87***		0.64***	0.41**	0.16
RPE	0.86***	0.86***	0.78**	0.61**	0.75**	0.71**		0.68***	0.21
RGs	0.89***	0.88***	0.84***	0.61**	0.77**	0.71**	0.93***		0.41**
RNPP	0.99***	0.99***	0.94***	0.78**	0.81***	0.86***	0.84***	0.88***	

The diagonal gray boxes separate data for 21 accessions grown in 2018 (upper half) and six accessions in 2019 (lower half). RTDM, relative total dry matter; RFDM, relative foliage dry matter; RRDM, relative root dry matter; RFRR, relative foliage/root ratio; RLA, relative leaf area; RCI, relative chlorophyll index; RPE, relative photochemical efficiency of photosystem II; RGs, relative stomatal conductance; RNPP, relative number of panicles per plant. *, **, *** indicate significant correlation at p<0.05, p<0.01, and p<0.001, respectively.

### 3.10 Principal component analysis

**Figure 10:**
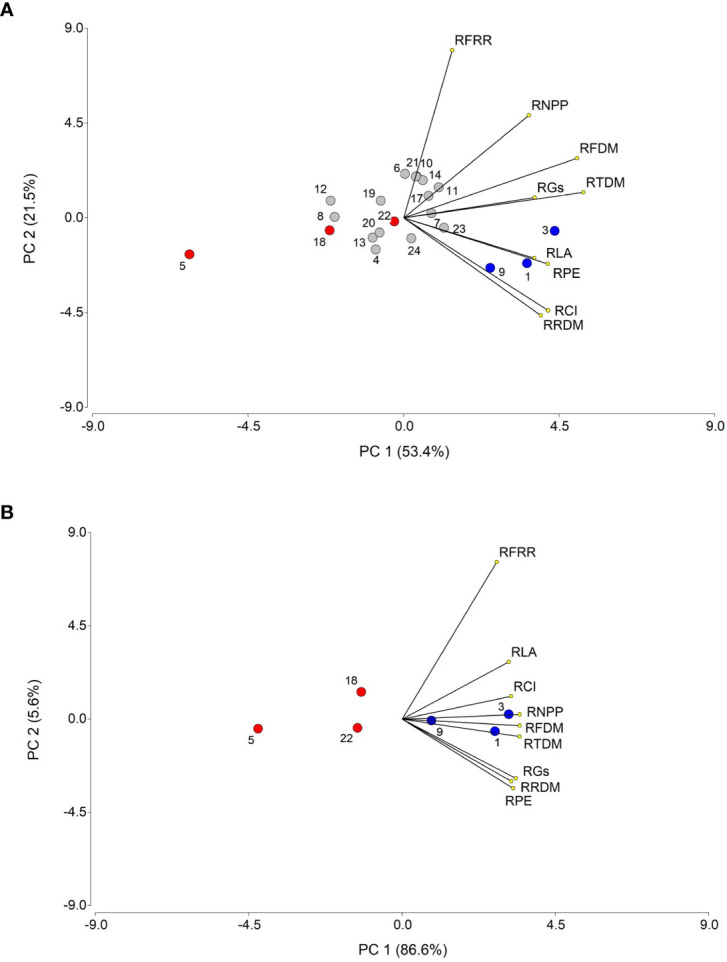
Principal component analysis (PCA) of nine biometric and physiological traits associated with drought tolerance, for 21 *T. crinita* accessions in 2018 **(A)** and 6 accessions in 2019 **(B)** grown under drought conditions for 84 days. The numbers of the accessions refer to the plant materials described in **Table 1**, indicating with blue and red circles the selected drought-tolerant and sensitive accessions, respectively, evaluated over two years, whereas the rest of the accessions are denoted in gray circles. Lines starting from the center point of the bi-plot depict the positive or negative association of the parameters with the two principal components (PC1 and PC2). RTDM, relative total dry matter; RFDM, relative foliage dry matter; RRDM, roots relative dry matter; RFRR, relative foliage/root ratio; RLA, relative leaf area; RCI, relative chlorophyll index; RPE, relative photochemical efficiency (RPE) of photosystem II, RGs, relative stomatal conductance; RNPP, relative number of panicles per plant.

Principal component analyses (PCA) with nine variables were conducted, independently, with data from years 2018 and 2019 (**Figure 10**). In the first year, two principal components (PC) explained, jointly, 74.9% of the total variation, with PC1 accounting for 53.4% of the variation. The variables that contributed most to PC1 were, in decreasing order, RTDM, RFDM, RRDM, RFRR, RLA, RCI, RPE, RGs, and RNPP. A group of nine accessions, located in the right half of the bi-plot, were the most representative of these variables, with accessions 3, 1 and 9 showing the strongest association, whereas most of the remaining accessions were, conversely, located in the left half of the bi-plot, with accession 5 being the one with strongest negative association with the variables. In the second year, considering only the selected six most contrasting accessions, the two main components accounted for 92.2% of the total variation, with PC1 explaining 86.6% and PC2 5.6%. In general, the same variables that were associated with PC1 in 2018, where also associated with this component in 2019. The drought tolerant and sensitive accessions were located on the left and right side of the bi-plot, respectively, with accessions 3 and 5 representing the most contrasting plant materials, as found in the previous year.

## 4 Discussion

The present work investigated variation for drought tolerance in a genetically-diverse germplasm collection of 21 *Trichloris crinita* accessions under natural field conditions by means of monitoring nine morpho-physiological traits associated with drought responses in plants during an 84-days drought treatment, using a partially-replicated two-year experiment. To the best of our knowledge, this is the most comprehensive study published to date concerning drought tolerance in this species, with regards to the number of genotypes, variables, and genetic environments (years) analyzed. Thus, although previous studies have generated valuable information, evidencing genetic variation for drought tolerance in this species, they evaluated relatively few germplasm (2-4 accessions or ecotypes) and traits under a single genetic environment (Greco and Cavagnaro, 2003; Quiroga et al., 2013; Marinoni et al., 2020). Considering that *T. crinita* is a native species of arid and semi-arid regions, covering extensive geographical areas, and promoted for range grazing and revegetation of degraded lands, a relevant aspect of this study is the fact that the experiments were carried out under field conditions, thereby facilitating extrapolation of the results to the species natural environment, whereas previous studies were conducted under controlled conditions, in pots (Greco and Cavagnaro, 2003; Quiroga et al., 2013) or hydroponics in a growth chamber (Marinoni et al., 2020).

Previous evaluations of *T. crinita* germplasm for drought tolerance compared ecotypes or accessions collected from geographical sites varying in water availability [with ranges for mean annual precipitation in the collection sites of 104-324 mm (Greco and Cavagnaro, 2003), 326-625 mm (Quiroga et al., 2013), and 179-1142 mm (Marinoni et al., 2020)], generally finding positive associations between the level of aridity at the collection site and drought tolerance, as estimated by different plant growth and physiological parameters. For instance, Greco and Cavagnaro (2003) found that the accession from the site with greatest aridity was less affected by drought –as compared to irrigated control plants- than the other two accessions originated from less arid regions, reporting greater relative levels of total DM, shoot DM (calculated as the sum of DM in leaves, culms, and sheaths), and total leaf area associated with drought tolerance. Similarly, Marinoni et al. (2020) found that, under drought conditions, ecotypes from the driest collection sites had greater shoot and root biomass yield and greater number of tillers per plant (relative to irrigated controls) than ecotypes from less arid regions. Also, Quiroga et al. (2013) evaluated two ecotypes from different environments, finding that the one from the most arid region was less affected by drought than the one from humid origin, and the tolerant phenotype was associated with a slower extraction of water from the soil, lower leaf senescence rate, and greater leaf expansion rate.

In the present work, which included accessions derived from geographical sites varying in mean annual precipitation from 104 to 519 mm and mean annual temperature from 14.4 to 20.4 °C (**Table 1**), we found no significant correlations between aridity levels in the collection sites [estimated by the aridity indices of De Martonne (1926) and Zomer et al. (2022), and mean annual precipitation data] and the vast majority of the variables analyzed, for the last two sampling times in both years (**Supplementary Table S9**). Only RNPP, RPE, and RGs had significant negative correlations with some of the aridity estimates, but most of these associations were rather weak and marginally significant (r= -0.25 to -052, p=0.026-0.049), and they were inconsistent across years and sampling times. These results suggest no generalized adaptive advantage for drought tolerance associated with the level of aridity in the native environments of the accessions. Furthermore, the two most contrasting materials, accessions 3 (most-tolerant) and 5 (most-sensitive), derived from environments with similar aridity levels, as indicated by comparisons of their mean annual precipitations (327 *vs.* 247 mm) and aridity indices by De Martonne (12.2 *vs.* 9.4) and Zomer et al. (0.15 *vs.* 0.14); whereas collection sites with relatively low (annual precipitation > 400 mm) and high aridity (annual precipitation < 220 mm) both included accessions varying broadly in their drought tolerance responses (**Table 1**). For example, accessions 1 (tolerant) and 22 (sensitive) derived from high-aridity environments, whereas accessions 14 (tolerant, based on total and foliage biomass yield) and 18 (sensitive) derived from La Pampa, the site with greatest annual precipitation (519 mm). Thus, conversely to previous studies suggesting an adaptive drought-tolerance advantage associated with the level of aridity in the accessions natural habitats (Greco and Cavagnaro, 2003; Quiroga et al., 2013; Marinoni et al., 2020), our results rather suggest that different genotypes varying in drought stress tolerance coexist in *T. crinita* natural populations derived from a particular location or environment, regardless of water availability, at least for the range of aridity conditions sampled in the present work. In full agreement with this hypothesis, we previously found that molecular marker (AFLP)-based genetic clustering of these same *T. crinita* accessions was not associated with geographical origin or habitat conditions, and suggested that *T. crinita* natural populations were genetically heterogeneous (Cavagnaro et al., 2006). Further support for genetic heterogeneity in *T. crinita* natural populations comes from studies -using this same germplasm collection- reporting lack of association between geographical origin or aridity levels in the accessions collection sites and various morphometric and quantitative agronomic traits, including forage productivity (Cavagnaro et al., 2006) and nutritional quality (Dominguez et al., 2022), as well as karyotype and cytogenetic characterizations (Kozub et al., 2019). Presumably, differences in the number and nature of the plant materials analyzed (2-4 ecotypes or populations *vs.* 21 accessions derived from single-plant descendants), and the range of aridity in the original habitats (e.g., range for mean annual precipitation was 179-1142 mm in Marinoni et al. (2020) *vs.* 104-519 mm in this work) may partially account for these discrepancies between previous works and the present study. It should be noted that the fact that the three aridity estimates used in our correlation analyses [i.e., the mean annual precipitation, and indices of De Martonne (1926) and Zomer et al. (2022)] yielded comparable results, including those from analysis using the mean annual precipitation (as used in previous studies), suggesting that these discrepancies are not due to differences in the choice of aridity estimators across studies.

Drought stress can strongly influence many plant growth, physiological, and biochemical parameters. In the present study, under prolonged drought stress (84 DAIDT), nearly all the accessions significantly reduced total-, foliage-, and root DM content, as well as total leaf area, chlorophyll content, photochemical efficiency, stomatal conductance, and number of panicles per plant, in comparison to their respective irrigated controls (**Figures 3**, **5**-**9**). The very few exceptions were accessions 1 (for root DM), 3 (for photochemical efficiency), and 6 (for number of panicles per plant), which had levels of these variables that were statistically comparable to their controls (**Figures 3**, **7**, **9**). These data agree with our previous study reporting significantly reduced total-, aerial-, and root DM contents, and total leaf area in three *T. crinita* accessions grown under drought stress, as compared to their irrigated controls (Greco and Cavagnaro, 2003). Conversely, foliage/root ratio was the only trait in the present study for which divergently different responses were observed at the end of the drought stress, with some accessions exhibiting significant increases while others showed reduced or unaffected values relative to their controls (**Figure 2**). Interestingly, Greco and Cavagnaro (2003) found that shoot/root ratios did not vary significantly between drought-stressed and irrigated plants for the three accessions tested, whereas Marinoni et al. (2020) reported that, under drought, shoot/root ratios decreased in comparison to irrigated controls in the four *T. crinita* ecotypes analyzed by them. Presumably, differences in the genotypes and number of plant materials used across these studies, as well as methodological differences (e.g., experiments conducted under field *vs.* pots conditions) may explain, at least partially, the different responses observed for this trait under drought stress.

Under drought stress, the productivity of a plant depends on some essential processes, such as the temporal distribution of biomass and the partitioning of photoassimilates (Anjum et al., 2017). The limited availability of assimilates, attributable to arrested photosynthesis and impaired partitioning of assimilates, determines the plant growth response under drought stress. The reorganization of assimilates partitioning under these conditions is generally accompanied by alterations in the expression of genes involved in the metabolism and transport of carbohydrates, and differs between drought-tolerant and sensitive genotypes (Aliche et al., 2020). For instance, some plants modify their assimilate distribution as a stress adaptation strategy and accumulate more soluble sugars in the leaf and root cells to concentrate the cytosol for osmotic adjustment, and reduce transport to the reproductive organs. This alteration in the partition of assimilates becomes inevitable for some plants during the limitation of water in the rhizosphere (reviewed by Chaves et al., 2002; Valliyodan and Nguyen, 2006). In contrast, the reduction in starch content in leaves and roots with decreased water supply is due to the conversion of starch to simple sugars for osmoregulation and the increased relationship between respiration and photosynthesis (Galmés et al., 2007). Plants often reallocate assimilates from shoot growth to root growth under drought conditions, increasing root spread into deeper soil layers (Rich and Watt, 2013). Conversely, other studies have reported decreased root growth in plants under drought stress (Tahere et al., 2000; Cui et al., 2008). Altogether, these studies suggest that the root growth response to drought stress depends on the genotype, the intensity and duration of drought stress, and the rate of stress development.

This work compared drought tolerance among the accessions by means of estimating their performances for nine morpho-physiological traits under drought conditions relative to their own irrigated controls, thereby expressing each variable as relative value (%). In crop breeding programs, one of the most commonly used criteria for selecting drought tolerant genotypes, is the use of indices that estimate yield lost under drought in comparison to normal (non-stressed) conditions (Mitra, 2001). Herein, the variables RTDM and RFDM estimate the loss of yield in total and foliage biomass due to drought, as compared to irrigated controls. Based on these variables, after prolonged drought conditions (84 DAIDT), accession 3 was the most tolerant material, consistently for both years, reducing its total plant biomass only 10-17% and its foliage biomass -a direct estimate of the total forageable biomass- 8-14% (**Table 3**, **Figure 3**, **Supplementary Figure S3**). Besides accession 3, other highly-productive accessions at 84 DAIDT were 1, 9, 11, 14, 17, 21, 23, and 24, which exhibited reductions in total and foliage biomass of 16-37% and 17-38% (data for both years), respectively. As comparison, accession 5, which represented the most drought-sensitive extreme, had a reduction of 78-81% and 80-83% of its total and foliage biomass, respectively, for the same time-frame and conditions. However, we observed that some members of the former group of high-biomass yielding materials –namely accessions 11, 14, 17, 21, 23, and 24- exhibited some dead plants and/or plants with a large proportion of dead tissue at the end of the drought treatment, also evidenced by their very low values for variables that reflect photosynthetic activity, such as RCI, RPE, and RGs (**Figures 6**-**8**, **Supplementary Tables 5-7**). Thus, based on these data, we took into consideration other variables, besides relative biomass yield, for the selection of the most tolerant plant materials. As result, accessions 1, 3, and 9 were selected as most tolerant because they consistently presented high relative biomass (estimated by the variables RTDM, RFDM, and RRDM), leaf area (RLA), and non-senescent functional leaves with photosynthetic activity (RCI, RPE, RGs) at the end of the drought treatment.

The most productive and tolerant accessions tended to have significantly greater mean values than the least productive and sensitive materials, for all the traits analyzed, at the end of the drought stress. This tendency was also reflected by the significant and positive correlations found between RTDM and RFDM with the rest of the variables in 2018 (r=0.34-0.74) and 2019 (r=0.75-0.99) (**Table 4**), and by the strong association between RTDM and RFDM with the most explanatory component in the PCAs of both years, along with most of the other variables (**Figure 10**). This suggest that under prolonged drought conditions, the tolerant accessions were able to sustain high relative biomass production in the aerial plant parts (predominantly in stems and panicles; **Figure 4**) and –to a lesser extent- in roots, resulting in greater relative foliage/root ratios and leaf area, exhibiting also greater chlorophyll content, stomatal conductance, and photochemical efficiencies than the drought-sensitive accessions. These results coincide with our previous findings showing that the most drought-tolerant accession had greater relative levels (expressed as a fraction of irrigated controls) of total leaf area, total DM, and shoot DM (equivalent to ‘foliage DM’ in the present study) than the other two –and more sensitive- accessions (Greco and Cavagnaro, 2003). They also agree with those of Marinoni et al. (2020) reporting that, under drought conditions, the two most tolerant ecotypes had greater relative levels of shoot DM, root DM, and tillers per plant than the more sensitive ecotypes.

Under drought conditions, *T. crinita* accessions varied widely with regards to the drought-response variables analyzed, and this may suggest mechanistic differences between the tolerant and sensitive genotypes in their ability to cope with such stress. For instance, the greater reduction in biomass yield components (RDTM, RFDM, RRDM, RLA) revealed in sensitive accessions could be a consequence of a greater reduction in their photosynthesis rate, presumably due to an earlier stomatal closure (lower RGs; **Figure 8**) triggered by higher abscisic acid (ABA) concentrations in response to the drought stress (Popova et al., 2000), and greater damage to the photosynthetic apparatus, as suggested by the earlier decay and much lower final RPE values observed –for these accessions- at the end of the drought stress (**Figure 7**). This drought stress-induced damage is generally accompanied by degradation of chlorophyll pigments, which is also suggested by the faster decay of RCI values in sensitive *vs.* tolerant accessions (**Figure 6**), as well as reduced concentration and enzymatic activity of Rubisco, weakened electron transport and photosynthesis photophosphorylation, and altered levels of relevant metabolites (Seleiman et al., 2021). In comparison, drought tolerant accessions seem to have some sort of protective mechanisms that postpone and/or attenuate the negative effects of drought stress on these physiological parameters, as indicated by the delayed and/or ameliorated decay in most of the drought-response variables analyzed (**Table 1**, **Figures 3**, **5**-**9**). In line with this hypothesis, we previously found that the most drought-tolerant of three *T. crinita* accessions had –at any given time point during the drought treatment- greater leaf water potentials, suggesting a greater efficiency at minimizing the loss of water status in the plant, and the appearance of the first drought symptoms (folded leaves) were significantly delayed (14-28 days), as compared to the more sensitive genotypes (Greco and Cavagnaro, 2003). Additional studies with a few highly-contrasting (tolerant *vs.* sensitive) accessions examining a larger number of physiological parameters and paralleled with global gene-expression analysis (e.g., transcriptome profiling) may be necessary to fully understand the mechanisms underlying drought resistance in this species.

A general belief in plant ecophysiology is the trade-off between the capacity of a genotype to grow when resources are abundant, and its capacity to tolerate resource shortages (Chapin, 1980; Huston, 1994; Bazzaz, 1996). For arid environments, this paradigm predicts a negative association between the potential biomass yield under optimal water availability (i.e., under irrigation) and drought tolerance. Results from our previous work with three *T. crinita* accessions matched the predicted negative association between potential biomass yield and drought tolerance (i.e., the least productive accession under optimal water availability was the most drought-tolerant material, and viceversa) (Greco and Cavagnaro, 2003), thereby supporting the trade-off hypothesis. However, from our present data, using a much larger number of accessions, no performance trade-offs emerged between optimal growth and drought tolerance, as indicated by the absence of significant negative correlations between total and foliage biomass yield under irrigated conditions *versus* the majority of the drought-response variables analyzed [the only exception was RFRR which showed a weak association (r=-0.26 to -0.30) with the former variables in 2018 but not 2019]. In contrast, we found significant positive correlations between potential biomass yield and four major drought-response variables (RTDM, RFDM, RRDM, and RLA), with r values in the range of 0.26-0.61 (data not shown), suggesting that the most productive genotypes under optimal water availability also tend to be more productive under drought stress. In agreement with this line of evidence are two contrasting examples, namely accession 5, the least productive genotype under irrigation and yet the most drought-sensitive one; and accession 9, exhibiting high biomass yield under irrigation and high tolerance to drought. Altogether, our current data does not support the trade-off hypothesis. Instead, they coincide with more recent studies which have explicitly tested, and rejected, this hypothesis in several grass species (Fernandez and Reynolds, 2000; Couso et al., 2010; Jung et al., 2020).

We found broad genetic variation for drought tolerance in this *T. crinita* collection, as indicated by all the drought-response variables analyzed (**Tables 2**, **3**, **Figures 3**-**9**). Overall, accessions 3 and 5 were the most extreme and contrasting genotypes in the entire germplasm collection for the majority of the traits analyzed in both years, with accession 3 being most tolerant for five of the nine traits considered (RTDM, RFDM, RLA, RPE, Rg_s_) and accession 5 being the most sensitive one for eight traits (RTDM, RFDM, RRDM, RLA, RCI, RPE, Rg_s_, and RNPP). More broadly, considering all the traits and years, accessions 1, 3, and 9 were selected as most drought-tolerant, whereas accessions 5, 18, and 22 were considered most sensitive. Considering these two subsets of contrasting accessions, it should be noted that, at the end of the drought treatment, all the tolerant materials had statistically superior (p<0.05) performances than the sensitive accessions for nearly all the variables, consistently for both years of data (**Table 3**, **Figures 3**, **5**-**9**, **Supplementary Tables S4-S8**), and this was also reflected by their evident separation in the PCAs integrating all the variables (**Figure 10**). The only exception was RFRR, for which several tolerant and sensitive accessions overlapped and were statistically comparable at the end of the drought treatment in both years (**Figure 4**). These most-contrasting accessions will be instrumental for investigating the genetic basis underlying drought tolerance in *T. crinita*. For example, tolerant and sensitive genotypes can be intercrossed to produce F_1_ progenies and, by self-pollination of the latter, F_2_ segregating populations that can be used for mapping quantitative trait loci (QTL) and -combined with comparative transcriptome analysis of tolerant *versus* sensitive plants- identifying candidate genes for drought tolerance. Although the genome of *T. crinita* has not been sequenced, the increasingly widespread use of high-throughput NGS technologies, such as genotyping by sequencing (GBS) and RNA-Seq, can accelerate the construction of highly-saturated linkage maps with well-resolved QTLs, as well as transcriptome profiling, facilitating further strategies for candidate gene identification. Such approach has been successfully used in other grasses (Gelli et al., 2017; Kiranmayee et al., 2020; Pendergast et al., 2022).

The drought-tolerant accessions identified in this study are valuable materials for revegetation and range grazing in extremely arid regions that would otherwise be agriculturally unexploited. This becomes particularly relevant in the current context of climate change, predicting increased temperatures (e.g., a 2–4°C increase in mean diurnal temperature is predicted by the end of the century for the central West part of Argentina), changes in precipitation patterns, and increased desertification in some regions (International Panel of Climatic Changes, 2014). The drought-tolerant accessions 1 and 9 may be of particular interest in this context, considering their high nutritive value as forage, as indicated by recent findings showing that these two accessions were among the plant materials with greatest crude protein content in this same germplasm collection (Dominguez et al., 2022).

From a breeding perspective, it is desirable to combine drought tolerance with high forage biomass yield and nutritive value. Given that these *T. crinita* germplasm have already been characterized –finding broad and significant variation- for these traits (Cavagnaro et al., 2006; Dominguez et al., 2022; this work), and that the reproductive system of *T. crinita* was recently elucidated and classified as autogamous and self-compatible (Gutierrez et al., 2016; Kozub et al., 2017), it is now theoretically feasible to combine these –and other- traits of interest by sexually intercrossing these materials. For this purpose, it is worthwhile noticing that our 21 accessions represent individual genotypes, as they are single-plant descendants [i.e., each accession derives from seeds obtained from a single mother plant, selected as representative of a particular natural population sampled from the ‘Monte’ phytogeographical region (Cavagnaro et al., 2006)], thereby facilitating their rapid inclusion in breeding programs, as opposed to plant materials with more complex genetic structures, such as ecotypes (Marinoni et al., 2020) or natural populations (Quiroga et al., 2013), used in other studies.

## 5 Conclusions

A broad and genetically diverse *T. crinita* collection was characterized for drought tolerance on the basis of quantitative morpho-physiological parameters, revealing significant and ample variation among the accessions for all the traits. Highly-tolerant and sensitive accessions were identified, and they will be used in future studies to investigate the genetic basis underlying drought tolerance in this species. Under prolonged drought conditions, the tolerant accessions were more productive for all the biomass yield components analyzed, and this seemed to be associated with a postponed and more attenuated decrease in variables related to the plant photosynthetic activity, such as stomatal conductance, chlorophyll content, and photochemical efficiency. The tolerant materials identified will be incorporated in breeding programs aiming at developing new varieties that combine drought tolerance with other traits of interest, such as high forage biomass yield and nutritional value, facilitating their widespread use as forage and revegetation of degraded drylands. Altogether, these data provide a platform for future studies and breeding programs for one of the most widely distributed grass species in arid environments of the Americas.

## Data availability statement

The original contributions presented in the study are included in the article/**Supplementary Material**. Further inquiries can be directed to the corresponding authors.

## Author contributions

DD, JC, and PC conceived the project. DD and JR performed the experiments. DD and AL performed most of the analyses. DD and PC wrote the draft. JC, YC, and PC edited the paper. All authors contributed to the article and approved the submitted version.

## References

[B1] Acquaah G. (2012). “Breeding for resistance to abiotic stresses,” in Principles of plant genetics and breeding. Ed. Acquaah G. (Hoboken, NJ: John Wiley and Sons), 280–330.

[B2] Aliche E. B. Theeuwen T. P. J. M. Oortwijn M. Visser R. G. F. van der Linden C. G. (2020). Carbon partitioning mechanisms in POTATO under drought stress. Plant Physiol. Biochem. 146, 211–219. doi: 10.1016/j.plaphy.2019.11.019 31756607

[B3] Anjum S. A. Ashraf U. Zohaib A. Tanveer M. Naeem M. Ali I.. (2017). Growth and developmental responses of crop plants under drought stress: A review. Zemdirb. Agric. 104, 267–276. doi: 10.13080/z-a.2017.104.034

[B4] Bazzaz F. A. (1996). Plants in changing environments: linking physiological, population, and community ecology (Cambridge, UK: Cambridge University Press).

[B5] Blum A. (2005). Drought resistance, water-use efficiency, and yield potential - are they compatible, dissonant, or mutually exclusive? Aust. J. Agric. Res. 56 (11), 1159–1168. doi: 10.1071/AR05069

[B6] Busso C. A. Fernández O. A. (2018). “Arid and semiarid rangelands of Argentina,” in Climate variability impacts on land use and livelihoods in drylands. Eds. Gaur M. Squires V. (Cham, Switzerland: Springer Nature Switzerland AG), 261–291. doi: 10.1007/978-3-319-56681-8_1

[B7] Campanella M. V. Bertiller M. B. (2008). Plant phenology, leaf traits and leaf litterfall of contrasting life forms in the arid Patagonian Monte, Argentina. J. Veg. Sci. 19, 75–85. doi: 10.3170/2007-8-18333

[B8] Carrizo I. M. López Colomba E. Tommasino E. Carloni E. Bollati G. Grunberg K. (2020). Contrasting adaptive responses to cope with drought stress and recovery in *Cenchrus ciliaris* L. and their implications for tissue lignification. Physiol. Plantarum 172, 1–18. doi: 10.1111/ppl.13274 33179274

[B9] Cavagnaro P. F. Cavagnaro J. B. Lemes J. L. Masuelli R. W. Passera C. B. (2006). Genetic diversity among varieties of the native forage grass *Trichloris crinita* based on AFLP markers, morphological characters, and quantitative agronomic traits. Genome 49, 906–918. doi: 10.1139/G06-060 17036066

[B10] Cavagnaro J. B. Trione S. O. (2007). Physiological, morphological and biochemical responses to shade of *Trichloris crinita*, a forage grass from the arid zone of Argentina. J. Arid Environ. 68, 337–347. doi: 10.1016/j.jaridenv.2006.06.004

[B11] Chapin F. S. (1980). The mineral nutrition of wild plants. Annu. Rev. Ecol. Evol. Syst. 11, 233–260. doi: 10.1146/annurev.es.11.110180.001313

[B12] Chaves M. M. Pereira J. S. Maroco J. Rodrigues M. L. Ricardo C. P. P. Osório M. L.. (2002). How plants cope with water stress in the field. Photosynthesis and growth. Ann. Bot. 89, 907–916. doi: 10.1093/aob/mcf105 12102516 PMC4233809

[B13] Chesson P. Gebauer R. Schwinning R. Huntly N. Wiegand K. Ernest. (2004). Resource pulses, species interactions, and diversity maintenance in arid and semi-arid ecosystems. Oecologia 141, 236–253. doi: 10.1007/s00442-004-1551-1 15069635

[B14] Couso L. L. Gatti M. L. Cornaglia P. S. Schrauf G. E. Fernández R. J. (2010). Are more productive varieties of *Paspalum dilatatum* less tolerant to drought? Grass Forage Sci. 65, 296–303. doi: 10.1111/j.1365-2494.2010.00748.x

[B15] Cui K. Huang J. Xing Y. Yu S. Xu C. Peng S. (2008). Mapping QTLs for seedling characteristics under different water supply conditions in rice (*Oryza sativa*). Physiol. Plant 132, 53–68. doi: 10.1111/j.1399-3054.2007.00991.x 18251870

[B16] De Martonne E. (1926). Une nouvelle fonction climatologique: L’indice d’aridité [A new climatological function: The aridity index]. La Meteorol. 2, 449–458.

[B17] Di Rienzo J. A. Casanoves F. Balzarini M. G. Gonzalez L. Tablada M. Robledo C. W. (2020). InfoStat versión 2020 (Argentina: Grupo InfoStat, FCA, Universidad Nacional de Córdoba). Available at: http://www.infostat.com.ar.

[B18] Di Rienzo J. A. Guzmán A. W. Casanoves F. (2002). A multiple-comparisons method based on the distribution of the root node distance of a binary tree. J. Agric. Biol. Environ. Stat. 7, 129–142. doi: 10.1198/10857110260141193

[B19] Di Rienzo J. A. Machiavelli R. Casanoves F. (2017). Modelos lineales mixtos: aplicaciones en InfoStat - 1a edición especial (Córdoba : InfoStat Transfer Center, FCA, National University of Cordoba).

[B20] Dominguez D. L. E. Cavagnaro J. B. Pérez M. B. Cavagnaro P. F. (2022). Plant dry weight and nutritive value of genetically diverse germplasm of false Rhodes grass [*Leptochloa crinita* (Lag.) P.M. Peterson and N.W. Snow], a native forage grass from arid regions of the Americas. Crop Sci. 62, 610–623. doi: 10.1002/csc2.20678

[B21] Fernandez R. J. Reynolds J. F. (2000). Potential growth and drought tolerance of eight desert grasses: lack of a trade-off? Oecologia 123 9090– 98. doi: 10.1007/s004420050993 28308749

[B22] Fick S. E. Hijmans R. J. (2017). WorldClim 2: New 1-km spatial resolution climate surfaces for global land areas. Int. J. Climatol. 37, 4302–4315. doi: 10.1002/joc.5086

[B23] Galmés J. Medrano H. Flexas J. (2007). Photosynthetic limitations in response to water stress and recovery in Mediterranean plants with different growth forms. New Phytol. 175, 81–93. doi: 10.1111/j.1469-8137.2007.02087.x 17547669

[B24] Gelli M. Konda A. R. Liu K. Zhang C. Clemente T. E. Holding D. R.. (2017). Validation of QTL mapping and transcriptome profiling for identification of candidate genes associated with nitrogen stress tolerance in sorghum. BMC Plant Biol. 17, 123. doi: 10.1186/s12870-017-1064-9 28697783 PMC5505042

[B25] Greco S. A. Cavagnaro J. B. (2003). Effects of drought on biomass production and allocation in three varieties of Trichloris crinita P. (Poaceae), a forage grass from the arid Monte region of Argentina. Plant Ecol. 164, 125–135. doi: 10.1023/A:1021217614767

[B26] Greco S. A. Cavagnaro J. B. (2005). Growth characteristics associated with biomass production in three varieties of *Trichloris crinita* (Poaceae), a forage grass native to the arid regions of Argentina. Rangeland J. 27, 135–142. doi: 10.1071/RJ05011

[B27] Guevara J. C. Grünwaldt E. G. Estevez O. R. Bisigato A. J. Blanco L. J. Biurrun F. N.. (2009). Range and livestock production in the Monte Desert, Argentina. J. Arid Environ. 73, 228–237. doi: 10.1016/j.jaridenv.2008.02.001

[B28] Gutierrez H. F. Richard G. A. Cerino M. C. (2016). Sistema reproductivo de *Trichloris* (Poaceae, Chloridoideae, Chlorideae). Bol. Soc Argent. Bot. 51, 111–122. doi: 10.31055/1851.2372.v51.n1.14421

[B29] Huston M. A. (1994). “Biological diversity,” in The coexistence of cpecies (Cambridge: UK: Cambridge University Press).

[B30] International Panel of Climatic Changes, (2014). In: Climate Change 2014: Impacts, Adaptation, and Vulnerability. Part A: Global and Sectoral Aspects. Contribution of Working Group II to the Fifth Assessment Report of the Intergovernmental Panel on Climate Change. Field, C. B., Barros, V. R., Dokken, D. J., Mach, K. J., Mastrandrea, M. D., Bilir, T. E., et al. (eds.). (Cambridge, United Kingdom and New York, NY, USA: Cambridge University Press) pp. 1–32.

[B31] Jung E. Y. Gaviria J. Sun S. Engelbrecht B. M. J. (2020). Comparative drought resistance of temperate grassland species: testing performance trade-offs and the relation to distribution. Oecologia 192, 1023–1036. doi: 10.1007/s00442-020-04625-9 32114638 PMC7165153

[B32] Kiranmayee K. N. S. U. Hash C. T. Sivasubramani S. Ramu P. Amindala B. P. Rathore A.. (2020). Fine-mapping of sorghum stay-green qtl on chromosome 10 revealed genes associated with delayed senescence. Genes 11, 1026. doi: 10.3390/genes11091026 32883037 PMC7565436

[B33] Kozub P. C. Barboza K. Cavagnaro J. B. Cavagnaro P. F. (2018a). Development and characterization of SSR markers for *Trichloris crinita* using sequence data from related grass species. Rev. Fac. Cienc. Agrar. 50, 1–16.

[B34] Kozub P. C. Barboza K. Galdeano F. Quarin C. Cavagnaro J. B. Cavagnaro P. F. (2017). Reproductive biology of the native forage grass *Trichloris crinita* (Poaceae; Chloridoideae). Plant Biol. 19, 444–453. doi: 10.1111/plb.12549 28135030

[B35] Kozub P. C. Cavagnaro J. B. Cavagnaro P. F. (2018b). Exploiting genetic and physiological variation of the native forage grass *Trichloris crinita* for revegetation in arid and semi-arid regions: An integrative review. Grass Forage Sci. 73, 257–271. doi: 10.1111/gfs.12337

[B36] Kozub P. C. Las Peñas M. L. Novo P. E. Cavagnaro P. F. (2019). Molecular cytogenetic characterization of the native forage grass *Trichloris crinita* . Crop Sci. 59, 1604–1616. doi: 10.2135/cropsci2018.12.0731

[B37] Manzoni S. Vico G. Katul G. Fay P. A. Polley W. Palmroth S.. (2011). Optimizing stomatal conductance for maximum carbon gain under water stress: A meta-analysis across plant functional types and climates. Funct. Ecol. 25, 456–467. doi: 10.1111/j.1365-2435.2010.01822.x

[B38] Marinoni L. Bortoluzzi A. Parra-Quijano M. Zabala J. M. Pensiero J. F. (2015). Evaluation and improvement of the ecogeographical representativeness of a collection of the genus *Trichloris* in Argentina. Genet. Resour. Crop Evol. 62, 593–604. doi: 10.1007/s10722-014-0184-4

[B39] Marinoni L. D. R. Richard G. A. Bustos D. Taleisnik E. L. Pensiero J. F. Zabala J. M. (2020). Differential response of *Trichloris* ecotypes from different habitats to drought and salt stress. Theor. Exp. Plant Physiol. 32, 213–229. doi: 10.1007/s40626-020-00182-x

[B40] Maxwell K. Johnson G. N. (2000). Chlorophyll fluorescence—a practical guide. J. Exp. Bot. 51, 659–668. doi: 10.1093/jexbot/51.345.659 10938857

[B41] Mitra J. (2001). Genetics and genetic improvement of drought resistance in crop plants. Curr. Sci. 80, 758–762.

[B42] Overpeck J. T. (2013). Climate science: The challenge of hot drought. Nature 503, 350–351. doi: 10.1038/503350a 24256801

[B43] Passera C. B. Borsetto O. Candia R. J. Stasi C. (1992). Shrub control and seeding influences on grazing capacity in Argentina. J. Range Manage. 45, 480–482. doi: 10.2307/4002906

[B44] Pendergast T.H.4. Qi P. Odeny D. A. Dida M. M. Devos K. M. (2022). A high-density linkage map of finger millet provides QTL for blast resistance and other agronomic traits. Plant Genome 15, e20175. doi: 10.1002/tpg2.20175 34904374 PMC12807240

[B45] Peterson P. M. Romaschenko K. Arrieta Y. H. (2015). A molecular phylogeny and classification of the Eleusininae with a new genus, *Micrachne* (Poaceae: Chloridoideae: Cynodonteae). Taxon 64, 445–467. doi: 10.12705/643.5

[B46] Peterson P. M. Romaschenko K. Snow N. Johnson G. (2012). A molecular phylogeny and classification of *Leptochloa* (Poaceae: Chloridoideae: Chlorideae) *sensu lato* and related genera. Ann. Bot. 109, 1317–1329. doi: 10.1093/aob/mcs077 22628365 PMC3359928

[B47] Poorter H. Nagel O. (2000). The role of biomass allocation in the growth response of plants to different levels of light, CO2, nutrients and water: a quantitative review. Aust. J. Plant Physiol. 27, 595–607. doi: 10.1071/pp99173_co

[B48] Poorter H. Niklas K. J. Reich P. B. Oleksyn J. Poot P. Mommer L. (2012). Biomass allocation to leaves, stems and roots: meta-analyses of interspecific variation and environmental control. New Phytol. 193, 30–50. doi: 10.1111/j.1469-8137.2011.03952.x 22085245

[B49] Popova L. P. Outlaw W. H. Aghoram K. Hite D. C. (2000). Abscisic acid—an intraleaf water-stress signal. Physiol. Plant 108, 376–381. doi: 10.1034/j.1399-3054.2000.t01-1-100406.x

[B50] Quiroga R. E. Fernández R. J. Golluscio R. A. Blanco L. J. (2013). Differential water-use strategies and drought resistance in *Trichloris crinita* plants from contrasting aridity origins. Plant Ecol. 214, 1027–1035. doi: 10.1007/s11258-013-0228-4

[B51] Quiroga R. E. Premoli A. C. Fernández R. J. (2018). Climatic niche shift in the amphitropical disjunct grass *Trichloris crinita* . PloS One 13, e0199811. doi: 10.1371/journal.pone.0199811 29953506 PMC6023228

[B52] Reynolds J. F. Smith D. M. S. Lambin E. F. Turner B. L. Mortimore M. Batterbury S. P.. (2007). Global desertification: building a science for dryland development. Science 316, 847–851. doi: 10.1126/science.1131634 17495163

[B53] Rich S. M. Watt M. (2013). Soil conditions and cereal root system architecture: review and considerations for linking Darwin and Weaver. J. Exp. Bot. 64, 1193–1208. doi: 10.1093/jxb/ert043 23505309

[B54] Seleiman M. F. Al-Suhaibani N. Ali N. Akmal M. Alotaibi M. Refay Y.. (2021). Drought stress impacts on plants and different approaches to alleviate its adverse effects. Plants 10, 259. doi: 10.3390/plants10020259 33525688 PMC7911879

[B55] Tahere A. S. Yamauchi A. Kamoshita A. Wade L. J. (2000). Genotypic variation in response of rainfed lowland rice to drought and rewatering. Plant Prod. Sci. 3, 180–188. doi: 10.1626/pps.3.180

[B56] USDA-NRCS (2020). Release brochure for Kinney Germplasm false Rhodes grass [Trichloris crinita (Lag.) Parodi] (Kingsville, Texas: USDA-Natural Resources Conservation Service, E. “Kika” de la Garza Plant Materials Center), 78363. Available at: https://nrcs.usda.gov/plantmaterials/stpmcrb13709.pdf.

[B57] Valliyodan B. Nguyen H. T. (2006). Understanding regulatory networks and engineering for enhanced drought tolerance in plants. Curr. Opin. Plant Biol. 9, 189–195. doi: 10.1016/j.pbi.2006.01.019 16483835

[B58] Villagra P. E. Passera C. B. Greco S. Sartor C. E. Meglioli P. A. Alvarez J. A. (2021). “Ecological restoration and productive recovery of saline environments from the argentine monte desert using native plants,” in Saline and alkaline soils in Latin America. Eds. Taleisnik E. Lavado R. S. (Cham, Switzerland: Springer Nature Switzerland AG), 309–324). doi: 10.1007/978-3-030-52592-7_17

[B59] Yao J. Liu H. Huang J. Gao Z. Wang G. Li D.. (2020). Accelerated dryland expansion regulates future variability in dryland gross primary production. Nat. Commun. 11, 1–10. doi: 10.1038/s41467-020-15515-2 32246032 PMC7125214

[B60] Zomer R. J. Xu J. Trabucco A. (2022). Version 3 of the global aridity index and potential evapotranspiration database. Sci. Data 9, 409. doi: 10.1038/s41597-022-01493-1 35840601 PMC9287331

