# Supplementary material for: Genetic diversity for drought tolerance in the native forage grass Trichloris crinita and possible morpho-physiological mechanisms involved
Source: Front Plant Sci. 2023 Aug 3;14:1235923. doi: 10.3389/fpls.2023.1235923 (PMC10435868; doi:10.3389/fpls.2023.1235923)
Supplement: Supplementary file 1 [file DataSheet_1.docx]

**SUPPLEMENTARY MATERIAL**

**Genetic diversity for drought tolerance in the native forage grass *Trichloris crinita* and possible morphophysiological mechanisms involved**

Deolindo L. E. Dominguez, Juan B. Cavagnaro, Juana Panasiti Ros, Anh Tuan Le, Yong Suk Chung, Pablo F. Cavagnaro

**Table S1.** Influence of the treatment, accession, sampling time, plant part, and their interactions on photoassimilates partitioning (%) to different plant organs (roots, stems, leaves, and panicles) for 21 *T. crinita* accessions grown in 2018

| **Factor** | **General**  **(n=2016)** | **Root**  **(n=504)** | **Stem**  **(n=504)** | **Leaf**  **(n=504)** | **Panicle**  **(n=504)** |
| --- | --- | --- | --- | --- | --- |
| Treatment (T) | - | 281.4*** | 139.7*** | 32.1*** | 288.8*** |
| Accession (A) | - | 569.2*** | 364.3*** | 392.5*** | 606.6*** |
| Sampling time (S) | - | 3425.2*** | 2118.7*** | 1736.9*** | 6532.4*** |
| Plant part (P) | 138005.6*** | - | - | - | - |
| T x A | - | 72.4*** | 55.9*** | 46.9*** | 42.4*** |
| T x S | - | 23.8*** | 21.9*** | 22.3*** | 37.1*** |
| T x P | 214.8*** | - | - | - | - |
| A x S | - | 29.1*** | 38.7*** | 29.4*** | 62.6*** |
| A x P | 554.5*** | - | - | - | - |
| S x P | 353.0*** | - | - | - | - |
| T x A x S | - | 11.5*** | 18.7*** | 9.3*** | 14.8*** |
| T x A x P | 68.9*** | - | - | - | - |
| T x S x P | 27.7*** | - | - | - | - |
| A x S x P | 42.1*** | - | - | - | - |
| T x A x S x P | 16.4*** | - | - | - | - |

Numbers are F values from ANOVA. Asterisks indicate statistically significant effects at p < 0.05 (*), p < 0.01 (**) and p < 0.001 (***). For the ANOVA, all the variables were expressed as percentage relative to the values in their respective irrigated Controls.

**Table S2.** Influence of the year, treatment, accession, sampling time, plant part, and their interactions on photoassimilates partitioning (%) to different plant organs (roots, stems, leaves, and panicles) for six *T. crinita* accessions over two years (2018 and 2019)

| **Factor** | **General**  **(n=1552)** | **Root**  **(n=288)** | **Stem**  **(n=288)** | **Leaf**  **(n=288)** | **Panicle**  **(n=288)** |
| --- | --- | --- | --- | --- | --- |
| Year (Y) | - | 4462.9*** | 3098.9*** | 20.6*** | 20.6*** |
| Treatment (T) | - | 80.1*** | 5.5* | 173.2*** | 173.2*** |
| Accession (A) | - | 1528.6*** | 816.6*** | 795.3*** | 795.3*** |
| Sampling time (S) | - | 530.7*** | 598.6*** | 1742.1*** | 1742.1*** |
| Plant part (P) | 65433.6*** | - | - | - | - |
| Y x T | - | 9.5** | ns | 33.5*** | 33.5*** |
| Y x A | - | 17.2*** | ns | 3.3** | 3.3** |
| Y x S | - | 1042.7*** | 81.7*** | 249*** | 249*** |
| Y x P | 2789.6*** | - | - | - | - |
| T x A | - | 98*** | 42.2*** | 36.6*** | 36.6*** |
| T x S | - | 22.9*** | 8.6*** | 20.8*** | 20.8*** |
| T x P | 96.3*** | - | - | - | - |
| A x S | - | 56.1*** | 34.8*** | 35.3*** | 35.3*** |
| A x P | 1245.7*** | - | - | - | - |
| S x P | 1237.9*** | - | - | - | - |
| Y x T x A | - | 12*** | 6.1*** | 8.9*** | 8.9*** |
| Y x T x S | - | ns | ns | 23.6*** | 23.6*** |
| Y x T x P | 14.5*** | - | - | - | - |
| Y x A x S | - | 7*** | 7.7*** | 19.5*** | 19.5*** |
| Y x A x P | 11.7*** | - | - | - | - |
| Y x S x P | 379.7*** | - | - | - | - |
| T x A x S | - | 18*** | 22.3*** | 13.4*** | 13.4*** |
| T x A x P | 63.1*** | - | - | - | - |
| T x S x P | 28.2*** | - | - | - | - |
| A x S x P | 60.5*** | - | - | - | - |
| Y x T x A x S | - | 7.7*** | 5.5*** | 7.7*** | 7.7*** |
| Y x T x A x P | 10.2*** | - | - | - | - |
| Y x T x S x P | 16.7*** | - | - | - | - |
| Y x A x S x P | 13*** | - | - | - | - |
| T x A x S x P | 21.6*** | - | - | - | - |
| Y x T x A x S x P | 7.9*** | - | - | - | - |

Numbers are F values from ANOVA. Asterisks indicate statistically significant effects at p < 0.05 (*), p < 0.01 (**) and p < 0.001 (***). ns, not significant. For the ANOVA, all the variables were expressed as percentage relative to the values in their respective irrigated Controls.

**Table S3.** Means comparisons for relative foliage/root ratio (RFRR) across accessions and time-points, for 2018 and 2019.

| Year | Acc. | 0 DAIDT | | 28 DAIDT | | 56 DAIDT | | | 84 DAIDT | |
| --- | --- | --- | --- | --- | --- | --- | --- | --- | --- | --- |
| 2018 | 1 | 100.6±5.7 | d | 135.2±23.4 | c | | 115.2±6.8 | c | 99.8±10.0 | d |
|  | 3 | 101.3±14.2 | d | 110.3±24.4 | c | | 140.8±9.2 | b | 123.8±3.2 | c |
|  | 4 | 103.3±2.9 | d | 61.1±2.4 | e | | 67.0±4.1 | e | 75.8±3.9 | e |
|  | 5 | 102.1±8.1 | d | 48.5±0.7 | f | | 69.9±1.0 | e | 75.6±10.4 | e |
|  | 6 | 102.9±3.6 | d | 145.9±5.8 | b | | 156.9±8.3 | b | 145.3±14.1 | b |
|  | 7 | 100.6±3.3 | d | 143.3±4.0 | b | | 116.5±2.0 | c | 119.1±2.0 | c |
|  | 8 | 101.3±13 | d | 105.8±17.3 | d | | 126.5±2.9 | c | 117.0±5.4 | c |
|  | 9 | 98.1±3.6 | d | 74.7±1.7 | e | | 84.3±6.1 | d | 97.6±2.8 | d |
|  | 10 | 105.0±1.1 | d | 158.6±24.3 | b | | 145.2±15.4 | b | 148.3±16.6 | b |
|  | 11 | 101.2±4.6 | d | 167.7±16.6 | a | | 152.5±4.9 | b | 148.4±0.5 | b |
|  | 12 | 101.9±2.2 | d | 122.0±8.0 | c | | 111.4±6.3 | c | 123.3±10.9 | c |
|  | 13 | 96.6±7 | d | 66.5±4.5 | e | | 72.9±4.3 | e | 88.4±1.5 | d |
|  | 14 | 99.7±2.2 | d | 138.3±10.0 | c | | 161.0±14.9 | b | 170.2±2.2 | a |
|  | 17 | 103.2±2.8 | d | 163.0±16.3 | b | | 176.7±9.5 | a | 146.0±2.2 | b |
|  | 18 | 104.3±9.4 | d | 84.3±1.1 | d | | 89.6±8.7 | d | 94.5±3.3 | d |
|  | 19 | 99.3±3.1 | d | 137.5±13.2 | c | | 127.9±1.2 | c | 129.5±10.0 | c |
|  | 20 | 97.2±5.5 | d | 128.1±14.8 | c | | 129.3±4.1 | c | 112.0±1.5 | c |
|  | 21 | 98.7±7.0 | d | 109.4±7.5 | c | | 121.4±27.5 | c | 155.9±8.1 | b |
|  | 22 | 102.3±1.4 | d | 132.9±17.6 | c | | 122.8±7.7 | c | 111.0±2.1 | c |
|  | 23 | 102.5±9.9 | d | 107.9±17.6 | d | | 106.4±3.5 | d | 104.7±7.0 | d |
|  | 24 | 102.3±5.2 | d | 95.5±7.2 | d | | 95.7±4.6 | d | 92.7±11.1 | d |
| 2019 | 1 | 99.2±9.9 | a | 83.5±14.2 | a | | 94.8±10.4 | a | 98.3±6.2 | a |
|  | 3 | 102.5±26.6 | a | 78.1±3.0 | a | | 121.0±13.9 | a | 117.4±2.9 | a |
|  | 5 | 103.7±7.7 | a | 61.4±3.7 | b | | 77.2±7.9 | a | 65.1±5.2 | b |
|  | 9 | 97.4±6.4 | a | 68.8±7.6 | b | | 84.9±11.3 | a | 85.7±5.8 | a |
|  | 18 | 100.9±7.6 | a | 93.8±14.6 | a | | 108.1±6.5 | a | 104.9±7.6 | a |
|  | 22 | 96.2±11.8 | a | 114.8±24.4 | a | | 100.5±9.9 | a | 81.6±2.8 | a |

Values are mean ± standard deviation of three biological replicates. For each year, means with a common letter are not significantly different at p<0.05, DGC test.

**Table S4.** Means comparisons for relative leaf area (RLA) across accessions and time-points, for 2018 and 2019.

| Year | Acc. | 0 DAIDT | | 28 DAIDT | | 56 DAIDT | | | 84 DAIDT | |
| --- | --- | --- | --- | --- | --- | --- | --- | --- | --- | --- |
| 2018 | 1 | 97.1±1.4 | a | 39.9±7.5 | d | | 46.6±4.2 | d | 29.8±4.2 | e |
|  | 3 | 97.6±1.6 | a | 40.5±6.2 | d | | 45.4±5.2 | d | 34.6±8.9 | e |
|  | 4 | 102.4±4.4 | a | 28.3±1.4 | e | | 35.6±3.7 | e | 19.7±3.2 | f |
|  | 5 | 99.3±5.5 | a | 30.9±1.8 | e | | 17.1±0.5 | f | 4±0.9 | g |
|  | 6 | 96.5±2.1 | a | 70.2±3.6 | b | | 63.4±6.8 | c | 16.8±10.1 | f |
|  | 7 | 102.3±4.5 | a | 45±2.1 | d | | 41±5 | d | 29.6±4.4 | e |
|  | 8 | 104.2±9 | a | 48±6.4 | d | | 29.3±1.4 | e | 8.4±1 | f |
|  | 9 | 104.1±7.6 | a | 33.3±2.3 | e | | 36.4±3.5 | e | 30.4±5.5 | e |
|  | 10 | 108±10.3 | a | 60±5 | c | | 50.7±2.1 | d | 9.2±1.2 | f |
|  | 11 | 103.9±1.7 | a | 61.7±5.4 | c | | 40.6±2.6 | d | 9±2.3 | f |
|  | 12 | 100±5 | a | 35.4±2.4 | e | | 14±2 | f | 11.8±2.4 | f |
|  | 13 | 97.9±0.7 | a | 45.9±4.1 | d | | 20.6±0.4 | f | 11.4±2.4 | f |
|  | 14 | 96.6±0.9 | a | 46±4.7 | d | | 27.9±1.9 | e | 19.7±2.4 | f |
|  | 17 | 101.6±1.6 | a | 50.5±12.6 | d | | 45.7±5.3 | d | 32.2±6.4 | e |
|  | 18 | 98±1.7 | a | 51.8±7.7 | d | | 40.8±1.9 | d | 18.9±7.7 | f |
|  | 19 | 102.1±4.1 | a | 32.7±3.4 | e | | 33.4±2.1 | e | 15.5±2.5 | f |
|  | 20 | 97.5±2.1 | a | 35.5±6.2 | e | | 32.8±2.2 | e | 27.1±2 | e |
|  | 21 | 100.2±1.6 | a | 73.3±5 | b | | 59±8.2 | c | 25±1.6 | e |
|  | 22 | 96.3±2.5 | a | 59.2±3.6 | c | | 33.5±1.5 | e | 29.5±2.1 | e |
|  | 23 | 97.9±4.1 | a | 48.9±8.3 | d | | 34.8±4.4 | e | 33.4±1.8 | e |
|  | 24 | 105.3±5.9 | a | 45.8±4.7 | d | | 32.8±4.3 | e | 15.4±1.5 | f |
| 2019 | 1 | 94.5±4.1 | b | 39.1±2 | f | | 38.6±3.4 | f | 39.2±1.3 | f |
|  | 3 | 94.5±10.4 | b | 47.1±5.3 | e | | 37.3±2.4 | f | 36.7±2.3 | f |
|  | 5 | 103.2±1.8 | b | 39.1±10.6 | f | | 16.3±1.6 | g | 12.3±1.8 | g |
|  | 9 | 116.9±21.7 | a | 54.3±3.4 | e | | 39.5±2.9 | f | 38.6±0.6 | f |
|  | 18 | 82.2±4 | c | 52.3±1.7 | e | | 38±1.1 | f | 24.9±2.5 | g |
|  | 22 | 110.1±9.7 | a | 65.1±4.7 | d | | 21.4±1.6 | g | 15.6±1 | g |

Values are mean ± standard deviation of three biological replicates. For each year, means with a common letter are not significantly different at p<0.05, DGC test.

**Table S5.** Means comparisons for relative chlorophyll index (RCI) across accessions and time-points, for 2018 and 2019.

| Year | Acc. | 0 DAIDT |  | 7 DAIDT |  | 14 DAIDT |  | 21 DAIDT |  | 28 DAIDT |  | 35 DAIDT |  | 42 DAIDT |  | 49 DAIDT |  | 56 DAIDT |  | 63 DAIDT |  | 70 DAIDT |  | 77 DAIDT |  | 84 DAIDT |  |
| --- | --- | --- | --- | --- | --- | --- | --- | --- | --- | --- | --- | --- | --- | --- | --- | --- | --- | --- | --- | --- | --- | --- | --- | --- | --- | --- | --- |
| 2018 | 1 | 99.3±0.7 | b | 100.2±7.4 | b | 99.9±10.1 | b | 99.1±6.3 | b | 95.3±1.9 | b | 91.5±6.8 | b | 92.4±5.5 | b | 87.6±5.2 | c | 78.7±0.8 | c | 77.4±0.8 | d | 63.9±3.9 | e | 44.7±3.9 | g | 36.2±5.0 | h |
|  | 3 | 96.5±5.4 | b | 101.4±4.9 | b | 97.2±6.9 | b | 92.2±1.7 | b | 87.9±0.7 | b | 86.5±6.3 | c | 85.4±6.1 | c | 83.6±2.0 | c | 82.2±0.6 | c | 71.0±4.4 | e | 62.7±2.7 | e | 47.8±5.4 | g | 38.7±4.9 | h |
|  | 4 | 99.3±2.4 | b | 98.8±6.8 | b | 93.1±3.7 | b | 89.9±2.6 | b | 84.2±2.3 | c | 85.2±6.3 | c | 79.6±8.0 | c | 79.3±13.1 | c | 67.7±3.5 | e | 66.8±4.5 | e | 55.7±1.0 | f | 31.9±3.6 | h | 36.2±1.1 | h |
|  | 5 | 101±3.4 | b | 94.4±5.8 | b | 94.7±6.8 | b | 90.6±3.9 | b | 85.3±4.3 | c | 88.6±2.8 | b | 82.8±3.8 | c | 67.9±6.1 | e | 55.4±3.9 | f | 30.1±3.9 | h | 10.1±7.5 | j | 10.1±8.3 | j | 8.4±1.4 | j |
|  | 6 | 99.5±6.9 | b | 90.4±4.1 | b | 91.9±2.7 | b | 96.2±2.1 | b | 90.1±1.2 | b | 87.9±5.3 | b | 96.6±4.9 | b | 67.1±3.2 | e | 65.8±2.7 | e | 58.7±4.6 | f | 27.5±4.6 | i | 23.5±2.6 | i | 24.1±2.2 | i |
|  | 7 | 99.3±2.5 | b | 98.2±2.0 | b | 94.2±2.4 | b | 93.9±2.3 | b | 89.6±1.8 | b | 90.5±2.6 | b | 86.7±0.8 | c | 76.6±5.4 | d | 72.9±3.6 | d | 73.1±5.4 | d | 62.8±2.5 | e | 25.1±1.4 | i | 22.9±1.8 | i |
|  | 8 | 100.6±0.6 | b | 91.3±1.4 | b | 93.1±0.8 | b | 94.3±2.7 | b | 91.5±4.6 | b | 94.2±5.1 | b | 91.8±3.6 | b | 91.4±2.2 | b | 79.3±5.8 | c | 80.1±2.1 | c | 59.0±3.2 | f | 26.3±5.1 | i | 22.3±2.4 | i |
|  | 9 | 99.9±3.6 | b | 98.3±2.5 | b | 102.1±2.2 | b | 105.2±5.8 | a | 93.5±1.1 | b | 92.2±0.8 | b | 99.2±0.9 | b | 94.6±1.8 | b | 97.6±0.8 | b | 91.8±1.4 | b | 70.8±4.0 | e | 54.7±4.4 | f | 45.1±3.3 | i |
|  | 10 | 100.4±0.9 | b | 96.6±2.2 | b | 92.1±5.1 | b | 90.6±5.5 | b | 89.4±2.0 | b | 90.7±0.8 | b | 92.9±2.4 | b | 78.1±3.2 | d | 69.7±2.6 | e | 58.7±2.1 | f | 44.5±4.4 | g | 34.0±1.9 | h | 20.4±2.7 | i |
|  | 11 | 100.2±2.8 | b | 99.2±5.1 | b | 99.3±3.2 | b | 94.2±1.9 | b | 89.9±2.5 | b | 89.9±3.3 | b | 84.2±5.1 | c | 83.5±2.0 | c | 73.5±2.1 | d | 69.2±2.4 | e | 65.5±1.5 | e | 40.1±5.5 | h | 19.8±2.8 | i |
|  | 12 | 101.4±4.4 | b | 93.1±0.6 | b | 93.3±3.9 | b | 88.2±2.3 | b | 86.7±1.6 | c | 77.2±5.5 | d | 86.3±2.8 | c | 75.3±3.5 | d | 74.1±4.2 | d | 68.5±0.6 | e | 64.9±3.4 | e | 31.6±2.5 | h | 18.4±2.3 | i |
|  | 13 | 99.2±1.1 | b | 92.7±0.6 | b | 92.9±4.1 | b | 95.3±1.2 | b | 94.3±2.2 | b | 95.7±5.9 | b | 88.9±0.4 | b | 86.1±3.2 | c | 73.9±2.7 | d | 64.0±1.8 | e | 55.7±3.5 | f | 35.4±3.1 | h | 18.1±2.8 | i |
|  | 14 | 99.4±3.5 | b | 94.9±5.2 | b | 90.2±3.7 | b | 88.2±3.8 | b | 90.5±3.9 | b | 84.2±2.8 | c | 90.8±1.7 | b | 80.9±0.5 | c | 62.3±3.5 | e | 58.7±1.3 | f | 52.4±3.7 | f | 32.8±3.7 | h | 17.8±2.5 | i |
|  | 17 | 99.3±3.2 | b | 93.9±6.5 | b | 92.3±4.6 | b | 89.3±2.6 | b | 88.1±1.6 | b | 81.7±1.6 | c | 83.7±5.5 | c | 75.8±3.8 | d | 66.3±2.5 | e | 65.2±4.4 | e | 57.6±3.6 | f | 29.7±2.8 | h | 17.2±0.2 | i |
|  | 18 | 98.2±10 | b | 97.2±5.5 | b | 96.1±4.5 | b | 98.6±4.2 | b | 94.9±4.1 | b | 86.7±4.6 | c | 79.5±3.9 | c | 75.9±5.1 | d | 63.4±4.1 | e | 53.4±3.3 | f | 34.3±2.2 | h | 15.2±2.4 | i | 12.7±2.4 | i |
|  | 19 | 99.5±2.9 | b | 91.2±7.2 | b | 85.5±3.7 | c | 86.8±4.1 | c | 84.8±2.2 | c | 84.2±1.5 | c | 79.9±4.2 | c | 80.7±0.9 | c | 63.1±2.3 | e | 52.5±2.3 | f | 39.5±1.4 | h | 19.8±3.1 | i | 16.1±2.9 | i |
|  | 20 | 98.4±2.9 | b | 95.7±4.3 | b | 94.5±4.5 | b | 86.8±1.7 | c | 87.5±4.5 | b | 90.5±4.7 | b | 89.1±3.2 | b | 84.6±2.2 | c | 65.4±3.3 | e | 56.2±2.3 | f | 52.4±3.2 | f | 23.7±2.4 | i | 15.5±3.4 | i |
|  | 21 | 100±4.2 | b | 89.8±0.5 | b | 84.3±3.4 | c | 83.4±2.8 | c | 83.1±0.3 | c | 83.6±2.7 | c | 83.1±2.4 | c | 72.6±5.4 | d | 60.7±2.4 | f | 50.6±4.1 | f | 38.9±4.0 | h | 17.2±2.1 | i | 15.5±2.1 | i |
|  | 22 | 97.5±3.9 | b | 101.4±1.9 | b | 106.3±4.3 | a | 98.4±4.9 | b | 92.6±2.9 | b | 89.3±7.8 | b | 86.2±4.4 | c | 70.7±4.8 | e | 67.3±1.1 | e | 58.9±4.4 | e | 50.1±6.3 | f | 16.8±2.1 | i | 13.2±2.5 | i |
|  | 23 | 100.1±4.8 | b | 98.6±1.3 | b | 96.5±1.3 | b | 90.1±2.4 | b | 88.1±4.7 | b | 84.9±1.1 | c | 94.5±2.4 | b | 75.2±5.4 | d | 74.1±2.8 | d | 64.6±1.8 | e | 57.8±4.2 | f | 30.8±2.6 | h | 14.1±5.8 | i |
|  | 24 | 99.9±4.7 | b | 90.3±2.5 | b | 91.2±0.6 | b | 92.7±1.7 | b | 95.5±1.2 | b | 93.2±3.6 | b | 88.2±1.1 | b | 72.4±2.5 | d | 75.7±3.2 | d | 69.1±0.6 | e | 57.5±5.7 | f | 33.5±2.0 | h | 9.4±7.1 | j |
| 2019 | 1 | 99.0±3.6 | b | 98.8±2.2 | b | 108.6±1.7 | a | 100.4±5.9 | b | 102.5±5.9 | b | 95.7±4.4 | b | 93.7±1.2 | b | 81.6±3.1 | c | 87.8±4.1 | c | 74.3±0.7 | d | 38.7±4.2 | f | 32.1±3.0 | f | 31.4±2.5 | f |
|  | 3 | 95.1±7.3 | b | 105.5±5.4 | b | 100.3±8.7 | b | 101.9±6.4 | b | 94.6±5.2 | b | 88.3±0.8 | c | 91.8±5.5 | b | 79.8±2.5 | c | 84.6±2.6 | c | 68.5±4.8 | d | 35.9±3.7 | f | 28.9±2.5 | f | 33.2±4.7 | f |
|  | 5 | 99.6±5.7 | b | 92.5±6.4 | b | 91.7±5.2 | b | 86.9±2.0 | c | 80.1±4.4 | c | 75.5±6.6 | d | 72.4±4.1 | d | 62.7±2.6 | d | 50.8±4.2 | e | 29.3±4.3 | f | 8.8±8.3 | g | 4.2±2.4 | h | 2.9±0.1 | h |
|  | 9 | 101.4±1.0 | b | 102.5±2.8 | b | 102.5±0.4 | b | 108.3±1.3 | a | 106.1±4.6 | b | 90.7±5.8 | b | 96.1±5.6 | b | 91.6±3.4 | b | 95.5±7.0 | b | 85.9±5.1 | c | 49.8±4.7 | e | 38.9±2.3 | f | 37.1±3.4 | f |
|  | 18 | 103.8±5.8 | b | 97.9±2.4 | b | 91.7±7.1 | b | 95.7±2.8 | b | 90.7±4.2 | b | 76.1±6.2 | d | 74.8±2.1 | d | 73.4±0.3 | d | 64.6±3.6 | d | 36.6±3.6 | f | 11.3±3.2 | g | 10.5±3.3 | g | 14.2±3.7 | g |
|  | 22 | 98.8±5.6 | b | 99.3±2.8 | b | 96.9±4.8 | b | 101.8±5.5 | b | 90.9±3.8 | b | 85.7±3.8 | c | 75.9±4.5 | d | 71.2±1.5 | d | 66.9±4.4 | d | 52.2±3.5 | e | 16.7±2.4 | g | 13.9±1.9 | g | 10.2±7.0 | g |

Values are mean ± standard deviation of three biological replicates. For each year, means with a common letter are not significantly different at p<0.05, DGC test.

**Table S6.** Means comparisons for relative photochemical efficiency (RPE) of photosystem II across accessions and time-points, for 2018 and 2019

| Year | Acc. | 0 DAIDT |  | 7 DAIDT |  | 14 DAIDT |  | 21 DAIDT |  | 28 DAIDT |  | 35 DAIDT |  | 42 DAIDT |  | 49 DAIDT |  | 56 DAIDT |  | 63 DAIDT |  | 70 DAIDT |  | 77 DAIDT |  | 84 DAIDT |  |
| --- | --- | --- | --- | --- | --- | --- | --- | --- | --- | --- | --- | --- | --- | --- | --- | --- | --- | --- | --- | --- | --- | --- | --- | --- | --- | --- | --- |
| 2018 | 1 | 99.1±1.6 | a | 100.3±2.0 | a | 102.4±4 | a | 98.1±2.2 | a | 106.8±11.7 | a | 96.9±2.4 | a | 104.8±1.2 | a | 96.8±2.1 | a | 95.9±1.9 | a | 93.4±3.5 | a | 86.6±4.0 | a | 81.4±3.6 | b | 78.2±7.8 | b |
|  | 3 | 101.3±2.2 | a | 99.1±2.2 | a | 100±2.9 | a | 100.3±1 | a | 104.7±5.1 | a | 98.8±2.6 | a | 99.9±3.0 | a | 98.5±3.4 | a | 102.4±2.6 | a | 93.2±1.4 | a | 92.9±4.2 | a | 92±3.6 | a | 85±7.5 | a |
|  | 4 | 100.4±0.7 | a | 101.6±4.4 | a | 97.4±6.2 | a | 99.3±2.1 | a | 96.9±3.5 | a | 99.3±2.8 | a | 104.6±4.8 | a | 87.5±2.8 | a | 96.2±6.4 | a | 86.0±1.5 | a | 69.9±12.5 | b | 42.7±22.5 | c | 10.8±14 | e |
|  | 5 | 99.6±0.8 | a | 97.9±3.6 | a | 100±2.8 | a | 98.3±2.5 | a | 100.1±1.1 | a | 94.7±2.2 | a | 93.8±4.6 | a | 80.4±1.2 | b | 78.1±5.0 | b | 35.9±18.5 | d | 9.7±11.5 | e | 1.9±1.6 | e | 0.0±0.0 | e |
|  | 6 | 103.2±5.5 | a | 102.1±2.6 | a | 96.0±4.2 | a | 99.1±0.9 | a | 99.8±2.2 | a | 98.7±4.4 | a | 103.9±1.0 | a | 92.8±5.6 | a | 90.8±3.8 | a | 34.7±17.1 | d | 2.6±1.4 | e | 0.5±0.8 | e | 0.9±1.6 | e |
|  | 7 | 99.5±0.8 | a | 99.2±3.6 | a | 107.7±1.5 | a | 108.5±6.9 | a | 103.4±4.6 | a | 98.3±2.5 | a | 110.1±2.8 | a | 92.6±4.2 | a | 84.0±9.4 | a | 80.2±6.5 | b | 54.9±14.3 | c | 45.4±7.6 | c | 45.8±27.3 | c |
|  | 8 | 99.4±1.0 | a | 98.2±4.1 | a | 95.2±6.3 | a | 98.7±2.2 | a | 102.1±4.3 | a | 100.1±3.3 | a | 101.3±5.0 | a | 94.1±2.0 | a | 96.1±3.7 | a | 85.8±6.4 | a | 79.4±7.6 | b | 39.5±12.5 | d | 12.6±12.9 | e |
|  | 9 | 100.3±0.5 | a | 100.2±1.7 | a | 103.2±3.1 | a | 100.1±2.8 | a | 99.1±1.2 | a | 98.5±4.0 | a | 105.7±3.0 | a | 89.2±2.6 | a | 92.3±4.4 | a | 93.2±1.3 | a | 87.8±6.4 | a | 73.6±9.3 | b | 68.7±6.0 | b |
|  | 10 | 102.6±4.4 | a | 103.1±4.9 | a | 104.8±2.7 | a | 99.6±4.7 | a | 105.3±2.7 | a | 105±2.5 | a | 108.4±2.3 | a | 91.1±3.0 | a | 96.4±2.6 | a | 68.7±26.5 | b | 52.6±41.1 | c | 35.5±18.4 | d | 33.0±20.7 | d |
|  | 11 | 99.7±0.6 | a | 101.8±3.2 | a | 103.4±2.8 | a | 101.7±2.4 | a | 105.9±2.5 | a | 98.3±1.2 | a | 102.0±6.4 | a | 88.7±3.6 | a | 90.6±5.9 | a | 93.7±3.3 | a | 91.1±9.2 | a | 58.6±14.8 | c | 44.0±17.0 | c |
|  | 12 | 99.6±0.6 | a | 100.8±4.1 | a | 102.1±4.0 | a | 96.5±2.5 | a | 101.0±4.9 | a | 100.7±3.6 | a | 110.7±3.6 | a | 84.6±4.4 | a | 86.6±3.1 | a | 52.2±13.2 | c | 8.9±6.8 | e | 7.8±7.7 | e | 0.9±1.5 | e |
|  | 13 | 98.7±2.2 | a | 95.4±4.3 | a | 97.4±6.9 | a | 97.5±1.4 | a | 101.7±2.6 | a | 98.6±0.8 | a | 111.4±3.5 | a | 87.9±3.5 | a | 93.9±6.1 | a | 93.6±2.8 | a | 93.3±10.9 | a | 52.6±22.2 | c | 14.6±9.5 | e |
|  | 14 | 100.9±1.6 | a | 95.7±1.4 | a | 101.9±4.6 | a | 101.5±1.6 | a | 101.9±4.2 | a | 99.8±2.8 | a | 109.9±1.8 | a | 89.1±4.2 | a | 93.0±6.5 | a | 94.5±7.4 | a | 86.9±6.1 | a | 59.6±21 | c | 35.9±9.7 | d |
|  | 17 | 99.8±0.4 | a | 99.7±3.7 | a | 101.0±1.6 | a | 101.8±4.5 | a | 103.8±6.9 | a | 99.3±2.1 | a | 101.7±1.6 | a | 94.1±0.5 | a | 97.5±3.9 | a | 59.7±13.8 | c | 18.9±18.3 | e | 6.6±9.2 | e | 13.5±11.1 | e |
|  | 18 | 100.5±0.8 | a | 104.7±1.8 | a | 102.7±4.9 | a | 96.5±2.2 | a | 106.3±4.3 | a | 100.3±2.9 | a | 103.4±5.3 | a | 90.0±3.5 | a | 88.6±4.9 | a | 25.0±11.7 | d | 4.0±1.5 | e | 2.3±2.1 | e | 0.5±0.8 | e |
|  | 19 | 98.8±2.1 | a | 97.7±3.3 | a | 95.4±6.7 | a | 99.3±1.2 | a | 101.0±2.8 | a | 103.4±0.7 | a | 107.7±3.3 | a | 87.5±3.4 | a | 91.2±4.7 | a | 49.0±11.3 | c | 8.7±6.8 | e | 6.3±8.5 | e | 2.3±2.8 | e |
|  | 20 | 99.1±1.8 | a | 98.1±4.5 | a | 91.1±8.8 | a | 96.3±1.8 | a | 101.2±4.6 | a | 98.7±3.2 | a | 107.8±6.1 | a | 91.9±3.7 | a | 90.2±0.2 | a | 58.7±18.4 | c | 34.9±20.1 | d | 19.8±8.5 | e | 1.0±0.9 | e |
|  | 21 | 100.4±0.6 | a | 98.3±5.2 | a | 95.5±3.1 | a | 98.6±4.0 | a | 104.1±1.9 | a | 97.7±0.5 | a | 109.3±3.4 | a | 92.4±5.5 | a | 97.6±1.0 | a | 51.9±11.0 | c | 8.5±7.5 | e | 9.0±12.1 | e | 2.7±1.4 | e |
|  | 22 | 100.8±1.4 | a | 103.4±0.9 | a | 96.8±3.5 | a | 96.6±4.1 | a | 102.1±2.7 | a | 101.6±3.3 | a | 108.5±5.4 | a | 92.6±5.1 | a | 90.5±5.2 | a | 28.3±11.0 | d | 15.6±20.0 | e | 7.9±7.6 | e | 2.2±2.0 | e |
|  | 23 | 99.1±1.5 | a | 100.3±3.3 | a | 95.2±4.3 | a | 98.4±1.1 | a | 100.5±5 | a | 103.9±4.8 | a | 108.6±2.3 | a | 88.0±3.5 | a | 92.5±8.7 | a | 54.4±22.6 | c | 31.8±25.4 | d | 13.8±11.2 | e | 9.4±11.6 | e |
|  | 24 | 100.1±0.2 | a | 101±2.4 | a | 96.8±4.9 | a | 100.4±3.1 | a | 101.5±3.4 | a | 104.3±1.5 | a | 101.5±3.4 | a | 94.0±1.7 | a | 96.4±6.3 | a | 37.5±20.7 | d | 3.5±1.5 | e | 0.9±0.8 | e | 1.0±0.9 | e |
| 2019 | 1 | 98.1±4.9 | a | 99.9±1.8 | a | 99.2±3.8 | a | 98.4±2.2 | a | 96.5±1.1 | a | 99.0±2.6 | a | 105.0±4.9 | a | 96.1±1.5 | a | 94.3±4.0 | a | 92.3±1.1 | a | 84.7±4.5 | a | 66.5±5.2 | c | 59.9±6.9 | c |
|  | 3 | 103.9±4.8 | a | 93.2±0.8 | a | 99.4±1.2 | a | 98.1±8.9 | a | 103.8±0.9 | a | 98.3±1.2 | a | 100.2±3.5 | a | 93.0±3.8 | a | 99.6±3.5 | a | 94.3±5.5 | a | 92.7±1.1 | a | 87.1±2.9 | a | 63.2±10.0 | c |
|  | 5 | 101.8±1.1 | a | 98.5±3.6 | a | 97.3±3.6 | a | 97.6±2.5 | a | 98.9±2.0 | a | 94.0±1.5 | a | 94.7±1.6 | a | 80.3±3.0 | b | 75.4±9.2 | b | 30.7±10.2 | d | 8.9±6.8 | e | 7.8±7.7 | e | 0.0±0.0 | e |
|  | 9 | 102.2±3.4 | a | 103.9±1.3 | a | 99.3±1.7 | a | 101.4±3.5 | a | 102.1±1.4 | a | 97.8±1.0 | a | 108.3±2.0 | a | 92.1±4.6 | a | 93.9±6.1 | a | 91.9±1.9 | a | 84.2±5.1 | a | 58.3±15.1 | c | 41.8±11.8 | d |
|  | 18 | 100.8±3.5 | a | 101.5±4.7 | a | 77.6±9.7 | b | 100.1±2.8 | a | 103.4±3.1 | a | 102.2±3.0 | a | 102.8±0.9 | a | 89.1±4.2 | a | 90.2±2.0 | a | 29.1±10.1 | d | 8.2±12.5 | e | 5.6±9.6 | e | 0.0±0.0 | e |
|  | 22 | 101.7±2.9 | a | 97.7±3.0 | a | 94.4±3.7 | a | 97.6±8.0 | a | 102.2±3.6 | a | 100.6±1.9 | a | 105.1±3.5 | a | 89.1±0.5 | a | 85.7±2.0 | a | 25.6±3.4 | d | 6.2±8.8 | e | 6.7±9.2 | e | 1.8±2.1 | e |

Values are means ± standard deviation of three biological replicates. For each year, means with a common letter are not significantly different at p<0.05, DGC test.

**Table S7.** Means comparisons for relative stomatal conductance (Rg_s_) across accessions and time-points, for 2018 and 2019

| Year | Acc. | 0 DAIDT |  | 7 DAIDT |  | 14 DAIDT |  | 21 DAIDT |  | 28 DAIDT |  | 35 DAIDT |  | 42 DAIDT |  | 49 DAIDT |  | 56 DAIDT |  | 63 DAIDT |  | 70 DAIDT |  | 77 DAIDT |  | 84 DAIDT |  |
| --- | --- | --- | --- | --- | --- | --- | --- | --- | --- | --- | --- | --- | --- | --- | --- | --- | --- | --- | --- | --- | --- | --- | --- | --- | --- | --- | --- |
| 2018 | 1 | 101.8±9.1 | a | 36.1±2.6 | d | 30.3±1.6 | d | 34.8±2.7 | d | 35.7±2.3 | d | 42.1±1.5 | c | 41.8±0.4 | c | 34.5±3.7 | d | 39.3±3.2 | d | 46.1±7.6 | c | 57.6±2.0 | b | 50.2±1.2 | c | 47.0±6.6 | c |
|  | 3 | 100.0±2.7 | a | 29.1±2.1 | d | 33.1±3.8 | d | 38.8±3.0 | d | 37.2±3.4 | d | 42.0±0.6 | c | 37.3±3.0 | d | 36.5±3.4 | d | 35.9±2.4 | d | 45.8±7.3 | c | 59.0±1.1 | b | 61.9±5.8 | b | 63.1±10.9 | b |
|  | 4 | 100.5±4.3 | a | 55.1±4.3 | b | 48.4±2.5 | c | 35.4±2.4 | d | 22.8±1.8 | e | 23.0±1.8 | e | 19.8±0.7 | e | 19.2±1.6 | e | 17.9±2.1 | e | 17.0±1.3 | e | 14.0±2.6 | e | 11.6±2.9 | e | 13.1±3.1 | e |
|  | 5 | 100.9±4.9 | a | 53.8±3.4 | b | 47.2±1.7 | c | 33.5±3.8 | d | 29.5±4.3 | d | 33.2±3.4 | d | 36.6±3.2 | d | 23.0±4.4 | e | 21.4±5.3 | e | 11.4±11.0 | e | 12.2±13 | e | 11.7±20.3 | e | 11.3±21.4 | e |
|  | 6 | 100.2±2.7 | a | 63.7±3.1 | b | 43.3±3.5 | c | 38.6±2.0 | d | 37.3±15.6 | d | 24.3±2.5 | e | 15.8±1.1 | e | 26.0±2.4 | e | 22.3±1.1 | e | 29.6±3.1 | d | 25.3±4.8 | e | 29.6±3.1 | d | 30.2±4.9 | d |
|  | 7 | 100.0±4.1 | a | 34.8±2.4 | d | 24.9±2.0 | e | 30.3±0.7 | d | 30.6±1.9 | d | 31.0±1.2 | d | 32.1±1.3 | d | 39.6±3.0 | d | 36.7±2.6 | d | 33.6±2.7 | d | 31.5±3.3 | d | 29.9±1.0 | d | 28.4±3.0 | d |
|  | 8 | 100.9±3.1 | a | 58.6±3.9 | b | 39.7±4.2 | d | 30.1±2.3 | d | 26.1±2.0 | e | 24.2±1.5 | e | 28.7±4.9 | d | 26.7±4.3 | e | 24.0±2.2 | e | 31.9±0.3 | d | 28.2±3.3 | d | 22.4±3.1 | e | 29.1±2.5 | d |
|  | 9 | 99.7±6.1 | a | 37.6±1.5 | d | 33.2±0.5 | d | 32.5±1.8 | d | 32.1±1.1 | d | 36.9±3.0 | d | 37.1±2.5 | d | 35.4±3.2 | d | 26.1±3.3 | e | 28.0±3.6 | d | 26.4±2.0 | e | 26.0±2.3 | e | 24.3±4.3 | e |
|  | 10 | 100.2±1.3 | a | 45.0±1.2 | c | 37.7±3.9 | d | 41.4±1.8 | c | 39.3±2.7 | d | 35.0±1.2 | d | 31.6±2.0 | d | 29.2±4.1 | d | 28.1±1.8 | d | 34.3±1.4 | d | 41.7±5.3 | c | 46.7±3.0 | c | 46.2±1.2 | c |
|  | 11 | 99.4±3.2 | a | 36.8±2.3 | d | 26.3±2.6 | e | 35.3±3.4 | d | 31.7±1.6 | d | 34.6±2.1 | d | 32.0±2.1 | d | 32.7±2.0 | d | 32.2±2.7 | d | 32.8±0.4 | d | 32.1±1.4 | d | 35.6±2.3 | d | 35.9±3.2 | d |
|  | 12 | 103.3±4.1 | a | 56.6±1.1 | b | 44.9±4.4 | c | 32.7±4.0 | d | 21.9±3.5 | e | 19.2±2.4 | e | 21.8±2.0 | e | 13.2±2.1 | e | 16.8±1.7 | e | 13.9±2.7 | e | 25.6±3.2 | e | 23.9±9.9 | e | 18.2±4.7 | e |
|  | 13 | 101.3±1.7 | a | 50.4±1.6 | c | 49.4±2.7 | c | 38.8±1.4 | d | 28.4±7.4 | d | 22.1±2.6 | e | 23.8±1.7 | e | 21.1±1.0 | e | 18.1±1.2 | e | 16.7±1.4 | e | 13.5±1.0 | e | 18.4±1.8 | e | 15.6±3.5 | e |
|  | 14 | 100.6±1.7 | a | 43.5±3.4 | c | 39.3±0.5 | d | 36.2±2.5 | d | 34.9±2.2 | d | 32.9±3.4 | d | 30.9±2.3 | d | 29.4±1.9 | d | 25.9±1.6 | e | 26.4±1.2 | e | 25.2±1.5 | e | 28.1±0.6 | d | 29.3±2.7 | d |
|  | 17 | 100.1±3.3 | a | 54.0±2.3 | b | 37.7±2.5 | d | 32.2±3.6 | d | 27.7±1.9 | d | 21.9±1.7 | e | 24.9±1.8 | e | 31.1±2.3 | d | 22.3±1.2 | e | 17.4±1.6 | e | 16.1±1.9 | e | 22.2±3.3 | e | 16.1±2.2 | e |
|  | 18 | 100.5±3.2 | a | 67.8±2.9 | b | 58.9±0.5 | b | 33.5±4.3 | d | 29.7±4.3 | d | 28.6±3.7 | d | 26.7±1.9 | e | 24.0±2.3 | e | 20.4±1.9 | e | 19.7±2.3 | e | 16.4±2.9 | e | 17.5±3.9 | e | 14±13.5 | e |
|  | 19 | 100.1±2.1 | a | 53.9±3.5 | b | 36.8±2.7 | d | 32.0±4.5 | d | 28.9±2.7 | d | 21.6±2.4 | e | 31.8±4.0 | d | 29.5±2.3 | d | 24.1±2.3 | e | 17.3±3.4 | e | 17.7±1.7 | e | 12.5±2.0 | e | 15.8±1.9 | e |
|  | 20 | 101.8±2.3 | a | 62.7±2.1 | b | 57.2±5.1 | b | 40.7±3.3 | c | 25.8±1.8 | e | 27.3±3.7 | d | 21.9±2.1 | e | 20.6±2.3 | e | 18.5±2.2 | e | 19.4±2.4 | e | 14.8±2.0 | e | 12.4±2.1 | e | 12.3±0.5 | e |
|  | 21 | 99.7±4.2 | a | 57.9±3.0 | b | 51.5±4.5 | c | 36.1±4.1 | d | 28.3±1.8 | d | 25.2±3.8 | e | 33.2±4.0 | d | 36.2±4.4 | d | 28.1±1.8 | d | 25.5±4.4 | e | 23.5±2.6 | e | 32.3±2.3 | d | 18.7±3.3 | e |
|  | 22 | 100.4±5.1 | a | 63.1±4.5 | b | 60.0±4.6 | b | 44.1±4.6 | c | 31.6±2.6 | d | 26.8±3.6 | e | 23.1±1.1 | e | 19.1±2.7 | e | 24.2±2.7 | e | 25.4±2.5 | e | 17.0±3.7 | e | 19.2±3.7 | e | 16.8±3.4 | e |
|  | 23 | 100.1±4.6 | a | 24.4±0.8 | e | 30.5±1.0 | d | 34.5±2.9 | d | 31.2±1.8 | d | 29.9±2.8 | d | 34.8±3.1 | d | 32.8±1.7 | d | 26.6±2.5 | e | 25.7±1.8 | e | 20.2±2.0 | e | 20.5±0.4 | e | 25.5±2.3 | e |
|  | 24 | 101.2±3.4 | a | 27.5±1.8 | d | 33.9±2.7 | d | 42.5±3.6 | c | 38.6±3.0 | d | 33.8±2.1 | d | 28.6±2.5 | d | 34.9±2.8 | d | 28.3±2.2 | d | 25.8±2.5 | e | 25.5±1.7 | e | 21.9±4.1 | e | 21.9±2.8 | e |
| 2019 | 1 | 100.3±6.1 | a | 33.4±2.9 | d | 29.8±3.0 | d | 33.1±2.7 | d | 39.9±2.9 | c | 44.8±2.0 | c | 44.8±2.5 | c | 41.2±3.3 | c | 38.3±3.7 | c | 43.1±1.9 | c | 59.6±7.2 | b | 48.5±5.5 | c | 43.3±5.9 | c |
|  | 3 | 100.1±5.0 | a | 28.8±2.2 | d | 27.3±1.6 | d | 33.8±2.9 | d | 37.1±2.2 | c | 44.5±1.3 | c | 43.5±0.9 | c | 39.6±1.7 | c | 35.3±3.1 | d | 47.1±4.1 | c | 63.5±4.8 | b | 59.5±0.4 | b | 42.4±6.3 | c |
|  | 5 | 97.2±2.4 | a | 49.6±3.2 | c | 45.6±1.2 | c | 33.1±2.7 | d | 28.3±2.3 | d | 31.4±2.5 | d | 33.3±1.4 | d | 23.3±2.3 | e | 13.7±6.0 | f | 7.0±5.2 | f | 3.3±5.6 | f | 5.9±6.8 | f | 7.4±0.4 | f |
|  | 9 | 93.2±1.5 | a | 35.0±3.2 | d | 34.1±2.0 | d | 33.7±2.7 | d | 33.7±2.3 | d | 38.2±2.2 | c | 42.3±2.9 | c | 34.5±1.6 | d | 26.5±3.0 | d | 26.2±2.8 | d | 26.6±1.6 | d | 23.2±2.4 | e | 26.1±3.9 | d |
|  | 18 | 98.1±2.7 | a | 62.5±5.0 | b | 59.4±2.4 | b | 33.3±4.2 | d | 27.3±3.0 | d | 28.2±1.7 | d | 26.1±1.0 | d | 23.4±1.4 | e | 18.1±3.2 | e | 13.9±2.2 | f | 9.5±5.9 | f | 11.6±8.7 | f | 11.6±6.9 | f |
|  | 22 | 100.8±3.6 | a | 60.3±2.7 | b | 56.7±3.9 | b | 43.7±3.0 | c | 26.1±2.1 | d | 29.5±2.8 | d | 22.7±1.2 | e | 20.4±1.8 | e | 22.1±1.8 | e | 22.5±2.8 | e | 11.0±6.1 | f | 15.4±3.6 | e | 14.2±4.9 | f |

Values are mean ± standard deviation of three biological replicates. For each year, means with a common letter are not significantly different at p<0.05, DGC test.

**Table S8.** Means comparisons for relative number of panicles per plant (RNPP) across accessions and time-points, for 2018 and 2019

| Year | Acc. | 0 DAIDT |  | 7 DAIDT |  | 14 DAIDT |  | 21 DAIDT |  | 28 DAIDT |  | 35 DAIDT |  | 42 DAIDT |  | 49 DAIDT |  | 56 DAIDT |  | 63 DAIDT |  | 70 DAIDT |  | 77 DAIDT |  | 84 DAIDT |  |
| --- | --- | --- | --- | --- | --- | --- | --- | --- | --- | --- | --- | --- | --- | --- | --- | --- | --- | --- | --- | --- | --- | --- | --- | --- | --- | --- | --- |
| 2018 | 1 | 100.0±0.0 | f | 83.3±16.7 | g | 65.7±9.9 | h | 78.7±1.2 | g | 82.0±1.1 | g | 78.4±2.3 | g | 73.0±4.4 | h | 69.0±3.4 | h | 65.1±1.7 | h | 70.1±1.6 | h | 74.2±1.5 | h | 76.8±0.6 | g | 78.9±0.4 | g |
|  | 3 | 100.0±0.0 | f | 103.3±5.8 | f | 84.2±14.5 | g | 77.3±6.3 | g | 86.1±3.8 | g | 84.8±1.0 | g | 84.8±1.6 | g | 74.4±2.0 | h | 70.1±2.3 | h | 74.9±1.7 | h | 78.8±0.5 | g | 83.2±0.7 | g | 85.2±1.2 | g |
|  | 4 | 100.0±0.0 | f | 183.3±28.9 | a | 168.2±16.8 | b | 101.6±4.6 | f | 75.2±1.7 | h | 73.7±1.9 | h | 68.8±4.0 | h | 73.3±0.7 | h | 78.5±2.8 | g | 78.5±1.3 | g | 79.5±1.3 | g | 77.9±2.1 | g | 77.8±1.7 | g |
|  | 5 | 100.0±0.0 | f | 31.1±10.2 | j | 32.0±4.8 | j | 23.3±4.5 | k | 26.4±3.2 | k | 20.9±4.2 | k | 18.0±1.1 | k | 17.0±1.6 | k | 14.7±0.9 | k | 15.7±1.4 | k | 15.5±1.0 | k | 15.2±0.9 | k | 15.2±0.9 | k |
|  | 6 | 100.0±0.0 | f | 72.5±12.3 | h | 65.3±12.2 | h | 83.7±4.3 | g | 119.4±5.8 | e | 103.3±5.8 | f | 86.3±1.6 | g | 97.9±0.1 | f | 101.2±1.1 | f | 103.1±1.0 | f | 101.8±1.8 | f | 99.4±1.1 | f | 98.9±1.1 | f |
|  | 7 | 91.7±14.4 | f | 119.8±21.6 | e | 123.6±10.5 | e | 121.8±10.3 | e | 155.2±14.3 | c | 110.3±2.7 | e | 87.9±3.5 | g | 81.6±1.1 | g | 79.4±3.3 | g | 81.7±2.6 | g | 81.9±2.1 | g | 81.3±2.5 | g | 81.1±2.1 | g |
|  | 8 | 100.0±0.0 | f | 85.0±13.2 | g | 84.1±16.7 | g | 70.9±7.9 | h | 53.4±4.8 | i | 66.9±6.7 | h | 79.1±4.9 | g | 69.7±3.7 | h | 66.8±3.6 | h | 65.5±1.4 | h | 63.7±2.6 | h | 62.4±0.9 | h | 60.9±1.1 | h |
|  | 9 | 100.0±0.0 | f | 62.4±7.3 | h | 62.8±6.7 | h | 72.4±3.8 | h | 73.8±2.2 | h | 64.2±1.2 | h | 55.4±1.5 | i | 60.0±0.8 | i | 61.8±0.6 | h | 63±0.7 | h | 62.6±0.6 | h | 61.9±0.3 | h | 61.3±1.4 | h |
|  | 10 | 93.3±11.5 | f | 90.2±2.5 | f | 96.0±3.5 | f | 113.1±9.5 | e | 131.0±2.1 | d | 111.6±0.7 | e | 91.6±2.0 | f | 94.7±3.2 | f | 94.3±1.1 | f | 94.4±1.1 | f | 92.4±0.9 | f | 91.4±0.5 | f | 88.5±0.4 | g |
|  | 11 | 93.3±11.5 | f | 94.4±9.6 | f | 102.4±4.1 | f | 116.0±7.1 | e | 125.4±8.4 | e | 104.8±2.6 | f | 96.9±3.8 | f | 85.5±0.9 | g | 78.3±1.5 | g | 79.0±1.7 | g | 79.3±1.7 | g | 79.6±1.7 | g | 78.9±1.1 | g |
|  | 12 | 100.0±0.0 | f | 108.9±8.4 | e | 94.9±12.4 | f | 69.6±4.0 | h | 87.0±1.6 | g | 87.8±3.9 | g | 88.9±3.2 | g | 83.3±1.9 | g | 80.1±1.6 | g | 80.3±1.2 | g | 79.5±1.5 | g | 79.3±1.1 | g | 78.4±0.6 | g |
|  | 13 | 100.0±0.0 | f | 80.0±20.0 | g | 75.5±4.3 | h | 74.6±0.6 | h | 79.8±4.1 | g | 74.3±1.1 | h | 66.7±2.6 | h | 68.8±1.0 | h | 65.9±1.0 | h | 67.2±1.2 | h | 67.8±0.7 | h | 67.7±1.0 | h | 67.4±1.3 | h |
|  | 14 | 100.0±0.0 | f | 83.6±3.8 | g | 83.8±7.1 | g | 66.9±7.9 | h | 99.8±5.6 | f | 83.6±4.6 | g | 68.4±2.0 | h | 63.6±1.3 | h | 59.4±0.4 | i | 69.5±1.8 | h | 69.9±0.5 | h | 70.8±0.3 | h | 71.1±1.3 | h |
|  | 17 | 93.3±11.5 | f | 76.1±3.5 | h | 93.7±13.2 | f | 93.9±3.7 | f | 77.3±3.1 | g | 70.5±1.3 | h | 67.4±1.8 | h | 74.9±1.5 | h | 76.8±1.5 | g | 81.8±0.5 | g | 85.9±1.2 | g | 85.7±1.7 | g | 84.8±1.6 | g |
|  | 18 | 100.0±0.0 | f | 48.2±7.1 | i | 45.2±6.5 | i | 56.5±1.9 | i | 54.5±3.3 | i | 57.4±3.5 | i | 58.4±3.2 | i | 60.1±0.8 | i | 62.3±0.9 | h | 63.1±1.5 | h | 62.8±1.5 | h | 62.5±1.6 | h | 62.5±1.6 | h |
|  | 19 | 102.8±29.3 | f | 58.9±8.4 | i | 48.1±10.5 | i | 64.1±1.6 | h | 72.7±4.8 | h | 70.8±4.1 | h | 69.3±1.6 | h | 64.8±1.6 | h | 63.8±1.4 | h | 70.2±1.7 | h | 71.8±0.5 | h | 72.9±0.7 | h | 73.2±0.5 | h |
|  | 20 | 100.0±0.0 | f | 81.3±5.6 | g | 99.5±11.8 | f | 87.1±4.7 | g | 78.2±2.5 | g | 81.2±4.4 | g | 85.1±6.3 | g | 70.7±2.6 | h | 56.3±1.6 | i | 58.2±0.5 | i | 58.3±0.8 | i | 58.0±0.5 | i | 59.2±1.1 | h |
|  | 21 | 91.7±14.4 | f | 105.6±9.6 | f | 114.6±6.8 | e | 68.8±2.8 | h | 84.3±2.9 | g | 77.3±2.7 | g | 71.0±2.3 | h | 74.2±0.4 | h | 77.0±1.2 | g | 82.5±0.4 | g | 85.2±1.2 | g | 86.4±1.3 | g | 86.1±1.6 | g |
|  | 22 | 100.0±0.0 | f | 85±13.2 | g | 81.1±9.6 | g | 76.0±2.7 | h | 91.0±1.5 | f | 113.6±7.5 | e | 101.5±1.3 | f | 97.1±2.7 | f | 71.7±2.3 | h | 71.2±1.8 | h | 71.0±1.5 | h | 70.4±1.1 | h | 70.4±1.1 | h |
|  | 23 | 100.0±0.0 | f | 144.3±5.2 | c | 120.5±8.4 | e | 88.5±5.2 | g | 99.6±7.3 | f | 93.1±2.1 | f | 86.0±1.8 | g | 76.6±0.3 | g | 72.2±1.4 | h | 71.6±1.6 | h | 72.4±1.6 | h | 72.2±1.1 | h | 71.9±0.9 | h |
|  | 24 | 100.0±0.0 | f | 95.2±8.3 | f | 92.5±6.6 | f | 102.0±3.4 | f | 90.9±2.1 | f | 82.4±3.5 | g | 79.0±3.9 | g | 72.0±1.8 | h | 67.6±1.8 | h | 67.9±1.4 | h | 69.6±1.8 | h | 70.2±2.4 | h | 69.9±2.0 | h |
| 2019 | 1 | 100.0±0.0 | a | 87.8±10.7 | b | 59.1±4.9 | e | 64.1±4.1 | d | 77.4±4.2 | c | 76.5±2.2 | c | 71.3±0.5 | d | 68.6±1.1 | d | 70.3±1.6 | d | 73.0±1.1 | c | 73.7±0.7 | c | 74.1±0.3 | c | 75.2±1.1 | c |
|  | 3 | 100.0±0.0 | a | 104.2±7.2 | a | 85.8±8.5 | b | 73.5±3 | c | 69.1±5.2 | d | 63.7±1.3 | d | 54.8±1.0 | f | 55.8±0.4 | f | 66.3±1.9 | d | 67.7±1.8 | d | 70.9±0.5 | d | 75.7±0.7 | c | 76.1±0.3 | c |
|  | 5 | 100.0±0.0 | a | 28.9±7.7 | h | 31.9±6.4 | g | 34.9±4.4 | g | 48.3±8.0 | f | 47.9±1.8 | f | 38.2±3.8 | g | 34.5±2.3 | g | 28.4±2.5 | h | 27.6±2.4 | h | 27.4±2.1 | h | 26.8±2.1 | h | 26.8±2.0 | h |
|  | 9 | 100.0±0.0 | a | 64.1±7.6 | d | 67.6±3.5 | d | 72.4±3.8 | c | 53.6±0.6 | f | 60.1±0.2 | e | 50.8±0.8 | f | 52.0±1.3 | f | 57.5±1.2 | e | 52.4±0.5 | f | 53.0±0.5 | f | 52.9±0.9 | f | 61.3±1.4 | e |
|  | 18 | 100.0±0.0 | a | 48.5±2.6 | f | 44.3±1.3 | f | 50±1.5 | f | 54.9±3.2 | f | 46.4±1.4 | f | 48.6±6.6 | f | 50.9±1.5 | f | 51.7±0.8 | f | 51.7±0.4 | f | 51.9±1.6 | f | 51.2±1.6 | f | 51.2±1.6 | f |
|  | 22 | 100.0±0.0 | a | 70.7±12.0 | d | 43.7±5.5 | f | 69.7±5.5 | d | 77.6±3.0 | c | 77.5±2.3 | c | 65.5±1.8 | d | 57.0±1.7 | e | 54.9±0.1 | f | 53.6±0.1 | f | 54.1±0.6 | f | 53.9±0.7 | f | 53.9±0.7 | f |

Values are mean ± standard deviation of three biological replicates. For each year, means with a common letter are not significantly different at p<0.05, DGC test.

**Supplementary Table S9.** Pearson correlation analysis of aridity indices and annual precipitation in the accessions collection sites with phenotypic traits associated with drought tolerance.

| **Phenotypic traits *vs.* aridity index (De Martonne 1926)** | | | | | | | | |
| --- | --- | --- | --- | --- | --- | --- | --- | --- |
|  | **Year 1** | | | | **Year 2** | | | |
| **Phenotypic** | **56 DAIDT** | | **84 DAIDT** | | **56 DAIDT** | | **84 DAIDT** | |
| **Trait** | **r** | **p** | **r** | **p** | **r** | **p** | **r** | **p** |
| RTDM | -0.10 | 0.418 | -0.09 | 0.482 | -0.10 | 0.701 | -0.14 | 0.568 |
| Aerial RDM | -0.08 | 0.515 | -0.08 | 0.557 | -0.07 | 0.771 | -0.11 | 0.675 |
| Root RDM | -0.02 | 0.849 | -0.15 | 0.247 | 0.21 | 0.403 | -0.32 | 0.201 |
| RFRR | -0.05 | 0.699 | -0.02 | 0.850 | 0.32 | 0.194 | 0.44 | 0.066 |
| RLA | -0.19 | 0.135 | -0.14 | 0.261 | 0.32 | 0.201 | 0.23 | 0.350 |
| RCI | 0.06 | 0.636 | 0.07 | 0.591 | -0.07 | 0.772 | 0.08 | 0.761 |
| RPE | -0.13 | 0.317 | 0.10 | 0.425 | 0.12 | 0.626 | -0.22 | 0.390 |
| RGs | 0.00 | 0.976 | 0.02 | 0.862 | -0.25 | 0.322 | -0.22 | 0.390 |
| RNPP | **-0.26** | **0.038** | -0.25 | 0.052 | -0.06 | 0.801 | -0.06 | 0.809 |
| **Phenotypic traits *vs.* aridity index (Zomer et al. 2022)** | | | | | | | | |
| **Phenotypic**  **Trait** | **Year 1**  **Year 1**  **Year 1** | | | | **Year 2** | | | |
|  | **56 DAIDT**  **84 DAIDT** | | **84 DAIDT**  **84 DAIDT** | | **56 DAIDT**  **84 DAIDT** | | **84 DAIDT**  **84 DAIDT** | |
|  | **r** | **p** | **r** | **p** | **r** | **p** | **r** | **p** |
| RTDM | -0.05 | 0.689 | -0.03 | 0.846 | -0.22 | 0.372 | -0.26 | 0.295 |
| Aerial RDM | -0.04 | 0.783 | 0.00 | 0.991 | -0.20 | 0.439 | -0.23 | 0.351 |
| Root RDM | -0.06 | 0.643 | 0.16 | 0.204 | 0.42 | 0.080 | -0.35 | 0.149 |
| RFRR | 0.08 | 0.523 | 0.13 | 0.316 | 0.36 | 0.148 | 0.29 | 0.240 |
| RLA | -0.06 | 0.616 | -0.12 | 0.351 | 0.03 | 0.914 | -0.11 | 0.660 |
| RCI | -0.03 | 0.789 | -0.10 | 0.428 | -0.29 | 0.241 | -0.14 | 0.570 |
| RPE | 0.20 | 0.116 | -0.24 | 0.058 | 0.01 | 0.957 | **-0.52** | **0.026** |
| RGs | -0.03 | 0.799 | -0.09 | 0.484 | -0.38 | 0.115 | **-0.47** | **0.048** |
| RNPP | 0.07 | 0.583 | 0.01 | 0.927 | -0.11 | 0.656 | -0.18 | 0.481 |
| **Phenotypic traits *vs.* mean annual precipitation** | | | | | | | | |
| **Phenotypic**  **Trait** | **Year 1**  **Year 1**  **Year 1** | | | | **Year 2** | | | |
|  | **56 DAIDT**  **84 DAIDT** | | **84 DAIDT**  **84 DAIDT** | | **56 DAIDT** | | **84 DAIDT** | |
|  | **r** | **p** | **r** | **p** | **r** | **p** | **r** | **p** |
| RTDM | -0.07 | 0.594 | -0.06 | 0.657 | -0.07 | 0.780 | -0.12 | 0.626 |
| Aerial RDM | -0.06 | 0.631 | -0.05 | 0.677 | -0.05 | 0.854 | -0.09 | 0.736 |
| Root RDM | 0.04 | 0.777 | -0.12 | 0.369 | 0.21 | 0.411 | -0.29 | 0.237 |
| RFRR | -0.08 | 0.516 | -0.06 | 0.625 | 0.34 | 0.167 | 0.46 | 0.056 |
| RLA | -0.20 | 0.113 | -0.13 | 0.319 | 0.33 | 0.184 | 0.24 | 0.328 |
| RCI | 0.09 | 0.500 | 0.09 | 0.486 | -0.05 | 0.844 | 0.10 | 0.687 |
| RPE | -0.12 | 0.353 | 0.09 | 0.483 | 0.15 | 0.558 | -0.21 | 0.405 |
| RGs | 0.01 | 0.977 | 0.01 | 0.914 | -0.23 | 0.352 | -0.21 | 0.411 |
| RNPP | **-0.25** | **0.049** | -0.23 | 0.065 | -0.04 | 0.860 | -0.04 | 0.872 |

RTDM. relative total dry matter; Aerial RDM. relative dry matter of the aerial plant parts; Root RDM. root relative dry matter; RFRR. relative foliage/root ratio; RLA. relative leaf area; RCI. relative chlorophyll index; RPE. relative photochemical efficiency (RPE) of photosystem II, RGs. relative stomatal conductance; RNPP. relative number of panicles per plant. Significant correlations are indicated in bold.

**
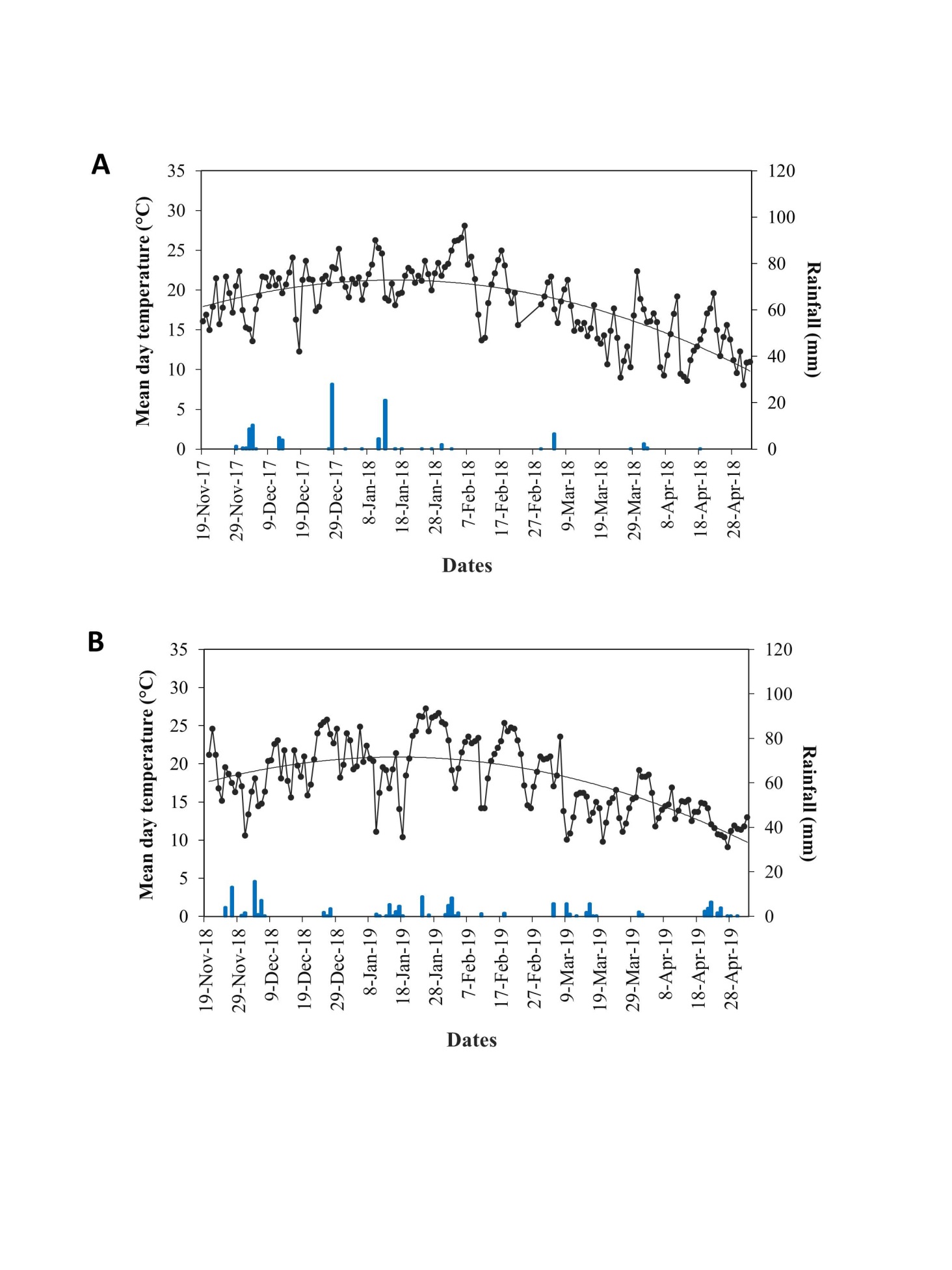
**

**Figure S1.** Time-course variation for mean day temperature (with adjusted trend line) and rainfall (blue bars) during the experiments of 2017-2018 (A) and 2018-2019 (B) at Lujan de Cuyo, Mendoza, Argentina.

**
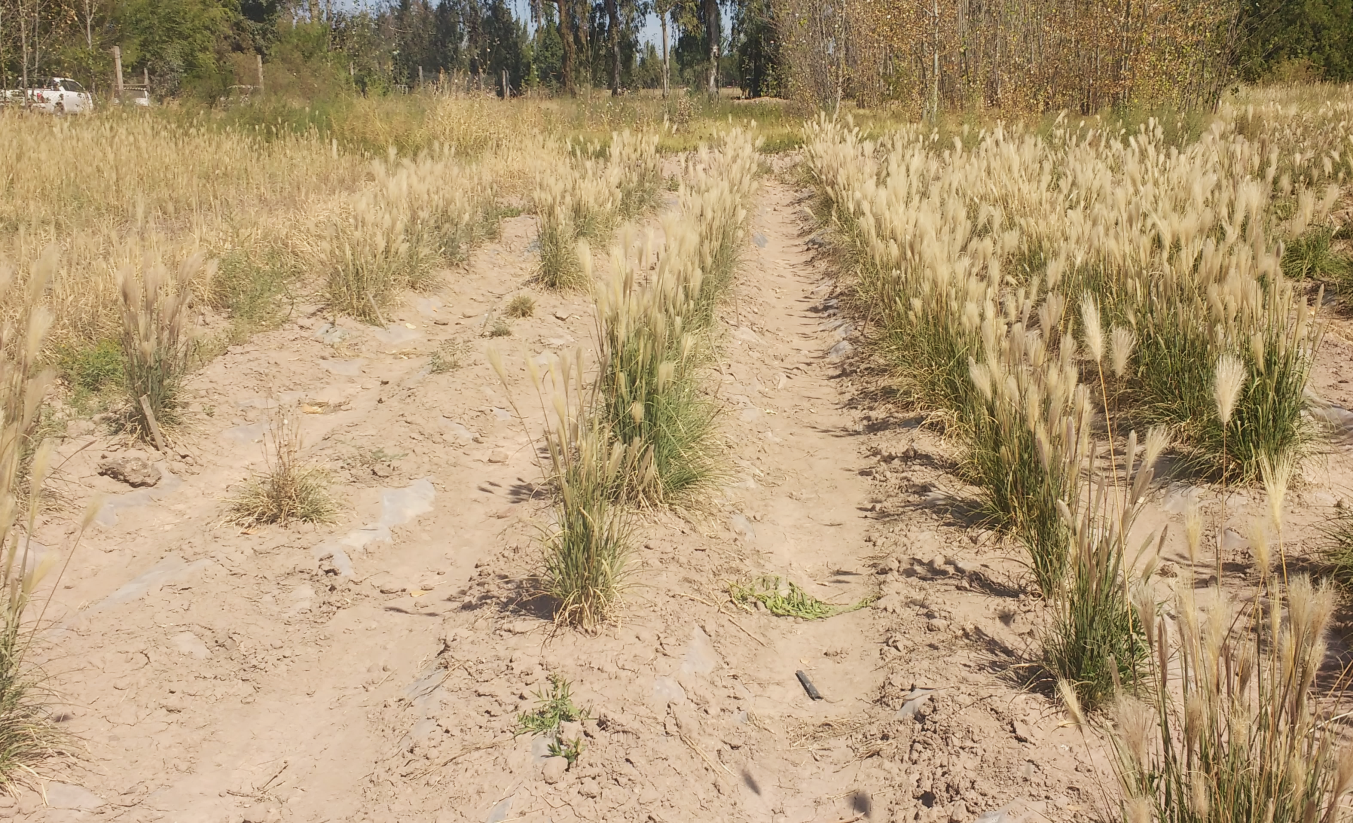
**

**Figure S2.** Field trial for evaluating drought tolerance in 21 *Trichloris crinita* accessions in the drought treatment plot at 28 DAIDT (108 days after the transplant).

**
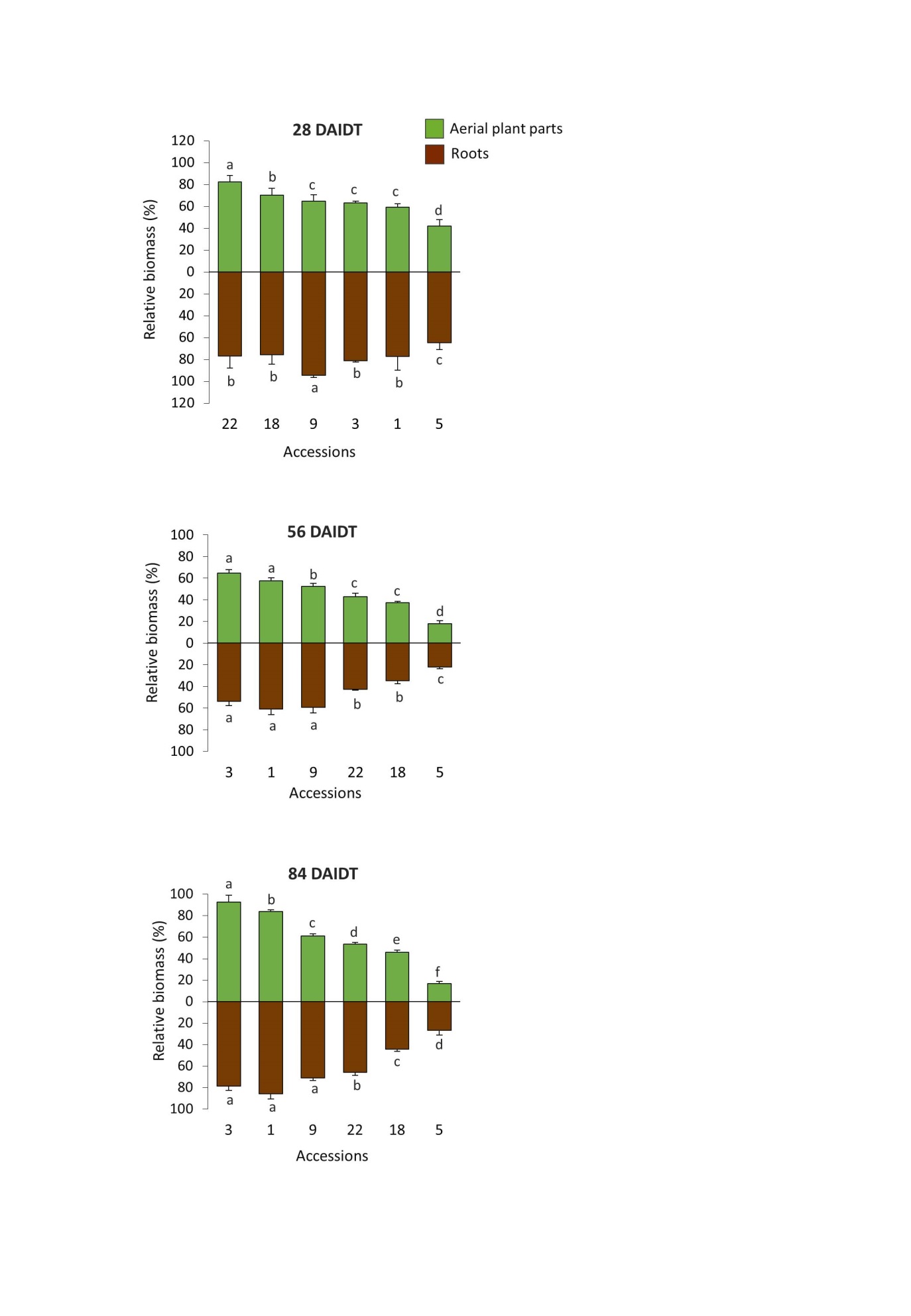
**

**Figure S3**. Time-course variation for relative biomass in the aerial part of the plant and in the roots of six *T. crinita* accessions grown under drought conditions in 2019, expressed as percentage of dry matter (DM) in these plant parts in the respective irrigated Control plants, according to the formula (DM_Drought_/DM_Irrigated_) × 100. Dry matter of the aerial part of the plant is the sum of DM values for stems, leaves, and panicles. Bars represent means of three biological replicates ± standard deviations. Data for 28, 56, and 84 days after initiation of the drought treatment (DAIDT) are presented, whereas baseline data (0 DAIDT) are not shown because all mean values were approximately 100% and not statistically different from each other. For each time point, data are presented in decreasing order based on the accessions DM content in the aerial part of the plant. Bars not sharing a common letter are significantly different at p < 0.05, DGC test.

**
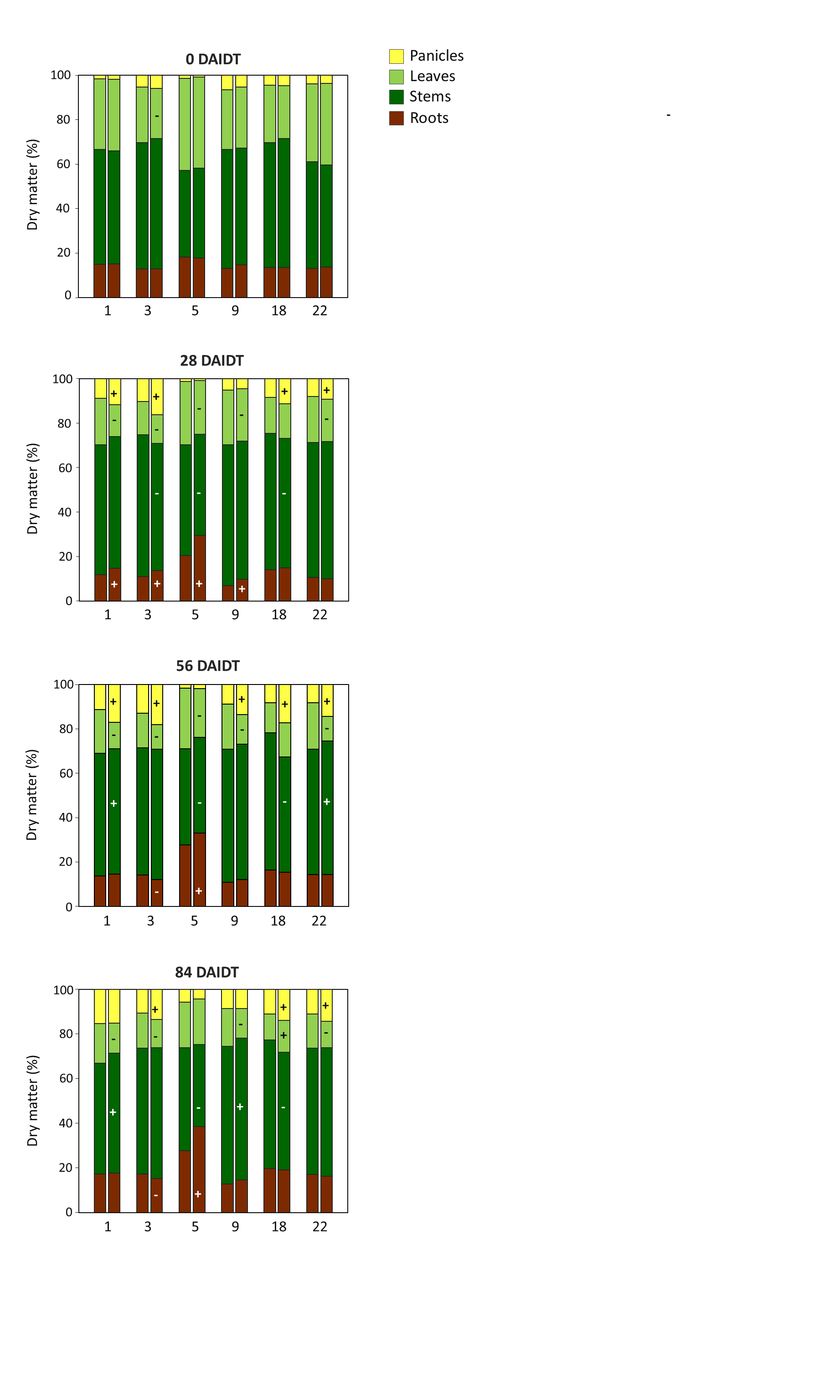
**

**Figure S4.** Percentage of the total dry matter (DM) per plant partitioned to different organs before the drought treatment (0 DAIDT), and 28, 56, and 84 days after initiation of the drought treatment (DAIDT) for six *T. crinita* accessions grown under irrigated (left bar) and drought conditions (right bar) in 2019. Plus (+) and minus (-) symbols in the drought treatment bar indicate significant increase and decrease in DM partitioning to a particular organ, respectively, relative to the irrigated (Control) plants, at p < 0.05, DGC test.


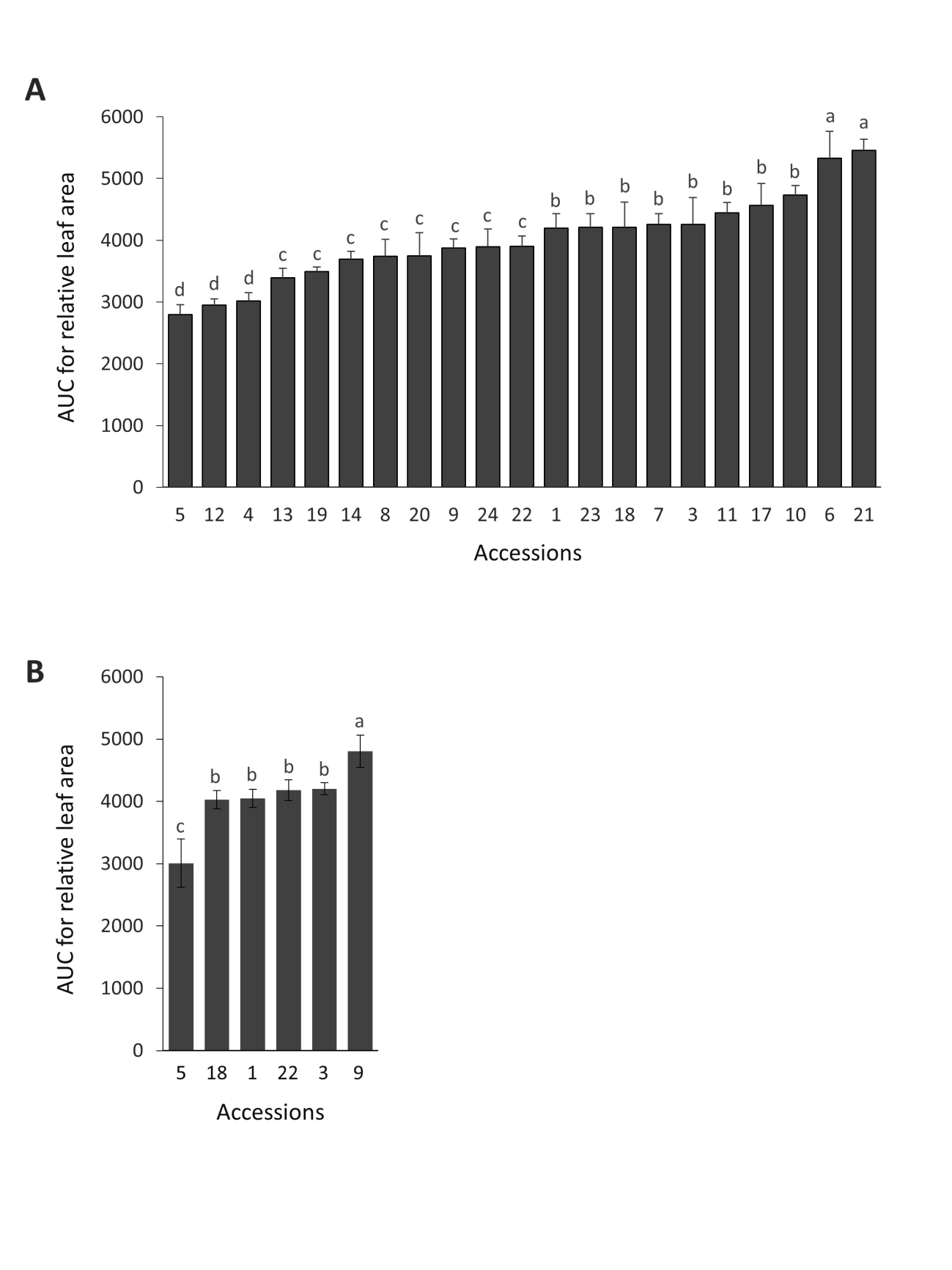


**Figure S5.** Area under the curve (AUC) for relative leaf area (RLA) per plant for 21 *T. crinita* accessions grown under drought conditions in 2018 (A), and six accessions in 2019 (B). RLA is expressed as percentage of the total leaf area in the respective irrigated Controls, as calculated by the formula: RLA = (LA_Drought_/LA_Irrigated_) × 100; and the AUC was estimated from the graphs in Fig. 3. Bars indicate mean AUC ± standard deviation. Bars with the same letters are not significantly different at p<0.05, DGC test.

**
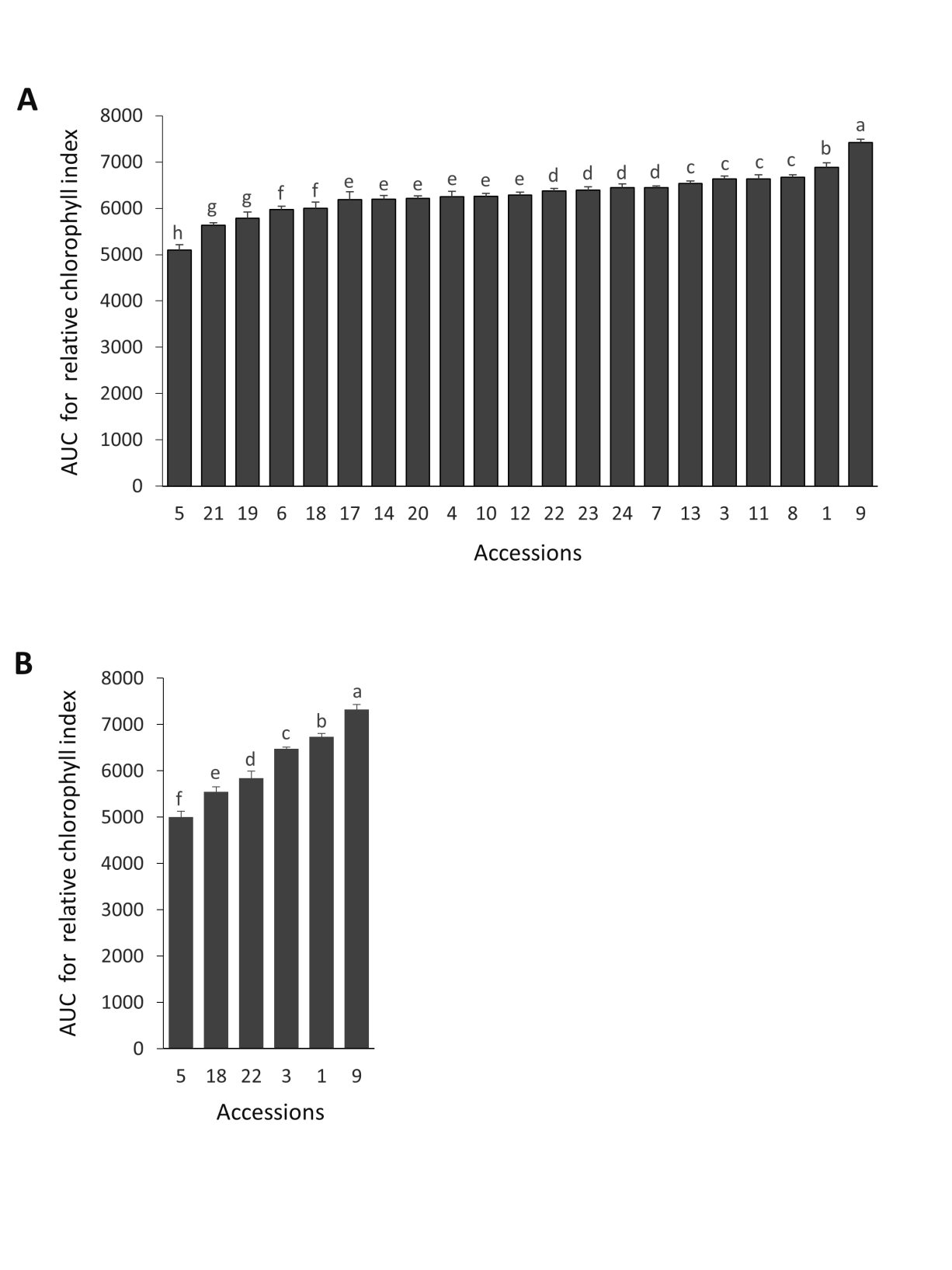
**

**Figure S6.** Area under the curve (AUC) for relative chlorophyll index (RCI) for 21 *T. crinita* accessions grown under drought conditions in 2018 (A), and six accessions in 2019 (B). RCI is expressed as percentage of the chlorophyll index in the respective irrigated Controls, as calculated by the formula: RCI = (CI_Drought_/CI_Irrigated_) × 100; and the AUC was estimated from the graphs in Fig. 4. Bars indicate mean AUC ± standard deviation. Bars with the same letters are not significantly different at p<0.05, DGC test.

**
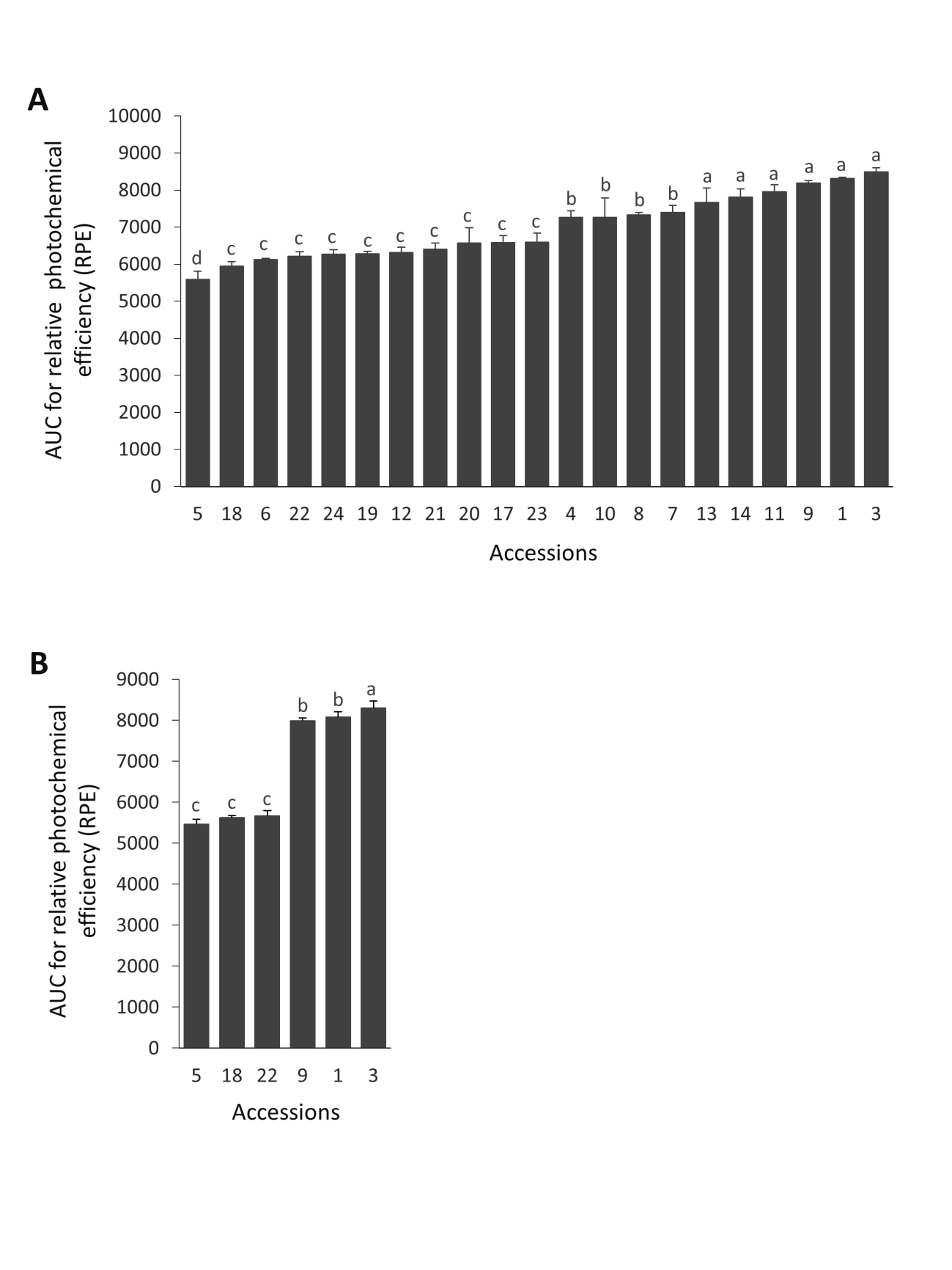
**

**Figure S7.** Area under the curve (AUC) for relative photochemical efficiency (RPE) of photosystem II (estimated by the Fv/Fm ratio) in leaves of 21 *T. crinita* accessions grown under drought conditions in 2018 (A), and six accessions in 2019 (B). RPE is expressed as percentage of the photochemical efficiency (IPE) in the respective irrigated Controls, as calculated by the formula: RPE = (PE_Drought_/PE_Irrigated_) × 100; and the AUC was estimated from the graphs in Fig. 5. Bars indicate mean AUC ± standard deviation. Bars with the same letters are not significantly different at p<0.05, DGC test.


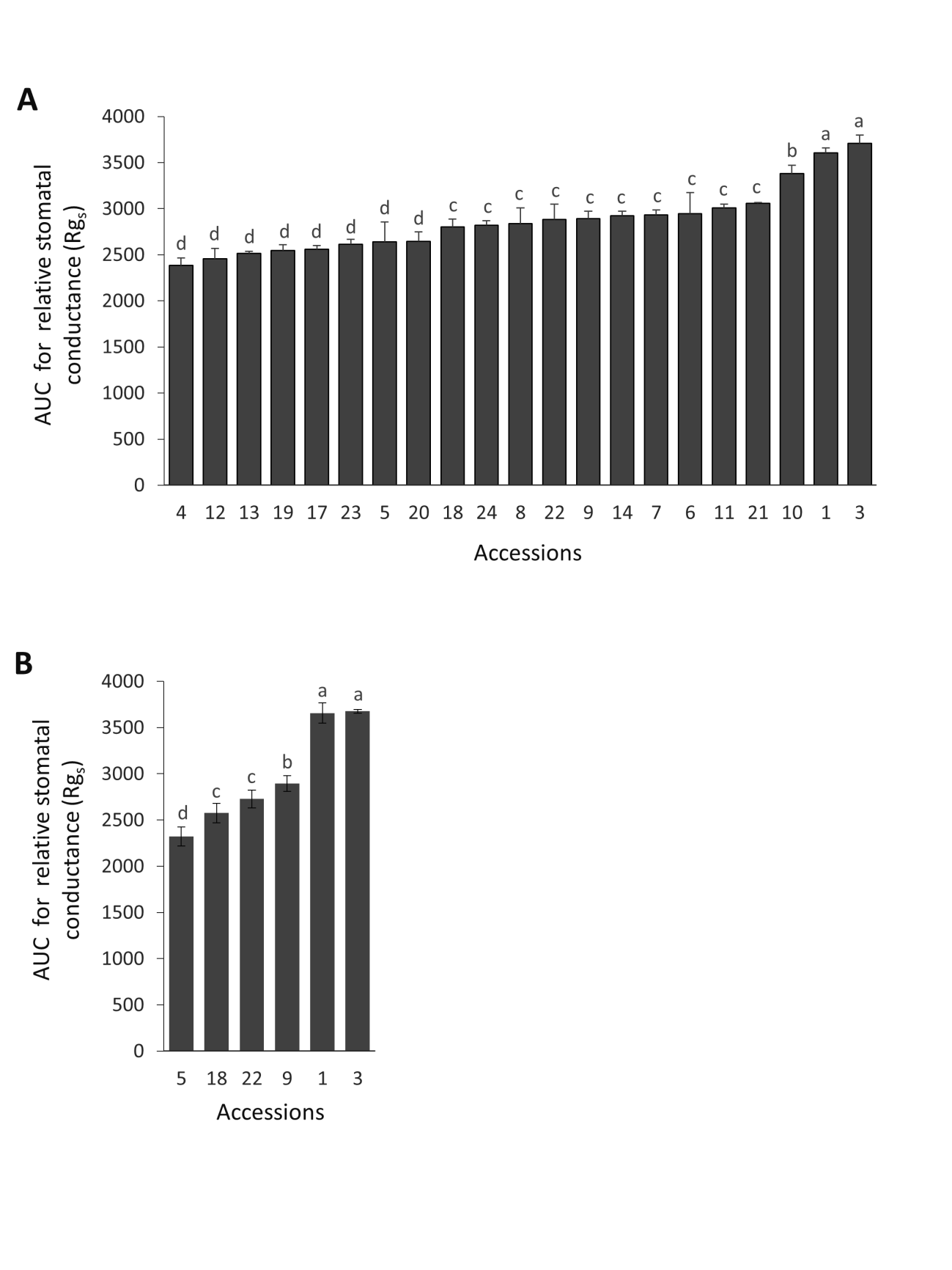


**Figure S8.** Area under the curve (AUC) for relative stomatal conductance (Rg_s_) in 21 *T. crinita* accessions grown under drought conditions in 2018 (A), and six accessions in 2019 (B). Rg_s_ is expressed as percentage of the stomatal conductance (G_s_) in the respective irrigated Controls, as calculated by the formula: Rg_s_ = (g_sDrought_/g_sIrrigated_) × 100; and the AUC was estimated from the graphs in Fig. 6. Bars indicate mean AUC ± standard deviation. Bars with the same letters are not significantly different at p<0.05, DGC test.

**
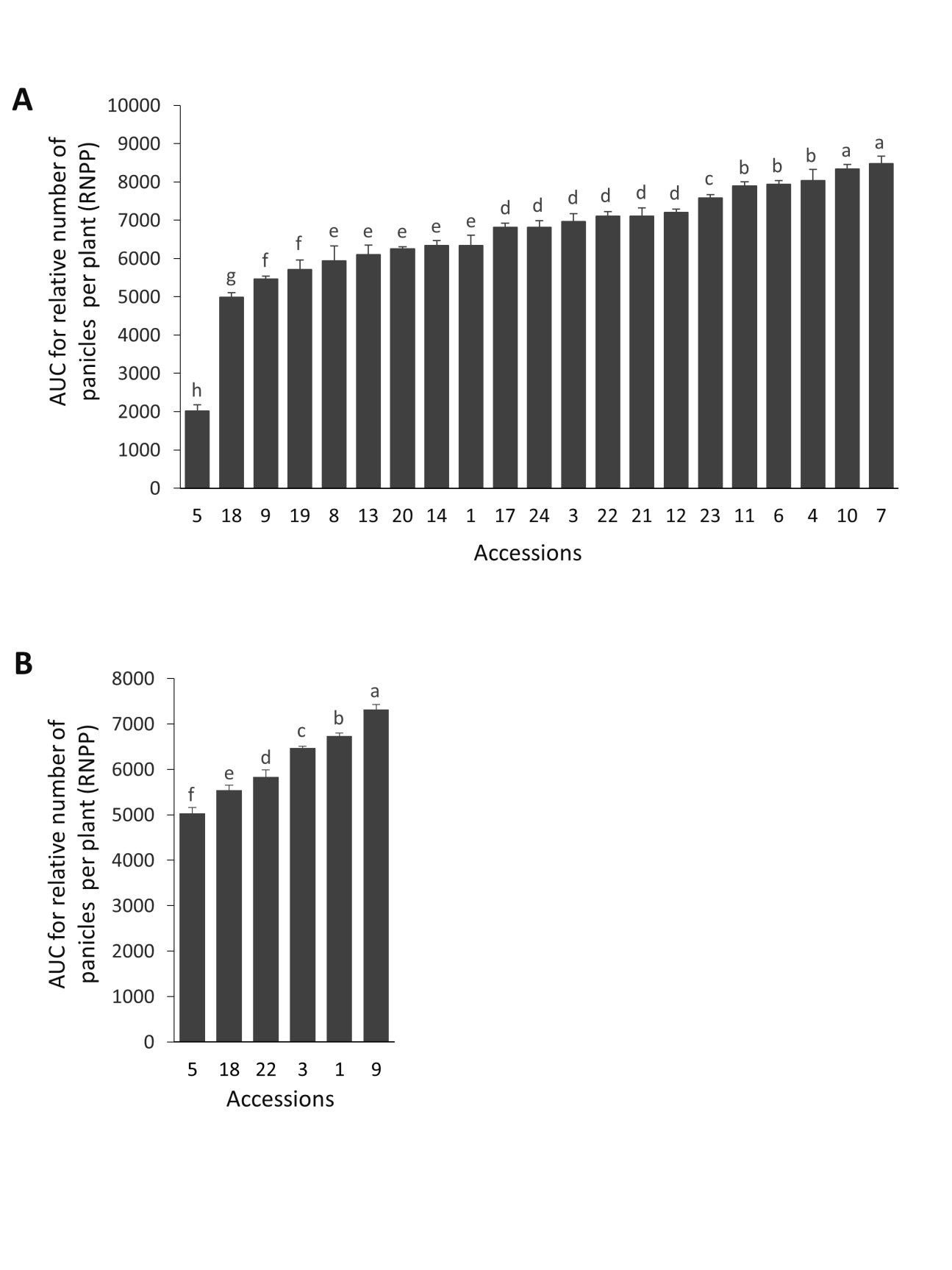
**

**Figure S9.** Area under the curve (AUC) for relative number of panicles per plant (RNPP) in 21 *T. crinita* accessions grown under drought conditions in 2018 (A), and six accessions in 2019 (B). RNPP is expressed as percentage of the number of panicles per plant (NPP) in the respective irrigated Controls, as calculated by the formula: RNPP = (NPP_Drought_/NPP_Irrigated_) × 100; and the AUC was estimated from the graphs in Figure 7. Bars indicate mean AUC ± standard deviation. Bars with the same letters are not significantly different at p<0.05, DGC test.
